# APP lysine 612 lactylation ameliorates amyloid pathology and memory decline in Alzheimer’s disease

**DOI:** 10.1172/JCI184656

**Published:** 2025-01-02

**Authors:** Qiuyun Tian, Junjie Li, Bin Wu, Yayan Pang, Wenting He, Qian Xiao, Jiaojiao Wang, Lilin Yi, Na Tian, Xiuyu Shi, Lei Xia, Xin Tian, Mulan Chen, Yepeng Fan, Boqing Xu, Yuhan Tao, Weihong Song, Yehong Du, Zhifang Dong

**Affiliations:** 1 Growth, Development, and Mental Health of Children and Adolescence Center, Pediatric Research Institute, Ministry of Education Key Laboratory of Child Development and Disorders, National Clinical Research Center for Child Health and Disorders, Chongqing Key Laboratory of Child Neurodevelopment and Cognitive Disorders, Children’s Hospital of Chongqing Medical University, Chongqing, China.; 2 Department of Neurology, The First Affiliated Hospital of Chongqing Medical University, Chongqing Key Laboratory of Neurology, Chongqing, China.; 3 Key Laboratory of Major Brain Disease and Aging Research (Ministry of Education), Chongqing Medical University, Chongqing, China.; 4 Townsend Family Laboratories, Department of Psychiatry, The University of British Columbia, Vancouver, British Columbia, Canada.; 5 Oujiang Laboratory (Zhejiang Lab for Regenerative Medicine, Vision and Brain Health), Institute of Aging, Key Laboratory of Alzheimer’s Disease of Zhejiang Province, Zhejiang Clinical Research Center for Mental Disorders, School of Mental Health and The Affiliated Kangning Hospital, Wenzhou Medical University, Wenzhou, Zhejiang, China.

**Keywords:** Aging, Neuroscience, Alzheimer disease, Neurodegeneration

## Abstract

Posttranslational modification (PTM) of the amyloid precursor protein (APP) plays a critical role in Alzheimer’s disease (AD). Recent evidence reveals that lactylation modification, as a novel PTM, is implicated in the occurrence and development of AD. However, whether and how APP lactylation contributes to both the pathogenesis and cognitive function in AD remains unknown. Here, we observed a reduction in APP lactylation in AD patients and AD model mice and cells. Proteomic mass spectrometry analysis further identified lysine 612 (APP-K612la) as a crucial site for APP lactylation, influencing APP amyloidogenic processing. A lactyl-mimicking mutant (APP_K612T_) reduced amyloid-β peptide (Aβ) generation and slowed down cognitive deficits in vivo. Mechanistically, APP_K612T_ appeared to facilitate APP trafficking and metabolism. However, lactylated APP entering the endosome inhibited its binding to BACE1, suppressing subsequent cleavage. Instead, it promoted protein interaction between APP and CD2-associated protein (CD2AP), thereby accelerating the endosomal-lysosomal degradation pathway of APP. In the APP23/PS45 double-transgenic mouse model of AD, APP-Kla was susceptible to L-lactate regulation, which reduced Aβ pathology and repaired spatial learning and memory deficits. Thus, these findings suggest that targeting APP lactylation may be a promising therapeutic strategy for AD in humans.

## Abstract 2

APP lactylation ameliorates amyloid pathology and memory decline by promoting APP metabolism in Alzheimer’s disease

## Graphical abstract

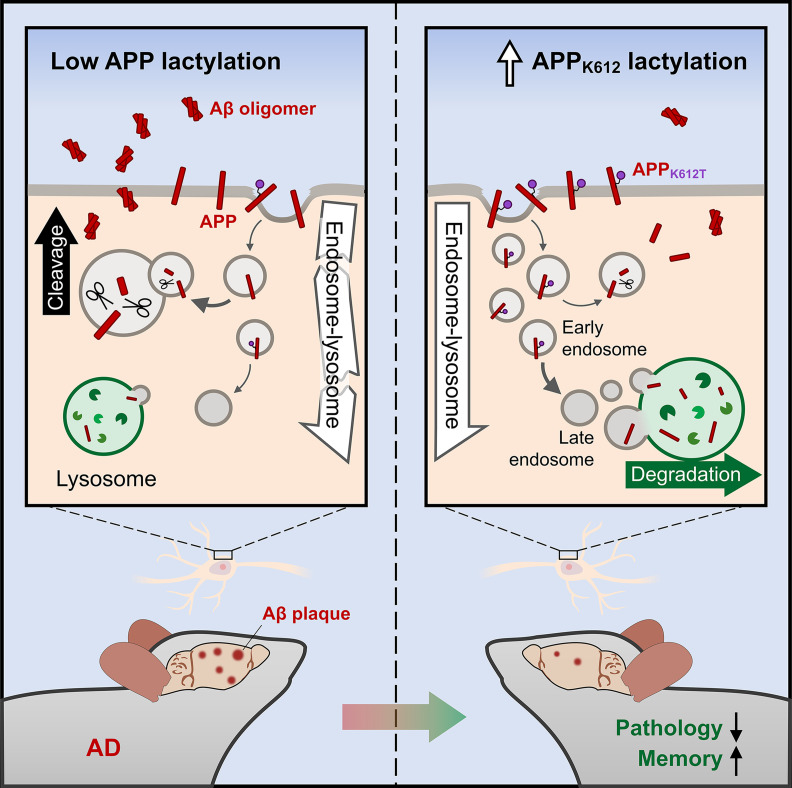

## Introduction

Alzheimer’s disease (AD) stands as one of the most common progressive degenerative diseases of the central nervous system, and its pathogenesis is extremely complex and not fully elucidated. Pathological deposition of amyloid-β (Aβ) peptides is a pivotal pathological hallmark of AD, believed to be a primary driver of subsequent neuronal loss and eventual cognitive decline in AD (1–3). Aβ originates from sequential proteolytic cleavages of the Aβ precursor protein (APP) by β-secretase (BACE1) and γ-secretase, with a major component being presenilin-1 (PS1) (4, 5). A growing body of evidence suggests that protein posttranslational modification (PTM) serves as an effective and rapid regulatory mechanism linking metabolism to protein and cellular function (6), playing a critical role in the pathology of neurological disorders (7, 8). In recent years, more and more studies have revealed that abnormal APP PTM could serve as a key factor affecting APP metabolism and Aβ deposition by regulating processes such as APP hydrolysis and transportation (9–11). APP undergoes various forms of PTMs, including phosphorylation (12, 13), ubiquitination (14, 15), glycosylation (16–18), palmitoylation (19–21), succinylation (22), acetylation (11), and small ubiquitin–related modifier (SUMO) (23, 24). These modifications actively participate in and regulate the pathological processes of AD. Therefore, investigating new PTMs and their associated regulatory mechanisms in APP holds the potential to identify drug targets for the treatment of AD.

Lactate was initially considered as a metabolic byproduct and a potential energy substrate, supporting up to 10% of brain energy metabolism (25). However, recent studies have revealed that lactate can also exert its biological activity through a protein PTM, referred to as lactylation modification (26–29). In 2019, Zhang and colleagues first reported that lactate accumulating during metabolism can serve as a precursor for stimulating histone lactylation at lysine residues (Kla), thereby turning on gene expression to promote homeostasis (26). Subsequent studies have demonstrated that Kla modification is implicated in various cellular functions related to glycolysis (19), macrophage polarization (30, 31), modulation of inflammation (32), and tumor-related diseases (33–35). In addition, Kla has also been reported to have a unique function in the regulation of brain function and the development of brain diseases (35–37). For example, histone H1 Kla occurs in brain cells, and its levels can be regulated by neuronal excitation and social defeat stress (36). During cerebral ischemia, Kla of key proteins in the Ca^2+^ signaling pathway, including Scl25a4, Slc25a5, and Vdac1, may influence mitochondrial function and neuronal injury (38). Levels of both Pan-Kla and H4K12la are increased in the prefrontal cortex and hippocampus of 5×FAD model mice, where H4K12La is enriched in the promoter of glycolytic genes and promotes transcription of glycolytic genes (29). However, it remains unclear whether APP, a key gene implicated in AD pathogenesis, undergoes Kla modification (APP-Kla) and how it contributes to the development of AD.

In this study, we identified that the decrease in K612 lactylation is a critical pathological change leading to APP amyloidogenic processing in AD brains using mass spectroscopy and a lactyl-mimicking mutant (APP_K612T_). Furthermore, we observed that APP_K612T_ promoted APP endosomal-lysosomal degradation by enhancing the interaction between APP and CD2AP in cellular endosomes. Finally, our findings indicated that APP-Kla was susceptible to modulation by L-lactate, which decreased Aβ burden and improved spatial learning and memory in the AD model of APP23/PS45 double-transgenic mice. Our study thus suggests that promoting APP-Kla may represent a promising therapeutic strategy for treating AD.

## Results

### Reduced expression level of APP lactylation modification in AD.

We first detected the protein expression levels of pan-lysine lactylation (Pan-Kla) in AD. The results showed that Pan-Kla protein levels in the hippocampus and frontal cortex tissues of patients with AD were not significantly changed compared with age-matched control participants (Supplemental Figure 1A; supplemental material available online with this article; https://doi.org/10.1172/JCI184656DS1). Similar to the findings in patients with AD, the Pan-Kla expression levels remained unchanged in the hippocampus and cortex tissues of 6-month-old APP23/PS45 double-transgenic AD model mice compared with age-matched WT mice (Supplemental Figure 1B). Additionally, to observe the localized expression of Pan-Kla in neuronal cells, immunofluorescence staining experiments revealed that Pan-Kla significantly colocalized with microglia (CD11B), astrocytes (GFAP), and neurons (NEUN) in hippocampus tissue sections from 6-month-old WT mice and APP23/PS45 double-transgenic AD mice (Supplemental Figure 1, C–E). This indicates that Pan-Kla is widely expressed in hippocampal neuronal cells of AD mice and may play a potential function in AD.

**Figure 1:**
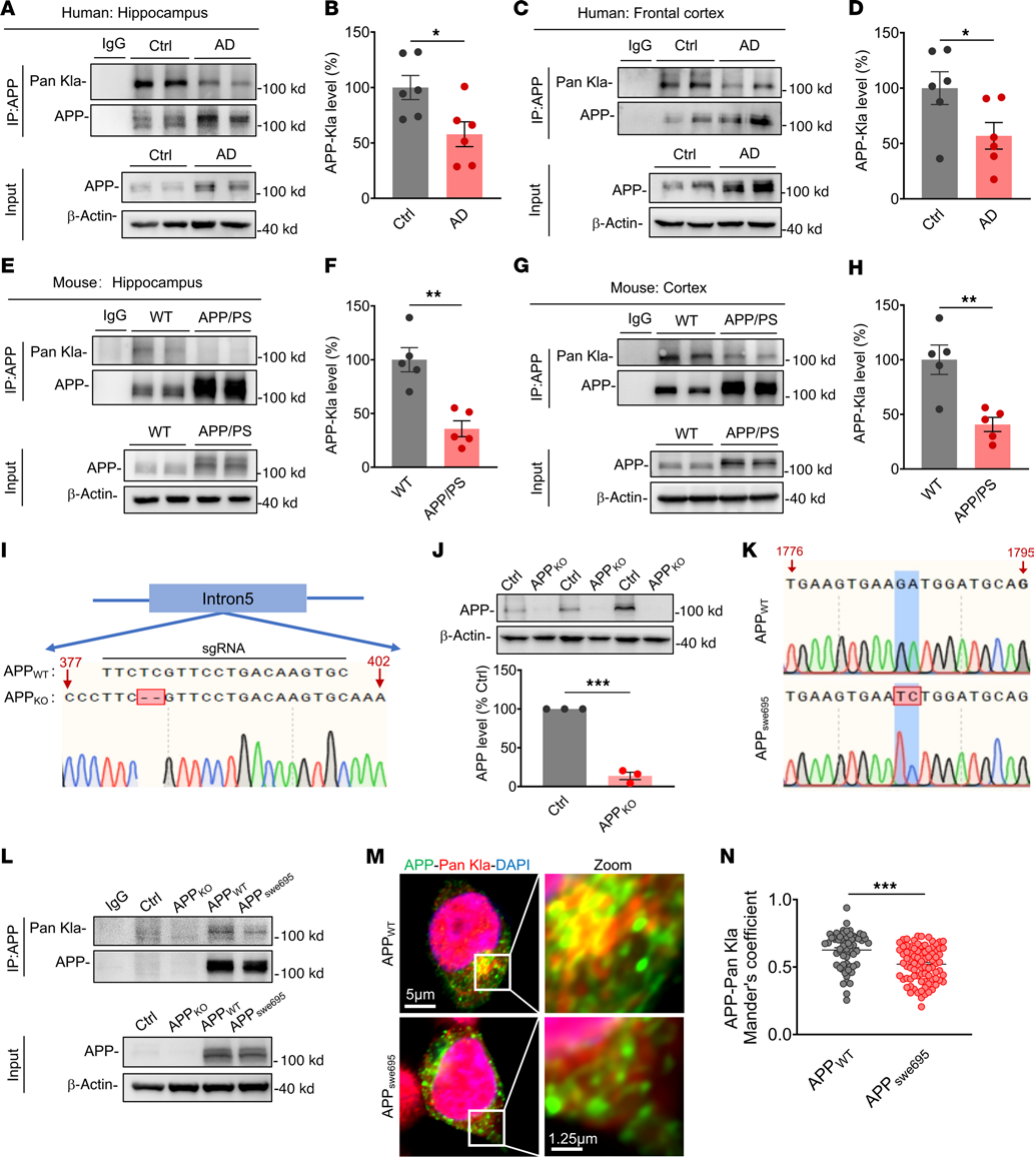
Reduced expression level of APP lactylation modification in AD. (**A**–**D**) Hippocampus (**A** and **B**) and frontal cortex (**C** and **D**) tissue lysates were immunoprecipitated with APP antibody, followed by immunoblot analysis with Pan-Kla antibody to detect APP-Kla expression levels in patients with AD and age-matched people in the control group (*n* = 6 in each group). (**E**–**H**) Hippocampus (**E** and **F**) and cortex (**G** and **H**) tissue lysates were immunoprecipitated with APP antibody, followed by immunoblot analysis with Pan-Kla antibody to detect APP-Kla expression levels in WT and APP23/PS45 mice at the age of 6 months (*n* = 5 in each group). (**I**) Genomic DNA sequences of APP locus in APP_KO_ HEK293 cells. (**J**) The relative protein levels of APP were assessed by Western blot in APP_KO_ cells and HEK293 cells (*n* = 3 in each group). (**K**) Sequencing map of the APP_WT_ and APP_swe695_ mutation site. (**L**) Cell lysates were immunoprecipitated with APP antibody, followed by immunoblot analysis with Pan-Kla antibody to detect APP-Kla expression levels in APP_WT_ and APP_swe695_ groups (*n* = 3 in each group). (**M** and **N**) Representative confocal fluorescence images of APP costained with Pan-Kla in APP_WT_ and APP_swe695_ groups (**M**), as well as the colocalization of APP with Pan-Kla in multiple confocal images quantified by calculating the Manders’ overlap coefficient (**N**) (*n* > 60 cells in each group; scale bars: 5 μm (left) and 1.25 μm (right)). Data were presented as mean ± SEM, *P < 0.05, **P < 0.01, ***P < 0.001 by 2-tailed unpaired Student’s *t* test (**B**–**D**, **F**, **G**, **J**, and **N**).

Next, we focused on detecting the expression level of lactylation modification of APP (APP-Kla) in AD. The results showed that APP-Kla protein levels in the hippocampus and frontal cortex tissues of patients with AD were significantly reduced compared with age-matched participants in the control group (Figure 1, A–D). Similar to the findings in patients with AD, the expression levels of APP-Kla were significantly decreased in the hippocampus and cortex tissues of 6-month-old APP23/PS45 double-transgenic AD model mice compared with age-matched WT mice (Figure 1, E–H). To further explore the APP-Kla without endogenous interference, we constructed APP knockout (APP_KO_) cell lines in HEK293 cells by CRISPR/Cas9 technology (Figure 1I). The protein expression of APP was almost completely abolished in APP_KO_ cells compared with control (ctrl) cells, indicating a highly efficient APP knockout (Figure 1J). Next, we constructed the APP_WT_ or APP_swe695_ mutant plasmids (Figure 1K) and transfected into APP_KO_ cell lines. We found that the APP-Kla level was significantly reduced in APP_swe695_ compared with APP_WT_ (Figure 1L). To more directly detect the expression distribution of APP and Pan-Kla, we performed immunofluorescence assays and found that the colocalization coefficient of APP with Pan-Kla in the APP_swe695_ group was significantly lower than that in the APP_WT_ group (Figure 1, M and N). Taken together, these results suggest that the expression level of APP-Kla is reduced in AD.

### APP-K612la reduces APP amyloidogenic processing in vitro.

**Figure 2:**
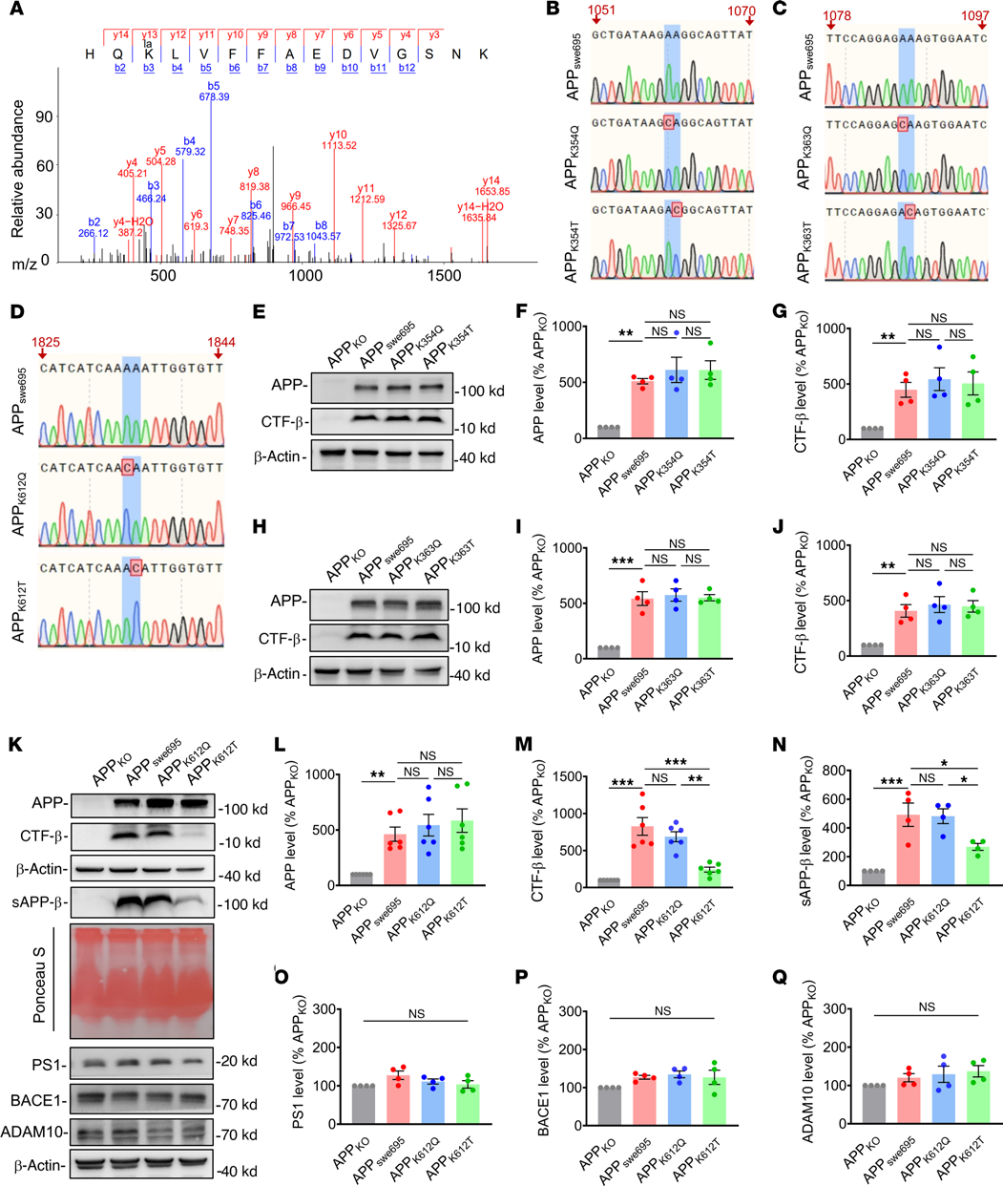
APP-K612la reduced APP amyloidogenic processing in vitro (**A**) LC-MS/MS spectra of the lactylated peptides of APP-K612. (**B**–**D**) Sequencing map of the APP lactylation/delactylation in K354 (**B**), K363 (**C**), and K612 (**D**) mimic lysine mutation site. Blue-shaded lines and red-shaded boxes represent lysine mutation sites. (**E**–**G**) The relative protein levels of APP (**E** and **F**) and CTF-β (**E** and **G**) were assessed by Western blot in APP_KO_ cells transfected with APP_swe695_, APP_K354Q_, and APP_K354T_ mutant plasmids (*n* = 4 in each group). (**H**–**J**) The relative protein levels of APP (**H** and **I**) and CTF-β (**H** and **J**) were assessed by Western blot in APP_KO_ cells transfected with APP_swe695_, APP_K363Q_, and APP_K363T_ mutant plasmids (*n* = 4 in each group). (**K**–**Q**) The relative protein levels of APP (**K** and **L**), CTF-β (**K** and **M**), sAPP-β (**K** and **N**), ADAM10 (**K** and **O**), BACE1 (**K** and **P**), and PS1 (**K** and **Q**) were assessed by Western blot in APP_KO_ cells transfected with APP_swe695_, APP_K612Q_, and APP_K612T_ mutant plasmids (*n* = 4–6 in each group). Data were presented as mean ± SEM, *P < 0.05, and ***P < 0.001. 1-way ANOVA, followed by Tukey’s multiple comparisons test (**F**, **G**, **I**, **J**, and **L**–**Q**).

Further mass spectrometry analysis predicted 3 possible lysine sites including APP_K354_, APP_K363_, and APP_K612_, where APP may undergo lactylation modification (Figure 2A and Supplemental Figure 2, A and B). To mimic lactylation or delactylation of APP, we replaced lysine in APP_swe695_ with threonine (39) (APP_K354T_, APP_K363T_, and APP_K612T_) or glutamine (40) (APP_K354Q_, APP_K363Q_, and APP_K612Q_), respectively (Supplemental Figure 2C and Figure 2, B–D). Subsequently, we constructed the plasmids and transfected into APP_KO_ cell lines. Neither APP_K354Q_ nor APP_K354T_ affected the protein expression levels of APP and CTF-β compared with APP_swe695_ (Figure 2, E–G). Similarly, both APP_K363Q_ and APP_K363T_ also did not change the protein expression levels of APP and CTF-β (Figure 2, H–J). However, the protein expression levels of CTF-β and sAPP-β, but not APP and its proteolytic enzymes, including BACE1, ADAM10, and PS1 were markedly reduced in APP_K612T_ (Figure 2, K–Q). In addition, we found that the protein expression levels of CTF-β, sAPP-β, and APP-related cleavage enzymes such as BACE1, PS1, and ADAM10 were not significantly changed in APP_K612Q_ compared with APP_swe_ (Figure 2, K–Q). However, the protein expression levels of CTF-α and sAPP-α were significantly reduced in both APP_K612Q_ and APP_K612T_ (Supplemental Figure 2, D and E). Collectively, these results suggest that the lactyl-mimicking mutant APP_K612T_ may affect APP trafficking and metabolism, but it does not impact overall APP expression.

To investigate whether APP-K612 undergoes lactylation modification and its potential role, we treated APP_KO_ cells transfected with APP plasmids using L-lactate and analyzed protein levels via Western blot. The results revealed that L-lactate treatment significantly reduced the protein expression levels of CTF-β in the APP_swe_ group, but did not produce this effect in the APP_K612Q_ group (Supplemental Figure 3, A–F). This suggests that the APP-K612 locus may play a critical role in the lactylation modification of APP. Inhibition of lactylation at this site did not alter CTF-β levels, despite L-lactate treatment, indicating resistance to rescue attempts.

### APP-K612la reduces Aβ generation in vivo.

**Figure 3:**
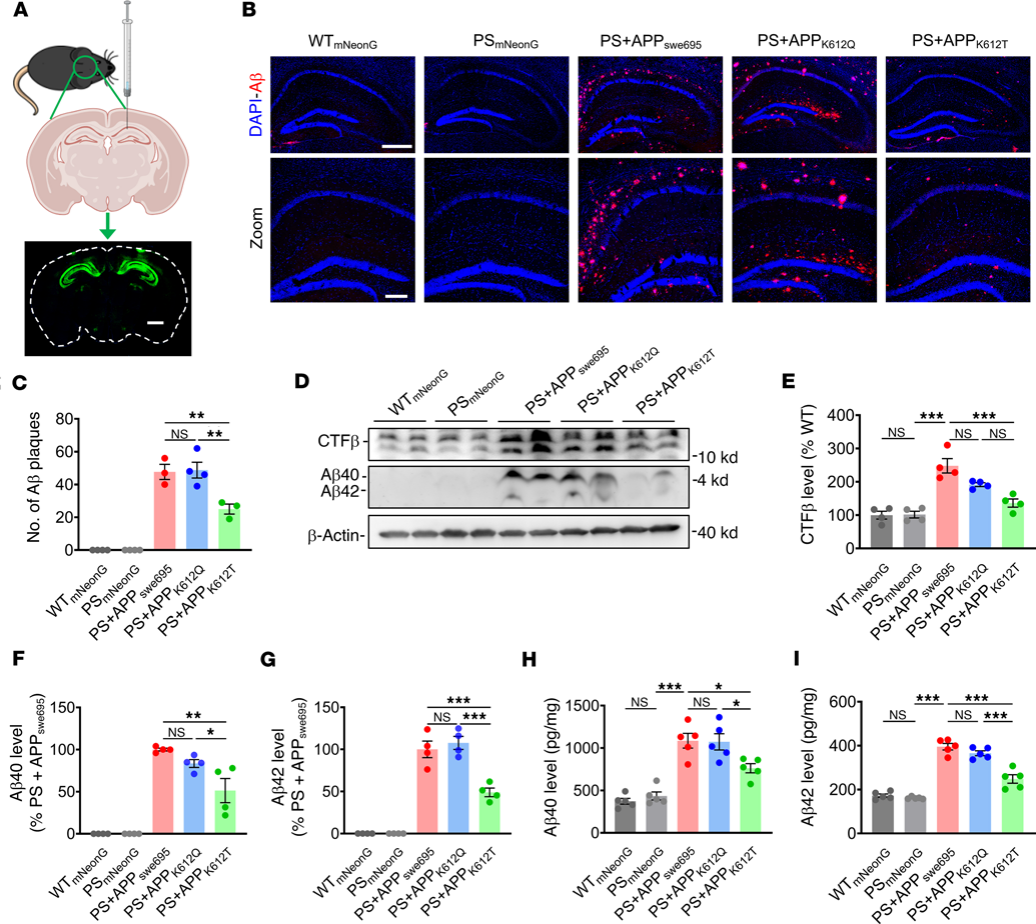
APP-K612la reduced Aβ generation in vivo. (**A**) Brain stereotactic injection of mouse bilateral hippocampal CA1 region with virus and immunofluorescence signaling in brain slices (Scale bar: 1,000 μm). (**B** and **C**) Representative confocal fluorescence images of Aβ (**B**), as well as the number of Aβ plaques (**C**) in the hippocampal region of WT and PS45 mice injected with APP_swe695_, APP_K612Q_, and APP_K612T_ viruses at the age of 5 months (Scale bar: 500 μm [up] and 200 μm [down], *n*=3–4 in each group). (**D**–**G**) The relative protein levels of CTF-β (**D** and **E**), Aβ40 (**D** and **F**) and Aβ42 (**D** and **G**) were assessed by Western blot (*n* = 4 in each group). (**H** and **I**) Generation of Aβ40 (**H**) and Aβ42 (**I**) as measured by ELISA (*n* = 5 in each group). Data were presented as mean ± SEM, *P < 0.05, **P < 0.01, ***P < 0.001. 1-way ANOVA, followed by Tukey’s multiple comparisons test (**C** and **E**–**I**).

To determine the role and therapeutic potential of APP lactylation in the pathogenesis of AD, we generated adeno-associated virus (AAV) carrying APP_swe695_, APP_K612Q_, or APP_K612T_. Three-month-old PS45 mice were microinjected with AAV into the bilateral hippocampal CA1 regions, and the Aβ pathology and spatial learning and memory were determined 2 months after microinjection (Supplemental Figure 4A). Immunofluorescence staining showed that AAV successfully infected the bilateral hippocampal CA1 region of mice (Figure 3A). Next, we observed the distribution of Aβ plaque expression in the mouse hippocampus by immunofluorescence staining. The results showed that the number of Aβ plaques was significantly increased in the hippocampus of APP_swe695_-treated mice compared with WT and PS45 mice, but APP_K612T_ treatment significantly reduced the number of Aβ plaques (Figure 3, B and C). In addition, the expressions of CTF-β, Aβ40, and Aβ42 were significantly reduced in APP_K612T_-treated PS45 mice compared with APP_swe695_ mice (Figure 3, D–G). To better understand Aβ expression, we further quantified Aβ40 and Aβ42 levels using ELISA. The results showed that APP_K612T_ mice exhibited significantly lower levels of both Aβ40 and Aβ42 in the hippocampus compared with APP_swe_ and APP_K612Q_ mice (Figure 3, H and I). Nevertheless, we did not observe any changes in protein expression in APP, BACE1, and PS1 (Supplemental Figure 4, B–E).

### APP-K612la ameliorates synaptic and memory impairments in vivo.

**Figure 4:**
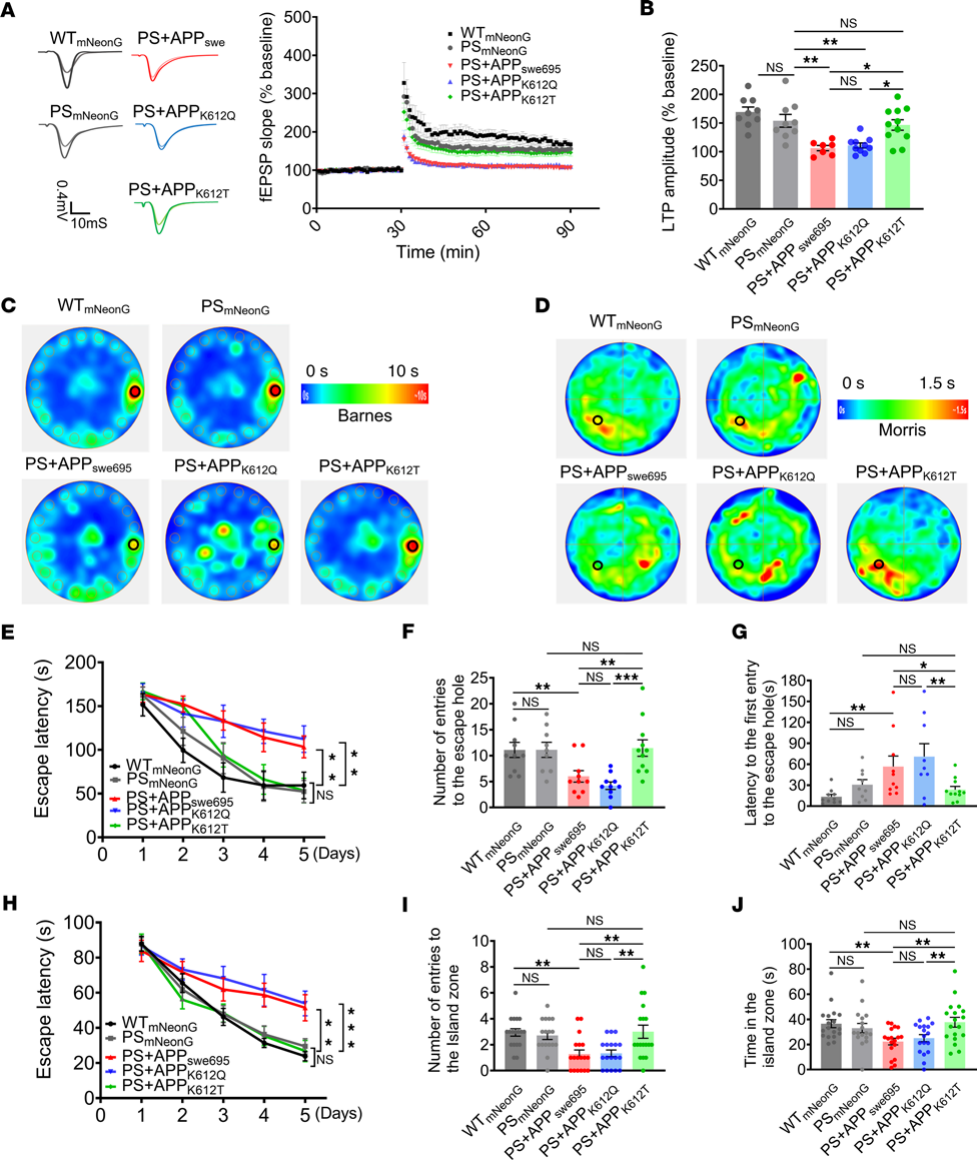
APP-K612la ameliorated synaptic and memory impairments in vivo. (**A** and **B**) Hippocampal CA1 LTP recorded from mouse brain slices (**A**) and the bar graphs of the average percentage changes in the fEPSP slope 55–60 minutes after TBS delivery (**B**) (*n* = 7–11 slices from 3–4 mice in each group). (**C**) Average heatmap during memory retrieval in the Barnes maze test. (**D**) Average heatmap during memory retrieval in the Morris water maze test. (**E**) The latency to the escape hole during spatial learning in the Barnes maze paradigm (*n* = 9–11 in each group). (**F** and **G**) Correct number of finding the escape hole (**F**) and the latency to finding the escape hole (**G**) during memory retrieval in the Barnes maze test (*n* = 9–11 in each group). (**H**) The latency for finding the hidden island during spatial learning in the Morris water maze test (*n* = 16–18 in each group). (**I** and **J**) Number of entries to the island zone (**I**) and the time in the hidden platform quadrant zone during spatial learning (**J**) in the Morris water maze test (*n* = 16–18 in each group). Data were presented as mean ± SEM, *P < 0.05, **P < 0.01, ***P < 0.001. 1-way ANOVA, followed by Tukey’s multiple comparisons test (**B**, **F**, **G**, **I**, and **J**) or 2-way ANOVA (**E** and **H**).

Hippocampal LTP is usually recognized as a cellular mechanism for spatial learning and memory. Next, we investigated the changes of hippocampal CA1 LTP in PS45 mice microinjected with AAV. The results showed that the hippocampal CA1 LTP was impaired in APP_swe695_ mice compared with PS45 and WT mice. As expected, mice treated with APP_K612T_ recovered from LTP deficits (Figure 4, A and B). To directly examine the effect of APP lactylation on cognitive function in vivo, the Barnes maze and Morris water maze tests were introduced to measure hippocampus-dependent spatial learning and memory (Figure 4, C and D). The results of the Barnes maze test showed that the spatial learning ability of APP_swe695_ mice was impaired, as reflected by a longer latency to find the escape hole, compared with WT mice. APP_K612T_, but not APP_K612Q_, mice displayed a significant improvement in spatial learning compared with APP_swe695_ mice (Figure 4E). In the probe trial, the spatial memory retrieval was impaired in APP_swe695_ mice, as evidenced by the reduced correct number to find the escape hole and longer latency to the escape hole, compared with WT mice, while the spatial memory retrieval restored to WT control level in APP_K612T_-treated mice (Figure 4, F and G). Similarly, the Morris water maze test also showed that spatial learning and memory retrieval were impaired in APP_swe695_ mice compared with WT mice, whereas these impairments were ameliorated in APP_K612T_ mice (Figure 4, H–J). In addition, we analyzed the swimming speed of mice in the Morris water maze test and found no significant differences in any of them (Supplemental Figure 5, A and B). To exclude the interference of mouse spontaneous locomotion and exploratory behaviors on learning and memory, we conducted the Open-field test and Elevated plus maze experiments before the learning and memory behavioral tests. The results showed that there was no significant difference in the total distance traveled among these groups (Supplemental Figure 5, C and D), as well as the number of entries to the open arm zone between the groups of mice (Supplemental Figure 5, E and F). Taken together, these results demonstrate that APP-K612la significantly reduces Aβ generation and ameliorates synaptic and cognitive impairments in vivo.

### APP-K612la regulates transcription associated with APP trafficking and metabolism in the hippocampus.

**Figure 5:**
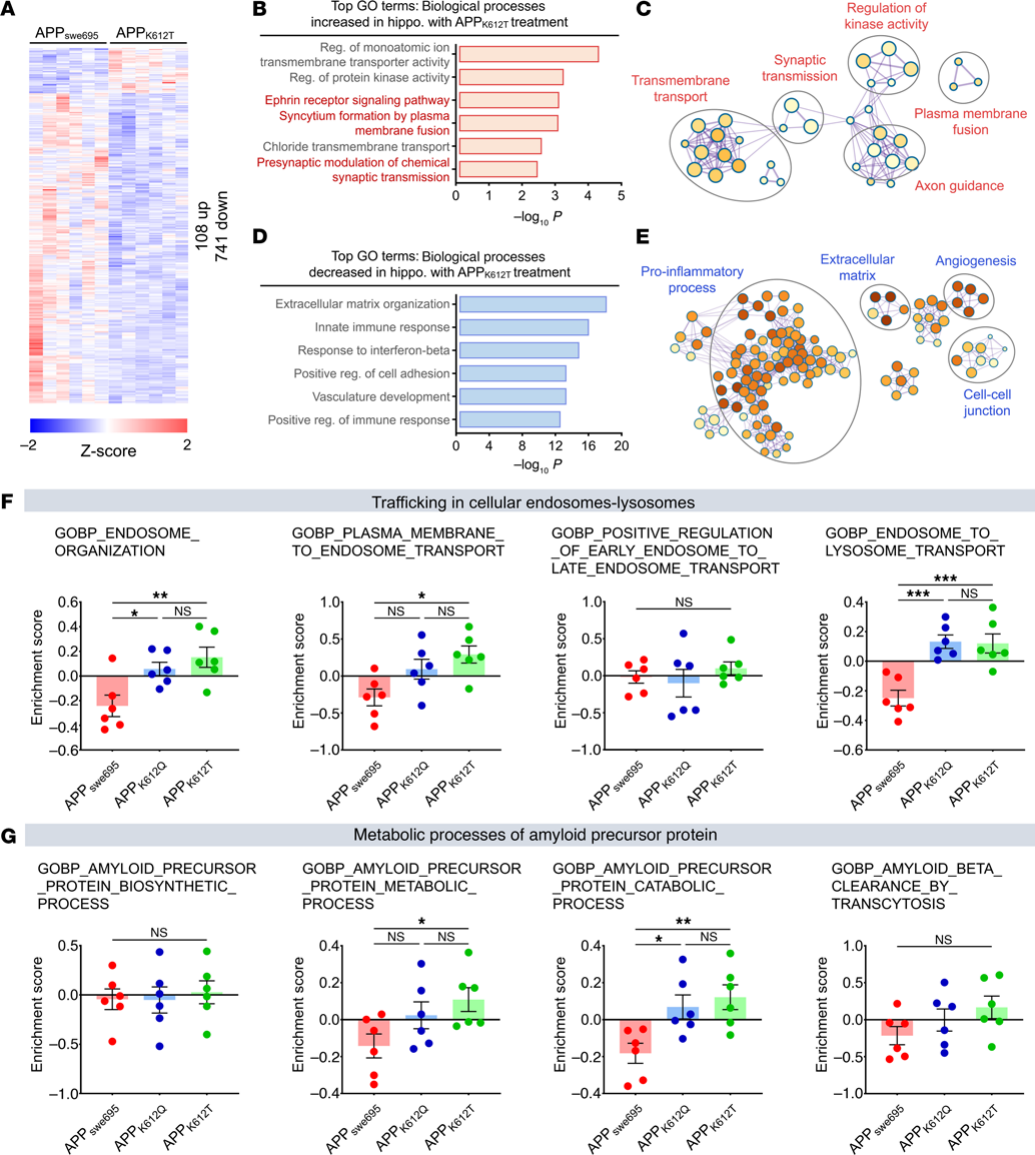
APP-K612la regulated transcription associated with APP metabolism in hippocampus. (**A**) Significant DEGs were identified by RNA-seq analysis of hippocampal tissues of AAV-APP_swe695_, APP_K612T_ microinjected mice (log_2_ FC = 1.2, *P* < 0.05, *n* = 6 in each group). (**B** and **C**) Top GO terms and GO enrichment map associated with upregulated DEGs in **A**. (**D** and **E**) Top GO terms and GO enrichment map associated with downregulated DEGs in **A**. (**F**) Enrichment scores for related endosomal-to-lysosomal transport in each group of hippocampal transcripts were assessed using GSVA (*n* = 6 in each group). (**G**) Enrichment scores for relevant APP metabolism in each group of hippocampal transcripts were assessed using GSVA (*n* = 6 in each group). Data were presented as mean ± SEM, *P < 0.05, **P < 0.01, ***P < 0.001. 1-way ANOVA, followed by Tukey’s multiple comparisons test (**F** and **G**).

We next isolated hippocampal tissues from mice after AAV microinjection and performed unbiased transcriptome-wide RNA-seq analysis to assess the effects of APP-K612la on AD mice. Differentially expressed genes (DEGs) were analyzed in the hippocampus of APP_K612T_ and APP_swe695_ mice, revealing 849 DEGs (Figure 5A). Gene Ontology (GO) and GO enrichment map analysis of 108 upregulated DEGs indicated that APP-K612la mice were enriched in pathways related to synaptic transmission, transmembrane transport, and axon guidance (Figure 5, B and C). Conversely, GO enrichment analysis of the 741 downregulated DEGs showed a focus on proinflammatory processes (Figure 5, D and E). These results suggest that APP-K612la may be associated with APP trafficking metabolism. We further explored biological changes after APP delactylation in AD brains by analyzing DEGs in the hippocampus of APP_K612Q_ and APP_swe695_ mice. In total, 1,134 DEGs were identified (Supplemental Figure 6). Analysis revealed that APP_K612Q_ mice showed downregulation of transcription genes related to abnormal lipid metabolism, cellular proliferation, and immune response compared with APP_swe695_ mice (Supplemental Figure 6, B–E).

Given that APP metabolism involves hydrolysis by secreted enzymes and trafficking via organelle membranes (41–43), we next assessed the activity of related pathways using gene set variation analysis (GSVA). APP-K612la mice exhibited higher enrichment scores for endosomal-associated trafficking pathways and more active APP metabolism processes in the hippocampus (Figure 5, F and G). Collectively, these results suggest that APP-K612la may regulate transcriptional programs associated with APP trafficking and metabolism in AD mice.

### APP-K612la enhances APP trafficking from the plasma membrane to the endosomes and lysosomes.

**Figure 6:**
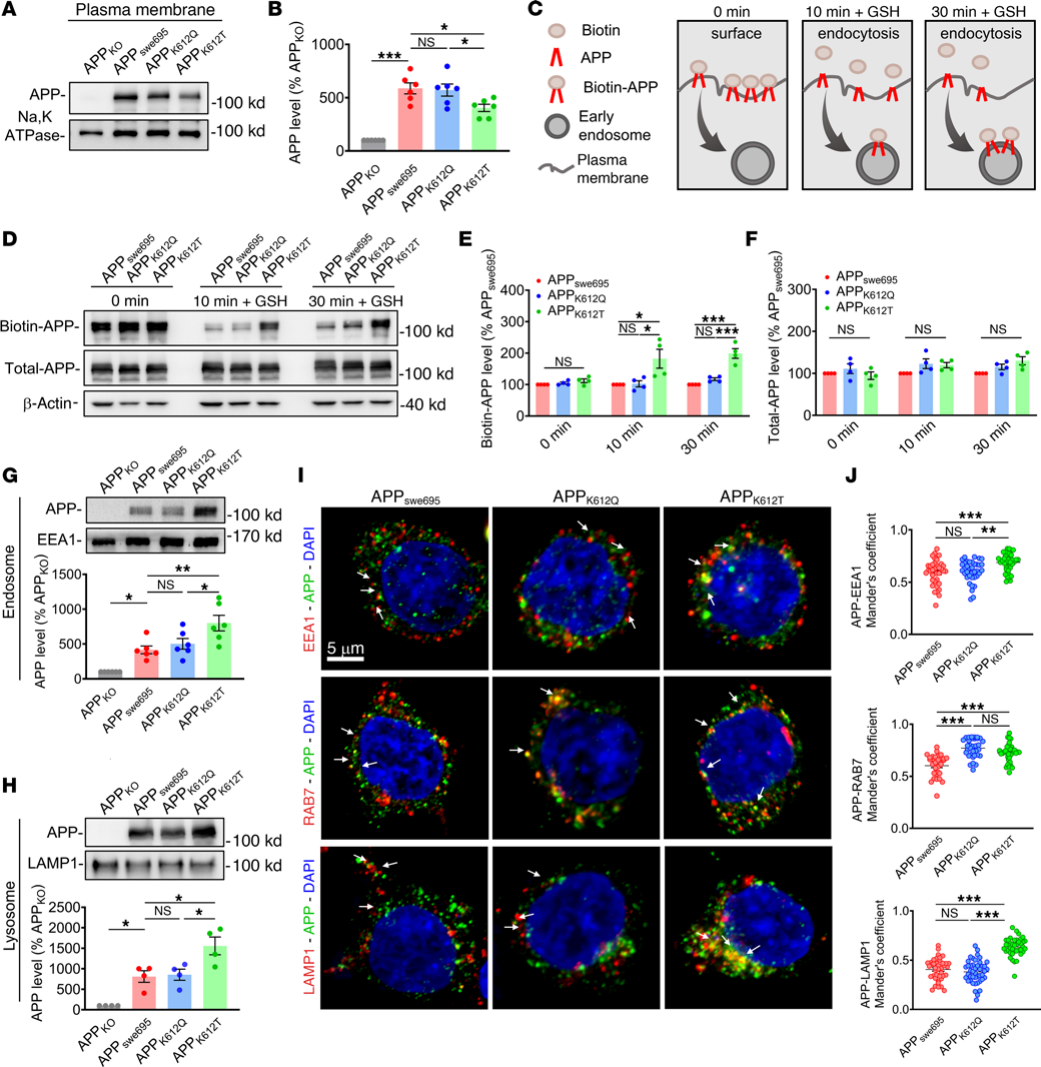
APP-K612la enhanced APP trafficking from the plasma membrane to the endosomes and lysosomes. (**A** and **B**) The relative protein levels of APP in the plasma membrane were assessed by Western blot in APP_KO_ cells transfected with APP_swe695_, APP_K612Q_, and APP_K612T_ mutant plasmids (*n* = 6 in each group). (**C**) Schematic representation of APP endocytosis assay using surface protein biotinylation. The biotinylated surface was chased for 10 minutes or 30 minutes, and then surface biotin was removed with non–cell-permeable GSH to detect endocytosed biotinylated proteins. (**D**–**F**) Biotinylation experiments were performed in APP_KO_ cells transfected with APP_swe695_, APP_K612Q_, and APP_K612T_ mutant plasmids, and the relative endocytosed protein levels of biotin-APP and total-APP were assessed by Western blot (*n* = 4 per group). (**G**) The relative protein levels of APP in endosomes were assessed by Western blot in APP_KO_ cells transfected with APP_swe695_, APP_K612Q_, and APP_K612T_ mutant plasmids (*n* = 6 in each group). (**H**) The relative protein levels of APP in lysosomes were assessed by Western blot in APP_KO_ cells transfected with APP_swe695_, APP_K612Q_, and APP_K612T_ mutant plasmids (*n* = 4 in each group). (**I** and **J**) Representative confocal fluorescence images of APP costained with EEA1, RAB7, and LAMP1 in APP_swe695_, APP_K612Q_, and APP_K612T_ groups (**I**), as well as the colocalization of APP with EEA1, RAB7, and LAMP1 in multiple confocal images quantified by calculating the Manders’ overlap coefficient (**J**) (*n* > 30 cells in each group; scale bars: 5 μm.). Data were presented as mean ± SEM, *P < 0.05, **P < 0.01, ***P < 0.001. 1-way ANOVA, followed by Tukey’s multiple comparisons test (**B**, **E**–**H**, and **J**).

Numerous studies have shown that APP on the cell surface rapidly internalizes into the early endosomes, where it is transported to the late endosomes and lysosomes for degradation (44). Therefore, we next obtained plasma membrane fractions (Supplemental Figure 7A) and examined the expression change of APP on the plasma membrane. We observed a significant decrease in the APP expression on the plasma membrane in APP_K612T_ cells compared with APP_swe695_ cells (Figure 6, A and B). In addition, a biotinylated APP endocytosis assay was also performed to confirm the effect of APP-K612la on APP endocytosis (Figure 6C). The results showed that the amount of endocytosed (biotinylated) APP was increased in APP_K612T_, but not APP_K612Q_, compared with APP_swe695_ cells, and this increase persisted for at least 30 minutes (Figure 6, D–F). To further determine the destination of the endocytosed APP, we isolated the cellular endosomal proteins (Supplemental Figure 7B) and lysosomal proteins (Supplemental Figure 7C) and found that the APP was significantly increased in both endosomal and lysosomal fractions in APP_K612T_, but not APP_K612Q_ cells, compared with APP_swe695_ cells (Figure 6, G and H). To directly observe the trafficking localization of APP, we used confocal imaging to detect the APP distribution in the early endosomes, late endosomes, and lysosomes. Immunofluorescence results showed that the colocalization of APP with EEA1, RAB7, and LAMP1 was significantly increased in APP_K612T_ compared with APP_swe695_ cells (Figure 6, I and J). Taken together, these findings suggest that APP-K612la promotes APP trafficking from the plasma membrane to the endosomes and lysosomes.

### APP-K612la promotes the APP endosomal-lysosomal degradation process associated with CD2AP.

**Figure 7:**
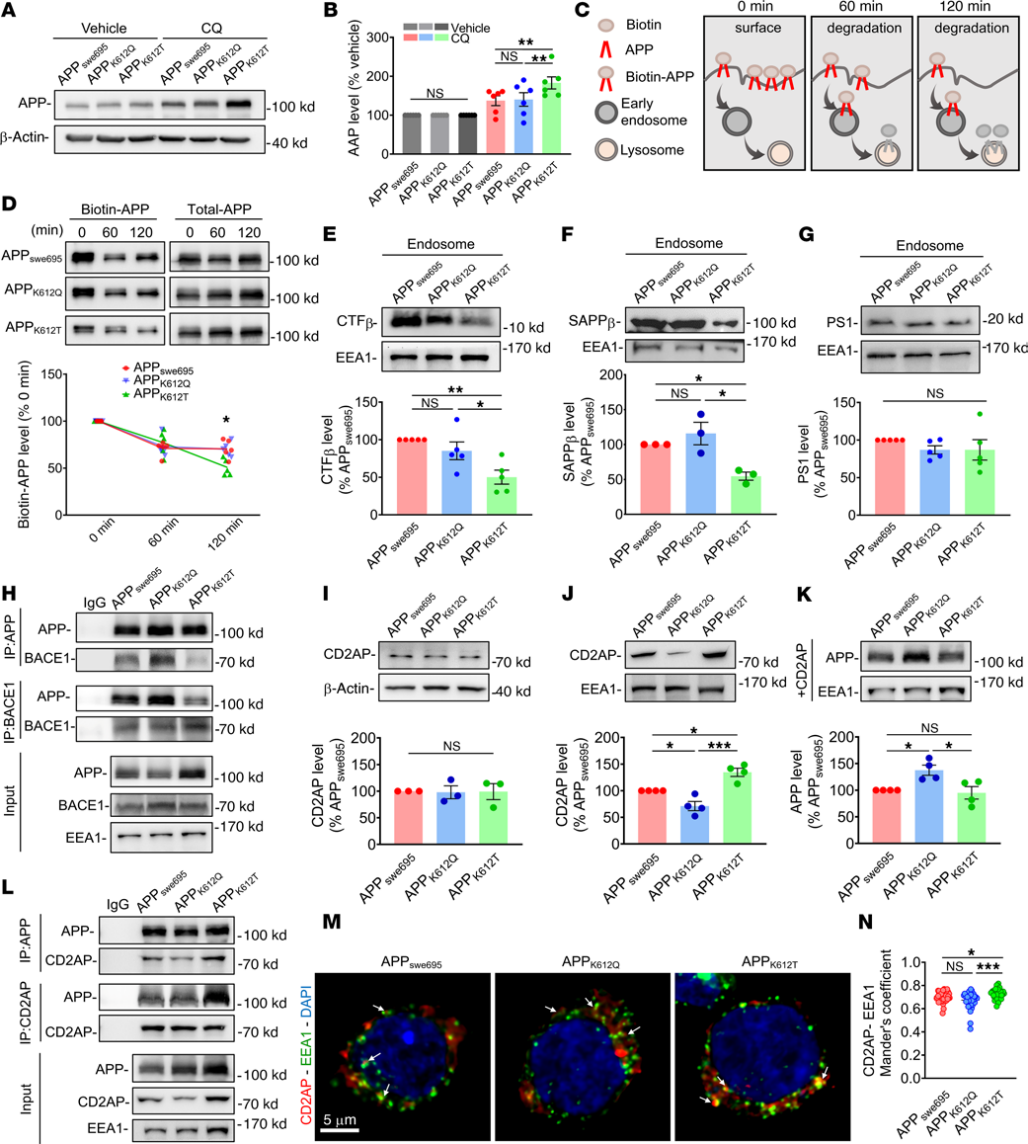
APP-K612la promoted the endosomal-lysosomal degradation process via CD2AP. (**A** and **B**) The relative protein levels of APP treated with chloroquine (CQ, 50 nM) for 24 hours were assessed by Western blot in APP_KO_ cells transfected with APP_swe695_, APP_K612Q_, and APP_K612T_ mutant plasmids (*n* = 4 in each group). (**C**) Schematic representation of APP degradation assay using surface protein biotinylation. (**D**) Degradation of surface biotinylated APP (Biotin-APP, 0 minutes) chased for 60 minutes and 120 minutes in cells (*n* = 5 in each group). (**E**–**G**) The relative protein levels of CTF-β (**E**), sAPP-β (**F**), and PS1 (**G**) in endosomes were assessed by Western blot (*n* = 3–5 in each group). (**H**) Interactions among APP_swe695_, APP_K612Q_, and APP_K612T_ group APP and BACE1 proteins were detected by coimmunoprecipitation in endosomal protein lysates (*n* = 3 per group). (**I**) The relative protein levels of CD2AP were assessed by Western blot in cells (*n* = 4 in each group). (**J**) The relative protein levels of CD2AP in endosomes were assessed by Western blot in cells (*n* = 4 in each group). (**K**) Overexpression of CD2AP plasmid cotransfected with APP_swe695_, APP_K612Q_, and APP_K612T_ mutant plasmids in APP_KO_ cells, and the relative protein levels of APP in endosomes were assessed by Western blotting (*n* = 4 per group). (**L**) Interactions among APP_swe695_, APP_K612Q_, and APP_K612T_ group APP and CD2AP proteins were detected by coimmunoprecipitation in endosomal protein lysates (*n* = 3 per group). (**M** and **N**) Representative confocal fluorescence images of APP costained with CD2AP in APP_swe695_, APP_K612Q_, and APP_K612T_ groups (**M**), as well as the colocalization of APP with CD2AP in multiple confocal images quantified by calculating the Manders’ overlap coefficient (**N**) (*n* > 30 cells in each group; scale bar: 5 μm.). Data were presented as mean ± SEM, *P < 0.05, **P < 0.01, ***P < 0.001. 1-way ANOVA, followed by Tukey’s multiple comparisons test (**B**, **E**–**G**, **I**–**K**, and **N**) or 2-way ANOVA (**D**).

To determine the effect of the lysosomal degradation pathway on APP metabolism, we treated cells with chloroquine (CQ), a lysosomal degradation inhibitor, and found that the protein level of APP was significantly increased in APP_K612T_ compared with APP_swe695_ cells (Figure 7, A and B). However, the autophagy-related proteins, including P62 and LC3 (Supplemental Figure 8, A–C), remained unchanged. To further determine whether APP_K612T_ is involved in the process of autophagic-lysosomal degradation, we treated cells with BafA1, an inhibitor of autophagic-lysosomal degradation pathway, and found that there were no significant changes in APP, P62, and LC3 in APP_K612T_ compared with APP_swe695_ cells (Supplemental Figure 8, D–G). Notably, when we detected the degradation process of APP via the cytoplasmic membrane by biotinylated markers, we found that the degradation rate of APP in APP_K612T_ cells was significantly increased than that in APP_swe695_ cells at 120 minutes, while there seemed to be no significant change in APP_K612Q_ cells (Figure 7, C and D). Collectively, these findings suggest that APP-K612la promotes the endosomal-lysosomal degradation process of APP rather than the autophagic pathway.

Next, we wanted to further determine the mechanism by which APP_K612T_ promotes the endosomal-lysosomal degradation pathway of APP. A series of studies have shown that APP is internalized and sorted into early endosomes, where it is cleaved by BACE1 to produce Aβ (45, 46). Given that APP_K612T_ facilitates the aggregation of APP in the early endosomes, we isolated the endosomal proteins to examine the cleavage product of APP by BACE1. Surprisingly, we found that CTF-β and sAPP-β were significantly decreased in APP_K612T_ cells, while they remained unchanged in APP_K612Q_ cells compared with APP_swe695_ cells (Figure 7, E and F). PS1 expression levels in endosomal proteins also showed no significant changes (Figure 7G). Co-IP assays revealed that APP_K612T_ significantly inhibited the interaction between APP and BACE1 (Figure 7H and Supplemental Figure 9, A and B), potentially protecting APP from BACE1 cleavage and the generation of CTF-β. It has been reported that APP trafficking for degradation is regulated by CD2AP (47). We, therefore, detected the protein expression of CD2AP in the early endosomes. The results showed that no significant change in the total protein level of CD2AP was observed in APP_K612T_ compared with APP_swe695_ cells (Figure 7I and Supplemental Figure 9, C and D). However, the level of CD2AP in the early endosomes was significantly increased in APP_K612T_ cells, while it was decreased in APP_K612Q_ cells compared with APP_swe695_ cells (Figure 7J). To further confirm the effect of CD2AP on APP cleavage, we overexpressed CD2AP and found that the amount of APP in the early endosomes was reduced to control (APP_swe695_) level in APP_K612T_ cells, while it was significantly increased in APP_K612Q_ cells compared with APP_swe695_ cells (Figure 7K). Further Co-IP results clearly demonstrated that APP_K612T_ promoted the interaction between APP and CD2AP, whereas APP_K612Q_ inhibited their interaction (Figure 7L). Immunofluorescence results also showed that APP_K612T_ increased the colocalization of CD2AP and EEA1 (Figure 7, M and N). Taken together, these results suggest that APP-K612la inhibited APP binding to BACE1 and subsequent cleavage in the early endosomes and, instead, promoted the protein interactions between APP and CD2AP, which may accelerate the endosomal-lysosomal degradation of APP.

### APP-Kla is susceptible to L-lactate modulation, which reduces Aβ pathology and cognitive impairment in AD model mice.

**Figure 8:**
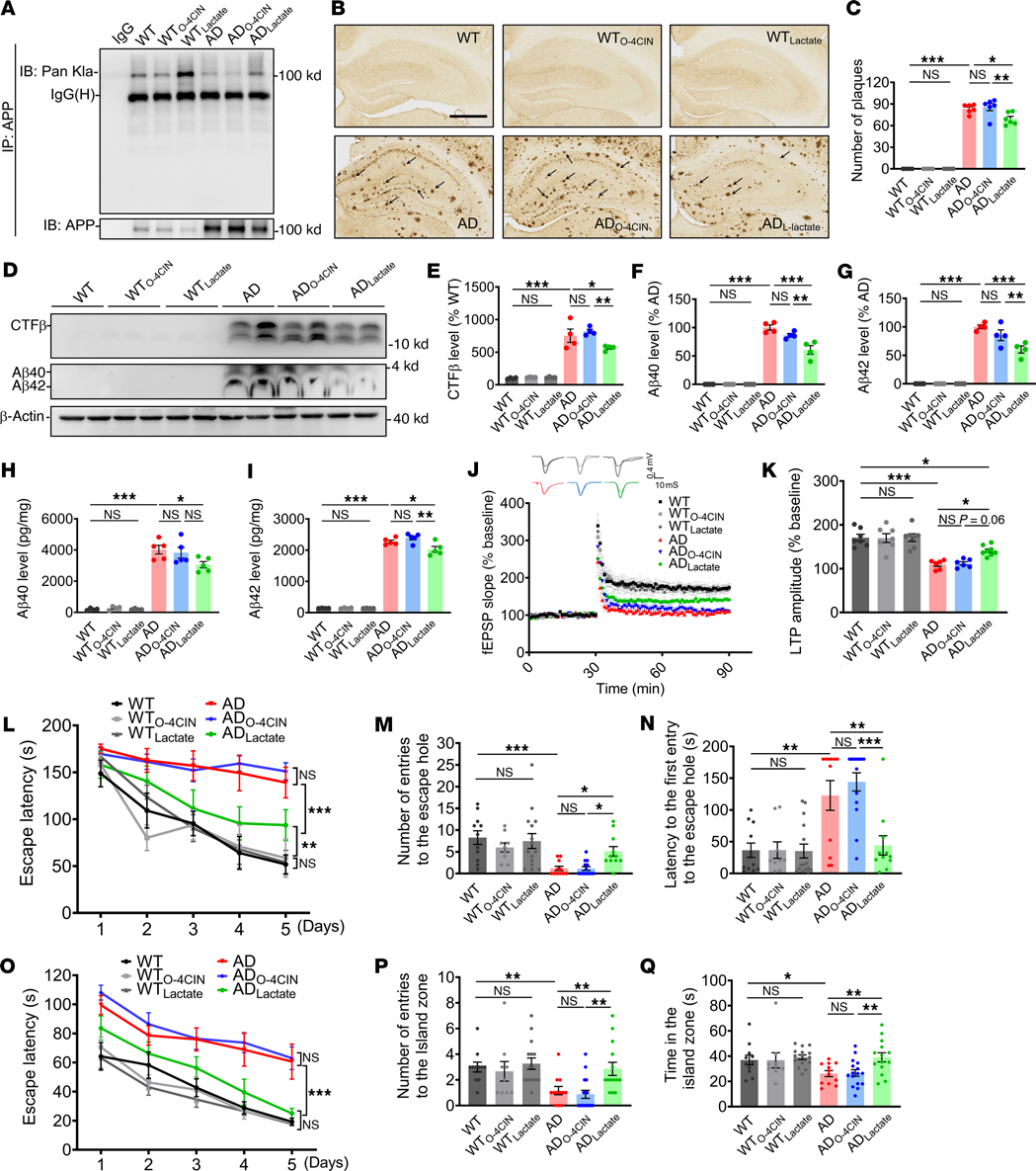
L-lactate enhancement of APP-Kla reduced Aβ pathology and cognitive impairment in AD model mice. (**A**) Hippocampus tissue lysates were immunoprecipitated using an APP antibody, followed by immunoblot analysis with a Pan-Kla antibody to detect APP-Kla expression in WT and APP23/PS45 mice treated with L-lactate or O-4CIN at the age of 6 months (*n* = 6 in each group). (**B** and **C**) Representative IHC staining images and quantitative statistics of hippocampal senile plaques in mice (scale bar: 500μm, *n* > 30 slices from 6 mice in each group). (**D**–**G**) The relative protein levels of CTF-β (**E**), Aβ40 (**F**), and Aβ42 (**G**) were assessed by Western blot (*n* = 4 in each group). (**H** and **I**) Generation of Aβ40 (**H**) and Aβ42 (**I**) as measured by ELISA (*n* = 4–5 in each group). (**J** and **K**) Hippocampal CA1 LTP recorded from mice brain slices (**J**) and the bar graphs of the average percentage changes in the fEPSP slope 55–60 min after TBS delivery (**K**) (*n* = 6–8 slices from 3–4 mice in each group). (**L**) The latency to the escape hole during spatial learning in the Barnes maze paradigm (*n* = 9–15 in each group). (**M** and **N**) The correct number of finding the escape hole (**M**) and latency to finding the escape hole (**N**) during memory retrieval in the Barnes maze test (*n* = 9–15 in each group). (**O**) The latency for finding the hidden island during spatial learning in the Morris water maze test (*n* = 9–15 in each group). (**P** and **Q**) The number of finding hidden platform quadrant zone during spatial learning (**P**) and time for entries to the island zone (**Q**) in the Morris water maze test (*n* = 9–15 in each group). Data were presented as mean ± SEM, *P < 0.05, **P < 0.01, and ***P < 0.001 by 2-way ANOVA, followed by Tukey’s multiple comparisons test (**C**, **E**–**G**, **H**, **I**, and **K**–**Q**).

To determine the pharmacological modulation of APP lactylation levels and whether it would be protective against AD, we treated 2-month-old APP23/PS45 AD model mice with L-lactate or a combined application of sodium oxalate and 4CIN (O-4CIN) (36) and tested Aβ pathology and spatial learning and memory in 6-month-old mice (Supplemental Figure 10A). The results showed that L-lactate treatment promoted the protein expression of APP-Kla in hippocampal tissues of AD-model mice (Figure 8A). Further IHC staining showed that L-lactate treatment reduced the number of senile plaques in the hippocampus of AD model mice, whereas O-4CIN treatment showed no significant changes (Figure 8, B and C). Meanwhile, we found that L-lactate treatment decreased the protein levels of CTF-β, Aβ40, Aβ42, and PS1 in AD mice (Figure 8, D–G, and Supplemental Figure 10, B and E). However, APP and BACE1 expression levels remained unchanged (Supplemental Figure 10, B–D). These results suggest that L-lactate treatment may also reduce APP cleavage by γ-secretase through lowering PS1 levels, leading to a reduction in Aβ production. Notably, O-4CIN had no effects on the expressions of AD-related pathological proteins including CTF-β, Aβ40, and Aβ42 (Figure 8, D–G). ELISA measurements showed significantly lower Aβ40 and Aβ42 levels in the hippocampus of L-lactate–treated mice compared with AD-model mice (Figure 8, H and I). Collectively, these data suggest that increased APP-Kla level is modulated by L-lactate and inhibits Aβ production and senile plaque formation in AD-model mice.

Next, we further investigated the effects of L-lactate treatment on hippocampal CA1 LTP and spatial learning and memory in AD model mice. The results showed that the hippocampal CA1 LTP was reduced in AD model mice compared with WT mice, but it increased in AD model mice treated with L-lactate (Figure 8, J and K). In the Barnes maze test (Supplemental Figure 11A), the spatial learning ability of AD-model mice was markedly impaired compared with WT mice, while it partially recovered, although not restored to the control level in the AD mouse model treated with L-lactate (Figure 8L). In the probe trial, L-lactate–treated mice increased the number of times they correctly found the escape hole and shortened the latency to find the escape hole (Figure 8, M and N), indicating better memory retrieval in AD-model mice treated with L-lactate. Similarly, the Morris water maze test also showed that spatial learning and memory retrieval were impaired in AD mice, while these impairments were partially ameliorated in L-lactate–treated AD-model mice (Figure 8, O–Q). In addition, we analyzed the swimming speed of mice in the Morris water maze test and found no significant differences in any of them (Supplemental Figure 11, B and C). In behavioral tests, including the open-field test and elevated-plus maze experiments, no significant differences in total distance traveled were observed among these groups. However, O-4CIN–treated AD mice exhibited anxiety- and depression-like behaviors, while L-lactate treatment had no significant effects compared with WT and AD mice (Supplemental Figure 11, D–G). In the elevated plus maze test, the number of entries and the time spent in the open arms were similar across groups (Supplemental Figure 11, H–J). Taken together, these results suggest that the level of APP lactylation modification is susceptible to L-lactate modulation, lowers Aβ generation and deposition, and rescues the deficits of spatial learning and memory in APP23/PS45-model mice.

## Discussion

APP PTMs play important roles in APP metabolism and Aβ deposition (48). Recently, histone lactylation has emerged as an epigenetic modification regulated by lactate content, linking intracellular metabolism and gene regulatory functions (26). Given that APP is a key gene in AD pathogenesis, understanding the role and mechanism of APP lactylation holds promise for providing a direction and target for AD treatment. In this study, we found that APP lactylation occurred at the K354 (APP-K354la), K363 (APP-K363la), and K612 (APP-K612la) sites. Subsequent experiments using lactyl-mimicking mutants revealed that APP-K612la, rather than APP-K354la and APP-K363la, significantly reduced the protein level of CTF-β. Notably, APP and its proteolytic enzymes, including ADAM10, BACE1, and PS1, did not exhibit any changes (Figure 2, K–Q), suggesting that APP-K612la may affect APP trafficking and metabolism but not its expression. Supporting this perspective, evidence suggests that lactylation modification of Vps34 promotes Vps34 binding to PI3KC3 complex I and II subunits, thereby enhancing Vps34 kinase activity and promoting autophagy and endosomal-lysosomal transport (19). Similarly, our findings showed that APP-K612la reduced the amount of APP on the plasma membrane, causing it to aggregate in the endosomes and lysosomes (Figure 6), indicating an increase in APP trafficking from the plasma membrane to the endosomes. Moreover, we reported that APP-K612la accelerated the endosomal-lysosomal degradation pathway of APP, resulting in decreased CTF-β production and Aβ deposition. However, it is noteworthy that APP-K612la, while reducing APP amyloidogenic processing, also appears to inhibit nonamyloidogenic processing, as evidenced by a significant decrease in sAPP-α and CTF-α (Supplemental Figure 2, D and E). This reduction may be attributed to the fact that K612-L613 functions as an APP α-secretase cleavage site. Previous studies have demonstrated that altering this site from lysine to asparagine (APP_K16N_) could lead to diminished α-secretase cleavage, potentially impairing neuroprotection and neurogenesis in the brain (49). Moreover, succinylation of APP at the K612 site might promote Aβ production by inhibiting α-secretase cleavage (22). In this study, when we mutated its lysine site to the unlactylated form of glutamine (APP_K612Q_), it appeared to have no significant effect on the pathological progression of AD. Surprisingly, when we mutated it to the lactylation form of threonine (APP_K612T_), it markedly reduced Aβ pathology and improved synaptic and cognitive functions in vivo, despite the reduction in α-cleavage. We speculate that APP_K612T_ may facilitate subsequent APP endocytosis by inhibiting α-cleavage at the plasma membrane, where most α-secretase cleavage occurs. If APPK612la translocates more readily from the plasma membrane than APP_swe_, this may reduce its interaction with ADAM10. Furthermore, endocytosed lactylated APP may inhibit BACE1 binding and promote APP degradation via the endosomal-lysosomal pathway. These findings suggest that APP-K612la offers potential therapeutic benefits for AD, warranting further research into its specific molecular mechanisms.

CD2-associated protein (CD2AP), an adaptor protein regulating membrane trafficking, plays an important role in signaling transduction and cytoskeletal regulation. Growing evidence suggests that CD2AP loss of function contributes to increased Aβ generation, Tau-induced neurotoxicity, synaptic dysfunction, and abnormal synapse architecture (50). CD2AP loss of function has been reported to raise Aβ generation by increasing the convergence of APP and BACE1 in the early endosomes (47), whereas its overexpression stimulates APP degradation by facilitating the transfer of APP from the early to late endosomes (51). In the present study, we found that APP-K612la promoted CD2AP protein localization in the early endosomes and enhanced the interaction between APP and CD2AP, thereby accelerating the degradation process of APP via the endosomal-lysosomal system. Surprisingly, although APP-K612la appeared to promote APP endocytosis from the plasma membrane into the early endosomes, it inhibited the binding of APP to BACE1, leading to the aggregation of APP in the early endosomes without being cleaved by BACE1. Interestingly, a previous study provided clear evidence that APP translocated from the cell surface, but depletion of Hrs and Tsg101, acting early in the multivesicular body pathway, arrested endocytosed APP in the early endosomes without cleavage by BACE1, thereby reducing the production of Aβ peptides (52). However, further research is needed in the future to investigate how APP-K612la inhibits the binding of APP to BACE1 and promotes the CD2AP-mediated degradation process of APP through the endosomal-lysosomal system.

APP lactylation was reduced in AD, and a lactyl-mimicking mutant, APP_K612T_, inhibited Aβ generation and slowed down memory decline in vivo, suggesting a neuroprotective role of APP-K612la in AD. In the APP23/PS45 double-transgenic mouse model of AD, the administration of L-lactate increased the level of APP lactylation modification, resulting in lower Aβ generation and deposition, as well as alleviated deficits in hippocampal CA1 LTP and cognition (Figure 8). It is crucial to note that, while L-lactate promoted APP lactylation and ameliorated cognitive deficits in AD model mice, its effects were broad spectrum and double sided (36). Lactate serves as an energy source for neurons and a signaling molecule regulating neuronal function, including excitability, plasticity, and memory consolidation (53–58). On the other hand, L-lactate may promote the lactylation of H4K12, potentially exacerbating glucose metabolism disorders and microglia dysfunction in AD (29). This dual role may explain why L-lactate treatment did not fully rescue synaptic and cognitive dysfunctions in AD model mice in the present study (Figure 8, K and L). Nevertheless, considering the enhancing effect of L-lactate on APP lactylation, a major contributor to Aβ production, we believe that L-lactate treatment has a positive impact on alleviating AD pathology and cognitive deficits. Of course, ongoing exploration of small molecule compounds targeting APPK612la to promote APP lactylation at specific sites is essential, providing the possibility of achieving precise treatment for AD in the future. Notably, protein lactylation is often parallel to lactate levels (26). Clinical studies have reported elevated cerebrospinal fluid lactate levels in AD, although some reports suggest no change or a decrease (57, 59–61). In the present study, we observed a reduction in APP lactylation in AD, which is different from a recent report that histone H4K12la was significantly increased in microglia of 5×FAD mice (29). These discrepancies may arise from differences in cell types, as increased L-lactate in AD is observed primarily in nonneuronal cells, likely due to defects in the astrocyte-neuron lactate shuttle system (57, 62, 63). Lactate transport across membranes is mediated by monocarboxylate transporters (MCTs), members of the solute carrier 16A (SLC16A) family. It has been reported that reduced MCT1, MCT2, and MCT4 expression has been associated with neuronal energy deficits, resulting from impaired lactate transport in APP/PS1 AD–model mice (57). The observed reduction in APP lactylation may stem from diminished lactate influx in neurons of patients with AD and mouse models, given that APP695 is predominantly expressed in neurons (64, 65).

**Figure 9:**
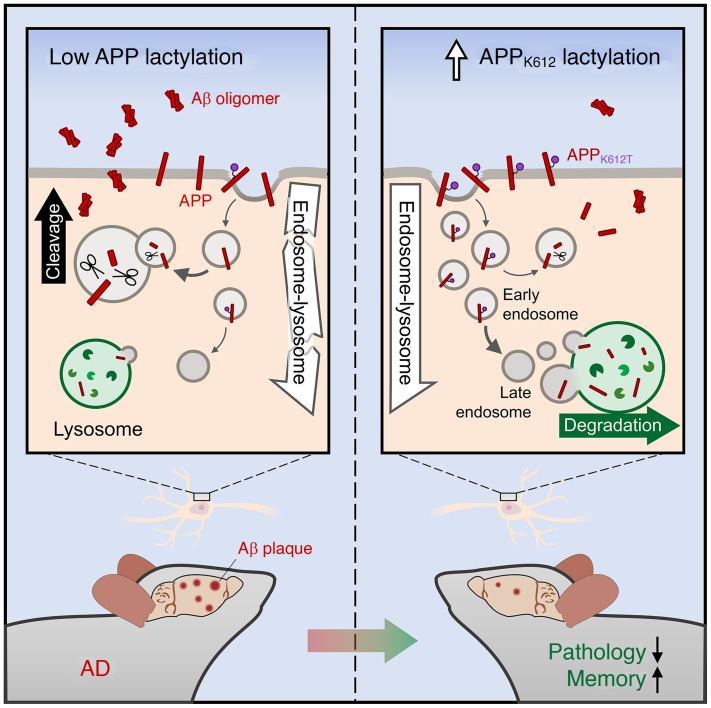
Schematic illustrates that lactylation of APP_K612_ ameliorates amyloid pathology and memory decline in AD. APP lactylation expression was reduced in AD, and upregulation of APP by the APP_K612T_ mimetic variant ameliorates amyloid pathology and memory decline by promoting APP endosomal-lysosomal degradation.

In conclusion, our results suggest that the regulation of APP lactylation plays an essential role in AD pathogenesis. By using a lactyl-mimicking mutant (APP_K612T_), we found that APP-K612la may facilitate APP endocytosis from the cytoplasmic membrane to the early endosomes. Lactylated APP in the endosomes inhibits its binding to BACE1 and subsequent cleavage but, conversely, promotes the protein interaction between APP and CD2AP, which may accelerate the endosomal-lysosomal degradation system and lead to reduced Aβ deposition and improved memory in AD model mice (Figure 9). Thus, these findings provide what we believe to be new insights into the role of APP lactylation modification in the pathogenesis of AD and establish a scientific basis for the potential development of APP lactylation-specific agonists as therapeutics for treating learning and memory deficits associated with AD and aged populations.

## Methods

### Sex as a biological variable.

Our study examined human samples from males and females, as well as male and female mice, and similar findings are reported for both sexes.

### Human samples.

Human brain tissue samples were obtained from the Chinese Human Brain Bank of Zhejiang University and the detailed information was shown in Supplemental Table 1. Briefly, proteins were extracted using Western and IP lysates (containing cOmplete Tablets EDTA-free) and lysed on ice for 20 minutes. Subsequently, the proteins were centrifuged (12,000*g*, 4 °C, 15 minutes) and the supernatant was collected for Western or Co-IP protein assays.

### Animals.

APP23/PS45 double–transgenic AD model mice were carrying the mouse/human chimera APP23 (Swedish APP751) as well as the human mutant PS45 (G384A mutant presenilin 1) gene (provided by Cyagen). Six-month-old male and female halves of APP23/PS45 double-transgenic mice, PS45 single-transgenic mice, and WT cobred mice (weight, approximately 25 g) were housed at the Experimental Animal Center of the Children’s Hospital of Chongqing Medical University (23 ± 1°C, 12 hour light-dark cycle, lights on a 7 a.m.–7 p.m.) and allowed free access to mouse-specific food and water. Genotype detection was confirmed by PCR using tissue DNA. Before the experiments, the animals were allowed to acclimatize in the animal facility for 1 week before performing experimental manipulations.

To promote the expression level of APP-Kla in AD, Sodium L-lactate (1 g/kg/day, Sigma-Aldrich, no. L7022) was used to promote Kla expression, and Sodium oxamate (1 g/kg/day, Yuan Ye, no. S30701) in combination with 4CIN (150 mg/kg/day, Sigma-Aldrich, no. C2020) was used to inhibit Kla expression, according to a previous report (36). Two-month-old WT and AD mice were administered intraperitoneally for 90 days and subsequently tested for behavioral and pathological analysis.

To express APP_swe695_, APP_K612Q_, and APP_K612T_ mutant locus genes in vivo, adeno-associated viruses were constructed by OBiO Technology (Serotype, AAV2/8. Vector, H13559, pAAV-CMV-3×FLAG-P2A-mNeonGreen-tWPA). A brain stereotaxic instrument (STOELTING, USA) was used for bilateral hippocampal CA1 localization injections, with fontanelle as the origin, 2.5 mm posterior to fontanelle, and 2.0 mm paracentral to the left and right, and a 10 μL microinjector was used to slowly insert the needle at a depth of 2.5 mm. The microinjector pump slowly injected 1 μL of adeno-associated virus (titer: 3×10^12^ TU/ml) at a rate of 0.2 μL/min. To allow sufficient spread of the virus, the needle was left in place for 5 minutes after injection and then slowly withdrawn.

### Antibody.

C20 synthesized from GL Biochem (Shanghai) Ltd. was used to detect APP and CTFs (synthetic peptide corresponding to a sequence within amino acids 751KMQQNGYENPTYKFFEQMQN770 of human APP). The detailed information was shown in Supplemental Table 2.

### Plasmids.

The APP lactylate-mimic plasmids were constructed by homologous recombination with mutation of each locus from lysine to threonine. In contrast, mutation to glutamine constructed the APP unlactylated mimic plasmids (39, 40). We used APP_swe695_ as the full-length isoform for lactylation site mutation in plasmid design numbering. APP plasmids were amplified from human cDNA and inserted into pCDNA4.1. DNA fragments containing the homologous end sequence were amplified from the APP plasmid using the primers. The detailed information was shown in Supplemental Table 3. Specific plasmid design patterns and lysine mutation sequencing maps were detailed in Figure 2, B–D. The plasmid of CD2AP (tagged HA, mCherry) was amplified from human cDNA and inserted into pcDNA3.1.

### Cell culture and transfection.

HEK293 cells were purchased from the American Type Culture Collection (ATCC, USA) and cultured in an incubator at 37 °C with 5% CO_2_. Basal medium containing 10 % total FBS, 100 U/mL penicillin, and 0.1 mg/mL streptomycin DMEM (Gibco, USA) was used to culture the cells. Plasmids, sgRNA, or empty vectors were transfected into cells using Lipofectamine 3000 (Invitrogen, no. L300008) for coimmunoblotting. The medium was completely replaced after 4–6 hours of transfection and the cells were further incubated for 24 hours.

To construct APP_KO_ stable cell lines, CRISPR/Cas9 system technology was introduced. Briefly, a sgRNA targeting intron 5 of the APP gene was designed using the CRISPR design tool from the Ran and colleagues (66). HEK293 cells were transfected with oligonucleotide cloned plasmids with sgRNA sequences (oligo 1, TTCTCGTTCCTGACAAGTGC; oligo 2, GCACTTGTCAGGAACGAGAA) to generate APP_KO_ cell lines. The sgRNA/Cas9-positive cells were screened using puromycin (0.5 mg/mL) 24 hours after transfection. Single-cell seeding into 96-well plates was then performed to screen for monoclonal APP_KO_ cell lines. Single cells were amplified and DNA was extracted for PCR and sequencing assays (Figure 1I) (Forward primer, 5′-GGTCTTGATTGGGTTGCTTAGGCA-3′; Reverse primer, 5′-GGCATGGCACAGACAACTTATATTTTTAAGT-3′). The knock-out level of APP protein was determined by Western blotting (Figure 1J).

### LC-MS/MS analysis.

LC-MS/MS analysis was conducted with the assistance of Jingjie PTM Biolabs (Hangzhou, China). The tryptic peptides were dissolved in 0.1% formic acid and directly loaded onto a homemade reversed-phase analytical column. On the EASY-nLC 1000 UPLC system, the peptides were subjected to an NSI source followed by tandem mass spectrometry (MS/MS) in Q ExactiveTM Plus (Thermo Fisher Scientific) coupled online to the UPLC. Peptides were then selected for MS/MS. The resulting MS/MS data were processed using the Maxquant search engine (v.1.5.2.8). Tandem mass spectra were searched against human uniprot database concatenated with reverse decoy database.

### RNA-seq analysis.

RNA-seq analysis was conducted with the assistance of Bioprofile (Shanghai, China). Total RNA was isolated from mouse hippocampal tissues using Trizol reagent (Invitrogen Life Technologies), and then its concentration, quality, and integrity were determined using a NanoDrop spectrophotometer (Thermo Fisher Scientific). RNA-seq and RNA-seq analyses were performed by BioArchive Technologies (Shanghai, China). Briefly, after RNA extraction, purification, and library construction, the libraries were subjected to paired-end (PE) sequencing using Next-Generation Sequencing (NGS) based on the Illumina sequencing platform. The raw downstream data (Raw Data) were first filtered, and the filtered high-quality sequences (Clean Data) were aligned to the reference genome of the species. Based on the comparison results, the expression amount of each gene was calculated and the samples were further subjected to expression difference analysis, enrichment analysis, and cluster analysis.

### Western blotting.

Cells and brain tissue were lysed using RIPA (containing cOmplete Tablets EDTA-free) buffer to extract proteins. Protein concentrations were measured and adjusted for aliquots using the BCA Protein Assay Kit (Pierce, no. 23227). Samples were subsequently separated by SDS-PAGE and transferred to PVDF membranes (Sigma-Aldrich). After blocking with 5% skim milk in a TBST buffer for 1 hour, the membranes were incubated with antibodies overnight at 4°C with slow shaking. The membranes were then incubated with the corresponding horseradish peroxidase-conjugated antibodies for 1 hour at room temperature and quantitative densitometric analysis was performed with a Bio-Rad Imager using ECL Western blotting substrate (Pierce) or an Oddessy imaging system, and the resulting fold changes were indicated by normalizing.

### Aβ ELISA.

Mouse hippocampal homogenates were collected, and a protease inhibitor (Roche, cOmplete Tablets EDTA-free) was added to prevent Aβ degradation. Aβ40 and Aβ42 levels were determined using ELISA Kits (R&D, no, DAB140B and DAB142), and samples were read at 450 nm using a microplate reader (Bio Tek Synergy H1).

### Immunostaining and confocal microscopy.

For immunofluorescence detection, coronal cryosections (30 μm) of mouse brain were frozen and stored in cryoprotective storage solution (30% sucrose, 1% polyvinyl-pyrrolidone, 5 mM Na_2_HPO_4_, 20 mM NaH_2_PO_4_, and 30% ethylene glycol) at –20°C until use. Cells were transfected and inoculated in confocal culture dishes. Cell nuclei were counterstained with DAPI (4,6-diamidino-2-phenylindole) and plates were blocked with an antifluorescence quencher (Solarbio, no. S2100). Cells were imaged and analyzed with a Nikon A1R scanning confocal microscope. Statistical analysis of Manders’ overlap coefficients (67) was performed using Nis-Elements AR analysis.

### Co-IP.

Protein A/Agarose beads (Sigma-Aldrich, no. P2545, mixed with PBS to 50% concentration) were added to the protein and placed on a shaker at 4°C for 10 minutes to remove nonspecific heteroproteins and reduce the background. The concentration of the supernatant protein was determined by BCA and adjusted for consistency, and then the antibody (in the ratio as directed) was added to the supernatant. The antigen-antibody mixture was shaken slowly at 4 °C overnight. Then add the corresponding amount of agarose beads to capture the antigen-antibody complexes and shake the antigen-antibody complexes slowly at 4 °C overnight or at room temperature for 2 hours. After centrifugation, the agarose beads-antigen-antibody complexes were collected and washed 3 times with prechilled PBS. A total of 20–40 μl of 2× sample buffer was boiled at 95 °C for 5 minutes to free the antigen, antibody, and beads. The samples were then centrifuged at 14,000*g* for 30 seconds and the supernatant was used for Western blotting.

### Trafficking assays.

Surface biotinylation assays were used according to previous reports (47, 68, 69). Briefly, after transfection, cells were placed on ice and rinsed with prechilled PBS. Cells were then incubated in PBS containing 1 mg/mL Sulfo-NHS-LC-Biotin (Pierce, no. PG82075) for 30 minutes at 4°C and rinsed twice with PBS. To determine the total protein concentration, 10% of the cell lysate was removed for immunoblotting. To isolate biotinylated proteins, 90% of the cell lysates were immunoprecipitated with 50 μL of NeutrAvidin agarose (Pierce, no. 29200) overnight at 4°C, washed and immunoblotted, and the data were quantified by comparing the ratio of biotinylated proteins to total proteins.

For APP endocytosis, cells were incubated with 1 mg/mL Sulfo-NHS-LC-Biotin at 4 °C for 30 minutes, then rinsed with prechilled PBS and lysates were immediately prepared, or biotinylated proteins were chased for 10 minutes and 30 minutes at 37°C and cells were immediately placed on ice and incubated in cold stripping buffer (50 mM glutathione, 75 mM NaCl, 75 mM NaOH, 10% FBS, pH 8.5–9.0) to strip the surface biotin to detect endocytosis of APP. Biotinylated proteins were immunoprecipitated with NeutrAvidin agarose beads. Isolated proteins were rinsed 3 times in buffer and added to 20 μL of sample buffer and boiled for 5 minutes for immunoblotting. See Figure 6C for an abbreviated schematic.

For APP degradation, cells were incubated with 1 mg/mL Sulfo-NHS-LC-Biotin at 4°C for 30 minutes, then rinsed with prechilled PBS, and lysates were prepared immediately, or biotinylated proteins were chased for a further 60 minutes and 120 minutes at 37°C, then proteins were immunoprecipitated with NeutrAvidin agarose beads, washed, and immunoblotted. See Figure 7C for an abbreviated schematic.

### Organelle protein isolation.

For plasma membrane protein isolation, the Minute Plasma Membrane Protein Isolation and Cell Fractionation Kit (Invent, no. SM-005) was used, and the steps in the instructions were strictly followed. The results of plasma membrane protein isolation purity verification are shown in Supplemental Figure 7A. For endosomal protein extraction, the Minute Plasma Membrane Protein Isolation and Cell Fractionation Kit (Invent, no. ED-028) was used, and the steps in the instructions were strictly followed. The results of endosomal protein extraction purity verification are shown in Supplemental Figure 7B. For lysosome protein isolation, the Minute Lysosome Isolation Kit (Invent, no. LY-034) was used, and the steps in the instructions were strictly followed. The results of lysosome protein isolation purity verification are shown in Supplemental Figure 7C.

Immunoblotting assays were performed using Minute Denaturing Protein Solubilization Reagent (Invent, no. WA-009) to dilute the precipitates, and immunoprecipitation assays were performed using Minute Nondenatured Protein Solubilization Reagent (Invent, no. WA-010).

### IHC staining.

Following our previously reported method (70). Briefly, mouse brain tissue was postfixed in freshly precooled 4% paraformaldehyde (PBS, 0.1 M, pH 7.4) for 48 hours, followed by gradient dehydration with 20%–30% sucrose. Brain tissue was processed for embedding and frozen sectioning using OCT. Sections were 30 μm thick coronal sections. To eliminate residual peroxidase, the sections were incubated with 3 % H_2_O_2_ for half an hour. Sections were then blocked with 10% BSA and incubated with mouse monoclonal 4G8 antibody at 4°C overnight. Plaques were stained with ABC and DAB methods and counted at ×40 magnification.

### Barnes maze test.

The setup for the Barnes maze test consisted of a white circular platform (0.75 m diameter) with 18 holes (5 cm diameter) around the edge, with 1 hole arbitrarily chosen as the escape hole. Following our previously reported method (71). The Barnes maze test consists of 3 phases: the adaptive phase, the acquisition training phase, and the learning memory test. After each mouse’s use, the maze platform and escape hole were thoroughly cleaned with 75 % alcohol for the next mouse’s use. Twenty-four hours after the last learning trial, the escape hole was removed and a 3 minute probe trial was performed. Any-maze tracking system (Stoelting Co.) was used to record escape latency.

### Morris water maze test.

The Morris water maze test was introduced to detect hippocampal-based spatial memory in mice. As previously described (72), the maze consisted of a circular stainless-steel pool with a diameter of 150 cm filled with a nontoxic pool filler and maintained at a temperature of 24 ± 1°C. For the first 24 hours of maze space learning (day 0), mice underwent 120 seconds of free swimming to acclimatize to the environment. Then, mice were trained to find the hidden escape platform (13 cm in diameter) and recorded the escape latency. Four trials were performed each day for 5 consecutive days (days 1–5). Twenty-four hours after the last training trial (day 6), the hidden platform was removed and a learning memory retrieval test was performed. Any-maze tracking system (Stoelting Co.) was used to record escape latency.

### Electrophysiology in vitro.

For LTP recordings, the CA1 layer radiatum of the hippocampus was stimulated by stimulating the Schaffer collateral/commissural pathway in response to recording field excitatory postsynaptic potentials (fEPSPs). Following our previously reported method (72). Briefly, mice were deeply anesthetized, the brains were rapidly removed after perfusion with artificial cerebrospinal fluid (ACSF) and oxygenated with 95% O_2_ and 5% CO_2_, then the brains were coronal cut (400 μm) using a vibrating microtome (VT1200S, Leica Microsystems) and transferred to a submersion-type incubation chamber and recovered at 35 °C for 2 hours. A bipolar stimulation electrode was placed on Schaffer collaterals of CA3 pyramidal neurons in the dorsal hippocampus and a recording pipette filled with ASCF was placed on the ipsilateral striatum in the CA1 region of the hippocampus. The frequency of evoked test EPSC was 0.05 Hz, and the stimulus intensity was adjusted to 50 % of the maximum response. After a 30 minute stable baseline, theta-burst stimulation (TBS) was given to induce LTP. Data acquisition was performed with PatchMaster v2.73 software. (HEKA Electronic, Germany)

### Statistics.

All data are expressed as the mean ± SEM. ANOVA or 2-tailed Student’s *t* tests were used to analyze the data as appropriate. The statistical analyses were performed using Prism 8.0 (GraphPad Software). Analysis items with *P* < 0.05 were considered statistically significant.

### Study approval.

The human sample study was evaluated and approved by the Ethics Committee of Zhejiang University (Research Project Ethics Approval No. 2018-009). All animal experiments were performed according to the regulations of the Chongqing Municipal Science and Technology Commission and were authorized by the Chongqing Medical University Animal Care Committee (CHCMU-IACUC20220323012), and every effort was made to reduce animal suffering and the number of animals used.

### Data availability.

Raw data of sequencing data were deposited in the Sequence Read Archive (SRA) under accession number PRJNA1175063. The values for all data points in the graphs are reported in the Supporting Data Values file.

## Author contributions

ZD, YD, and QT conceived the study and wrote the manuscript. QT, JL, BW, WH, QX, JW, LY, NT, MC, YF, BX, and YT performed behavioral and biochemical experiments. YP and JL performed electrophysiological recordings. LX and XS performed immunofluorescence assay. WS and XT provided important discussions and suggestions. ZD contributed reagents. All authors have read and approved the final manuscript.

## Supplementary Material

Supplemental data

Unedited blot and gel images

Supporting data values

## Version 1 01/02/2025

Electronic publication
