# Supplementary material for: APP lysine 612 lactylation ameliorates amyloid pathology and memory decline in Alzheimer’s disease
Source: J Clin Invest. 2025 Jan 2;135(1):e184656. doi: 10.1172/JCI184656 (PMC11684803; doi:10.1172/JCI184656)
Supplement: Supplemental data [file jci-135-184656-s082.pdf]

Supplementary material

Supplemental Table 1. Information for patients with AD and normal controls

Case	Sex	Age (years)	Braak staging	Postmortem interval (min)
AD-1	M	85	IV	930
AD-2	M	87	V	389
AD-3	F	84	V	140
AD-4	F	86	IV	1080
AD-5	M	83	IV	270
AD-6	M	73	IV	570
CTR-1	M	98	-	220
CTR-2	M	87	-	300
CTR-3	F	98	-	277
CTR-4	M	81	-	675
CTR-5	M	52	-	1890
CTR-6	F	60	-	135

Note: AD = Alzheimer's disease; CTR = normal control.

Supplemental Table 2. Information for antibodies

Antibodies	SOURCE	IDENTIFIER
Rabbit anti-C20	GL Biochem (Shanghai) Ltd.	
Mouse monoclonal anti-β Amyloid	Santa Cruz	Cat# sc-28365
Mouse monoclonal anti-APP	Invitrogen	Cat# 14-9749-82
Rabbit monoclonal anti-APP	ABclonal	Cat# A17911
Rabbit polyclonal anti-Pan Kla	PTM BIO	Cat# 1401RM
Mouse monoclonal anti-6E10	BioLegend	Cat# SIG-39320
Mouse monoclonal anti-sAPPα	IBL	Cat# 11088
Rabbit Polyclonal anti-sAPPβ	Biolegend	Cat# SIG-39138
Mouse monoclonal anti-APP A4 (N)	EMD Millipore	Cat# MAB348
Rabbit monoclonal anti-BACE1	Abcam	Cat# ab108394
Rabbit monoclonal anti-PSEN1	Abcam	Cat# ab76083
Rabbit monoclonal anti-ADAM10	Abcam	Cat# ab124695
Mouse monoclonal anti-β-actin	Sigma	Cat# A5411
Mouse monoclonal anti-GAPDH	arigobio	Cat# ARG10112
Mouse monoclonal anti-Na K-ATPase	Abcam	Cat# ab7671
Mouse monoclonal anti-EEA1	CST	Cat# 48453
Mouse monoclonal anti-RAB7	Santa Cruz	Cat# sc-376362
Rabbit monoclonal anti-LAMP1	CST	Cat# D2D11
Mouse monoclonal anti-CD2AP	Santa Cruz	Cat# sc-25272
Mouse monoclonal anti-Flag	CST	Cat# 8146S
Rabbit monoclonal anti-Flag	CST	Cat# 14793S
Rabbit polyclonal anti-P62	Proteintech	Cat# 18420-1-AP

Rabbit polyclonal anti-LC3B	CST	Cat# 2775
Mouse monoclonal anti-GFAP	Bioss	Cat# bsm-33065M
Rat monoclonal anti-CD11B	Invitrogen	Cat# 14-0112-82
Mouse monoclonal anti-NEUN	Abcam	Cat# ab104224
Anti-Rabbit IgG (Goat) HRP	Perkin-Elmer	Cat# NEF812001EA
Anti-Mouse IgG (Goat) HRP	Perkin-Elmer	Cat# NEF822001EA
Anti-Mouse IgG (Alexa Fluor™ 568)	Invitrogen	Cat# A10037
Anti-Mouse IgG (Alexa Fluor™ 488)	CST	Cat# 4408
Anti-Rabbit IgG (Alexa Fluor™ 647)	Abcam	Cat# ab150075
Anti-Rabbit IgG (Alexa Fluor™ 555)	CST	Cat# 4413
Anti-Rat IgG (Alexa Fluor™ 555)	Abcam	Cat# ab150154

Supplemental Table 3. Information for APP related plasmid primers

Primers	Primer sequence
APP_{WT} F	ccaagctggctagttaagcttATGCTGCCCGGTTTGGCA
APP_{WT} R	gaagggccctctagactcgagCTAGTTCTGCATCTGCTCAAAGAACT
APP_{swe695} F	tcTGGATGCAGAATTCCGACATGACTCAGGATA
APP_{swe695} R	CGGAATTCTGCATCCAgaTTCACTTCAGAGATCTCCTCCGTC
APP_{K363Q} F	TCCAGGAGcAAGTGGAATCTTTGGAACAGGAAG
APP_{K363Q} R	TTCCACTTgCTCCTGGAAATGCTGGATAACTG
APP_{K363T} F	CCAGGAGAcAGTGGAATCTTTGGAACAGGAAGC
APP_{K363T} R	ATTCCACTgTCTCCTGGAAATGCTGGATAACTG
APP_{K354Q} F	CTGATAAGcAGGCAGTTATCCAGCATTTCCAG
APP_{K354Q} R	AACTGCCTgCTTATCAGCTTTAGGCAAGTTCTTTG

APP_{K354T} F	CTGATAAGAcGGCAGTTATCCAGCATTTCCAG
APP_{K354T} R	AACTGCCgTCTTATCAGCTTTAGGCAAGTTCTTT
APP_{K612Q} F	cAATTGGTGTTCTTTGCAGAAGATGTGGGTTC
APP_{K612Q} R	GCAAAGAACACCAATTgTTGATGATGAACTTCATATCCTGAGTC
APP_{K612T} F	AcATTGGTGTTCTTTGCAGAAGATGTGGGTTC
APP_{K612T} R	GCAAAGAACACCAATgTTTGATGATGAACTTCATATCCTGAGT

Supplementary methods

Open-field test

The mouse open-field experiment was used to test the voluntary locomotion of mice. The absent field device was a square box (40 cm × 40 cm × 60 cm) made of Plexiglas, which was equally divided into nine squares, with one square area at the top corner defined as the corner, three square areas along the edge defined as the peripheral area, and the square area in the middle defined as the center area. Before the test, the mice were placed into the experimental room to acclimatize the mice to the environment in advance so that the stress would not affect the behavioral test. During the test, one mouse was placed in the center area and allowed to explore freely for 10 minutes. The activities of the experimental mice were monitored and recorded and the data were analyzed using Any-maze tracking system.

Elevated plus maze

The elevated plus maze (EPM) experiment was used to detect anxiety and exploratory behavior in mice. The elevated plus maze apparatus is made of white organic glass

and consists of two open arms (50 cm × 5 cm) and two closed arms (50 cm × 5 cm), the development arm with a 0.5cm high ledge to prevent mice from falling and the closed arm with a 40cm high wall. The two arms were connected by a central square (5 cm × 5 cm) and the device was fixed to a support table 40 cm above the floor. Briefly, a mouse was placed in the center square of the EPM and allowed to explore freely for 5 min. The activity of the experimental mice was monitored and recorded and the data were analyzed by the Any-maze software. The device was cleaned with 75% ethanol to eliminate olfactory interference with the next mouse.

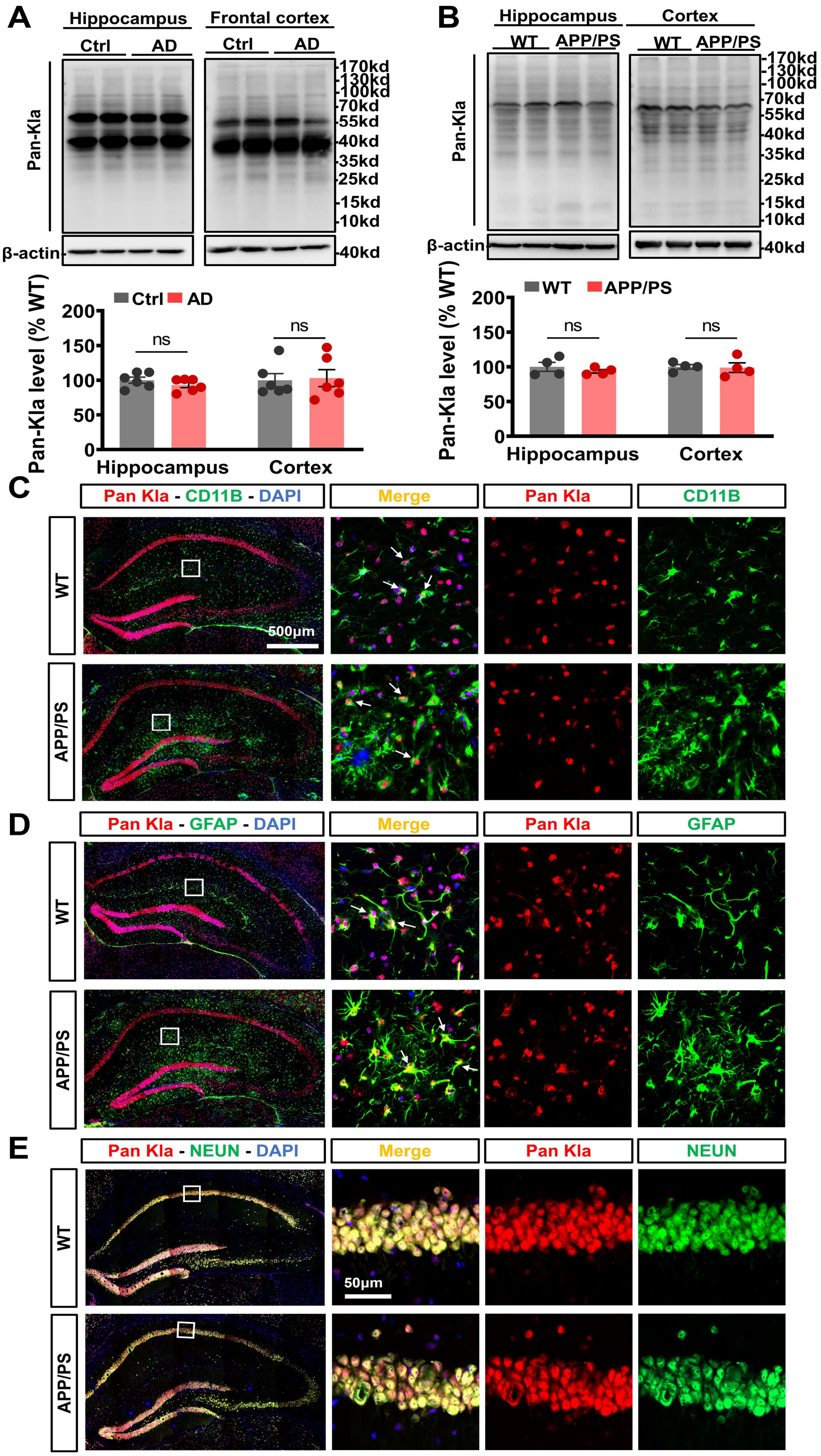
A
Hippocampus
Frontal cortex
Ctrl
AD
Pan-Kla
170kd
130kd
100kd
70kd
55kd
40kd
35kd
25kd
15kd
10kd
β-actin
40kd
Pan-Kla level (% WT)
Ctrl
AD
ns
Hippocampus
Cortex
B
Hippocampus
Cortex
WT
APP/PS
Pan-Kla
β-actin
WT
APP/PS
C
Pan Kla - CD11B - DAPI
Merge
Pan Kla
CD11B
WT
APP/PS
500μm
D
Pan Kla - GFAP - DAPI
GFAP
E
Pan Kla - NEUN - DAPI
NEUN
50μm

Supplemental Figure 1. Pan-Kla protein expression in AD patients and mice is not significantly altered but is widely distributed in hippocampal neuronal cells in AD mice.

(**A**) The protein levels of Pan-Kla were assessed by Western blot in the hippocampus and frontal cortex of AD patients and age-matched control subjects (n = 6 in each group). (**B**) The protein levels of Pan-Kla were assessed by Western blot in the hippocampus and cortex of WT and APP23/PS45 transgenic AD model mice at the age of 6 months (n = 4 in each group). (**C-E**) Representative confocal fluorescence images of Pan-Kla co-stained with CD11B, GFAP, and NEUN in 6-month-old WT and APP23/PS45 transgenic AD model mice. (n=4 in each group). Data were presented as mean ± SEM, ns, not significant by two-tailed unpaired Student's t-test (**A** and **B**).

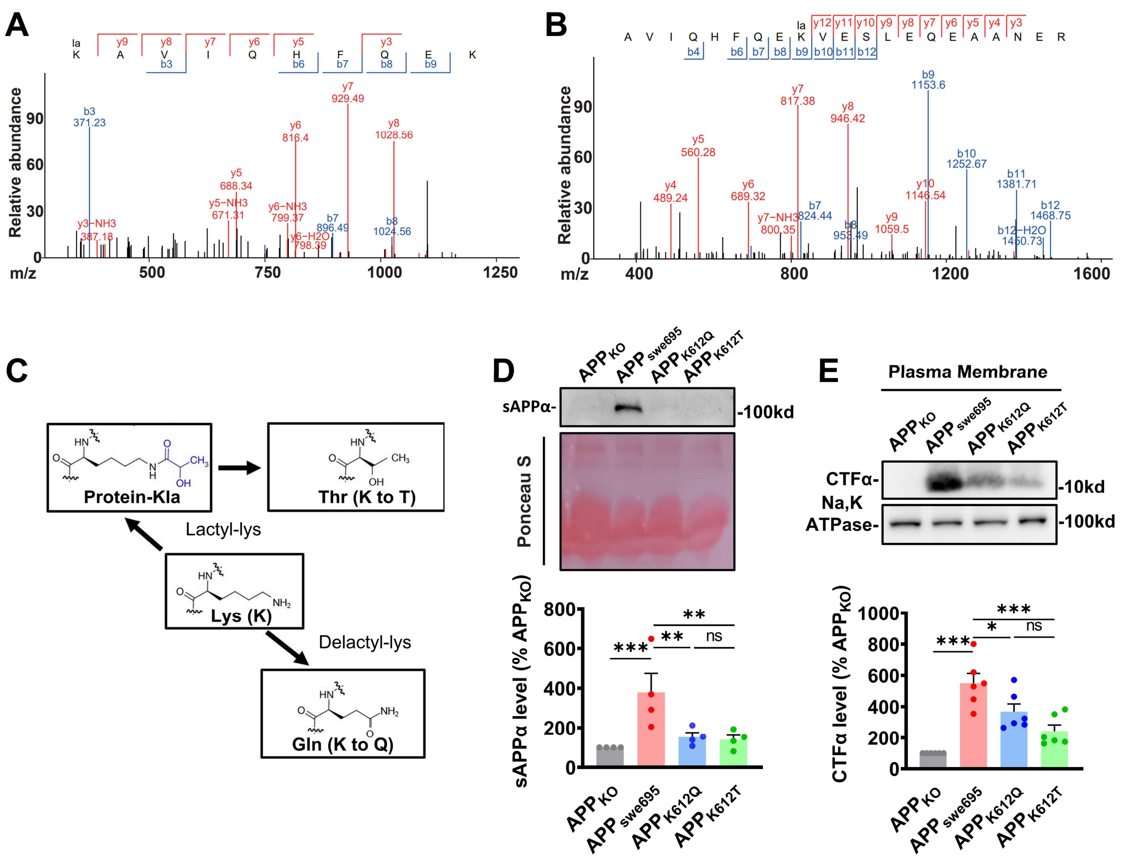

Supplemental Figure 2. Schematic map of the APP lactylation mimic mutant and the expression of APP non-amyloidosis processing-related proteins in APP-K612. (**A-B**) LC-MS/MS spectra of the lactylated peptides of APP-K354 and APP-K363. (**C**) A schematic diagram of the APP lactylation/de-lactylation mimic lysine mutant pattern. (**D**) The relative protein levels of sAPPα in cell supernatants were assessed by Western blot in APP_{KO} cells transfected with APP_{swe695}, APP_{K612Q}, and APP_{K612T} mutant plasmids (n = 4 in each group). (**E**) The relative protein levels of CTFα in plasma membrane were assessed by Western blot in APP_{KO} cells transfected with APP_{swe695}, APP_{K612Q}, and APP_{K612T} mutant plasmids (n = 6 in each group). Data were presented as mean ± SEM, *p < 0.05, **p < 0.01 and ***p < 0.001, ns, not significant. One-way ANOVA, followed by Tukey's multiple comparisons test (**D** and **E**).

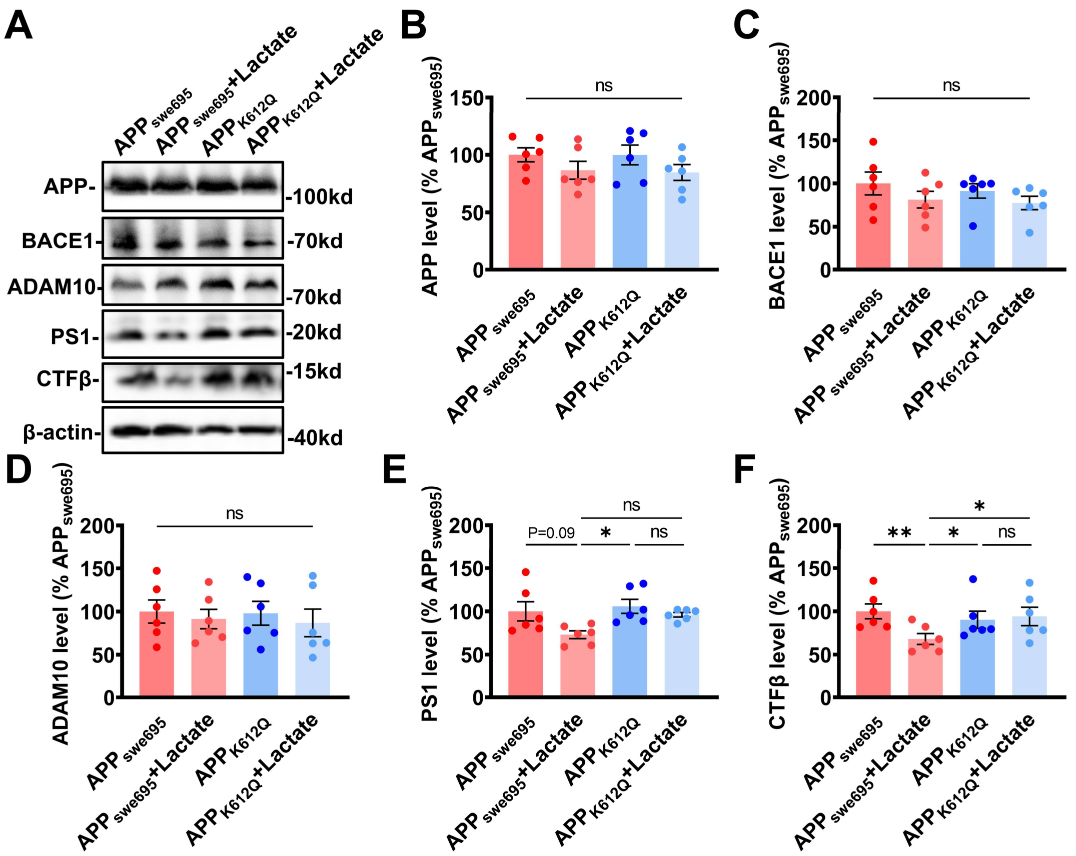

Supplemental Figure 3. L-lactate treatment does not affect the protein expression level of CTFβ in APP_{K612Q}.

(**A-F**) The relative protein levels of APP (**A**, **B**), BACE1 (**A**, **C**), ADAM10 (**A**, **D**), PS1 (**A**, **E**), and CTFβ (**A**, **F**) were assessed by Western blot in APP_{KO} cells transfected with APP_{swe695}, APP_{K612Q} mutant plasmids and treated with L-lactate (25mM/24h, n = 6 in each group). Data were presented as mean ± SEM, *$p < 0.05$, and **$p < 0.01$, ns, not significant. One-way ANOVA, followed by Tukey's multiple comparisons test (**B-F**).

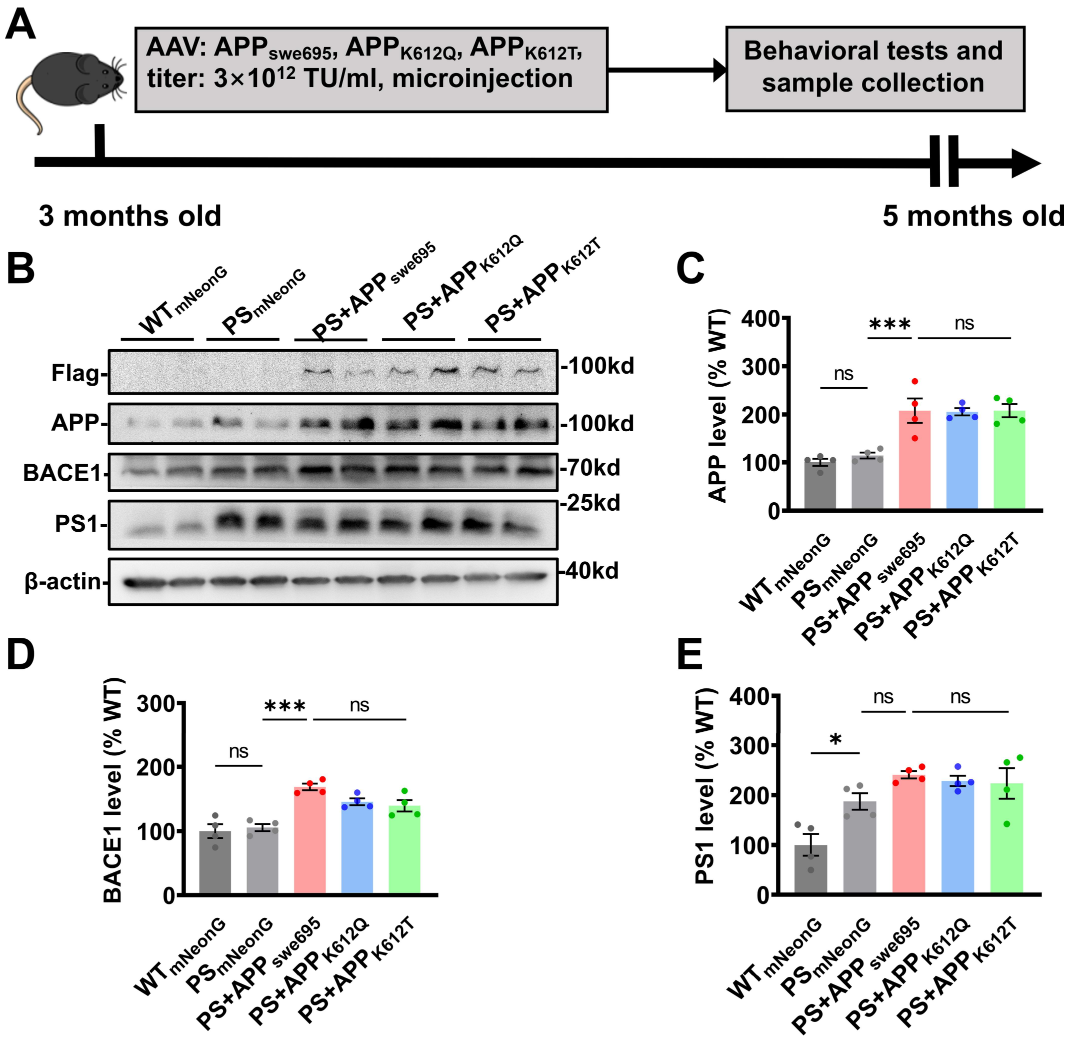

Supplemental Figure 4. Expression of APP amyloidogenic processing-related proteins in vivo by APP-K612la treatment.

(**A**) Schematic representation of the strategy for AAV treatment of bilateral hippocampal CA1 in PS45 mice. (**B-E**) The relative protein levels of APP (**B**, **C**), BACE1 (**B**, **D**), and PS1 (**B**, **E**) were assessed by Western blot in PS45 mice injected with APP_{swe695}, APP_{K612Q}, and APP_{K612T} viruses at the age of 5 months (n = 4 in each group). Data were presented as mean ± SEM, *p < 0.05, **p < 0.01, and ***p < 0.001, ns, not significant. One-way ANOVA, followed by Tukey's multiple comparisons test (**C-E**).

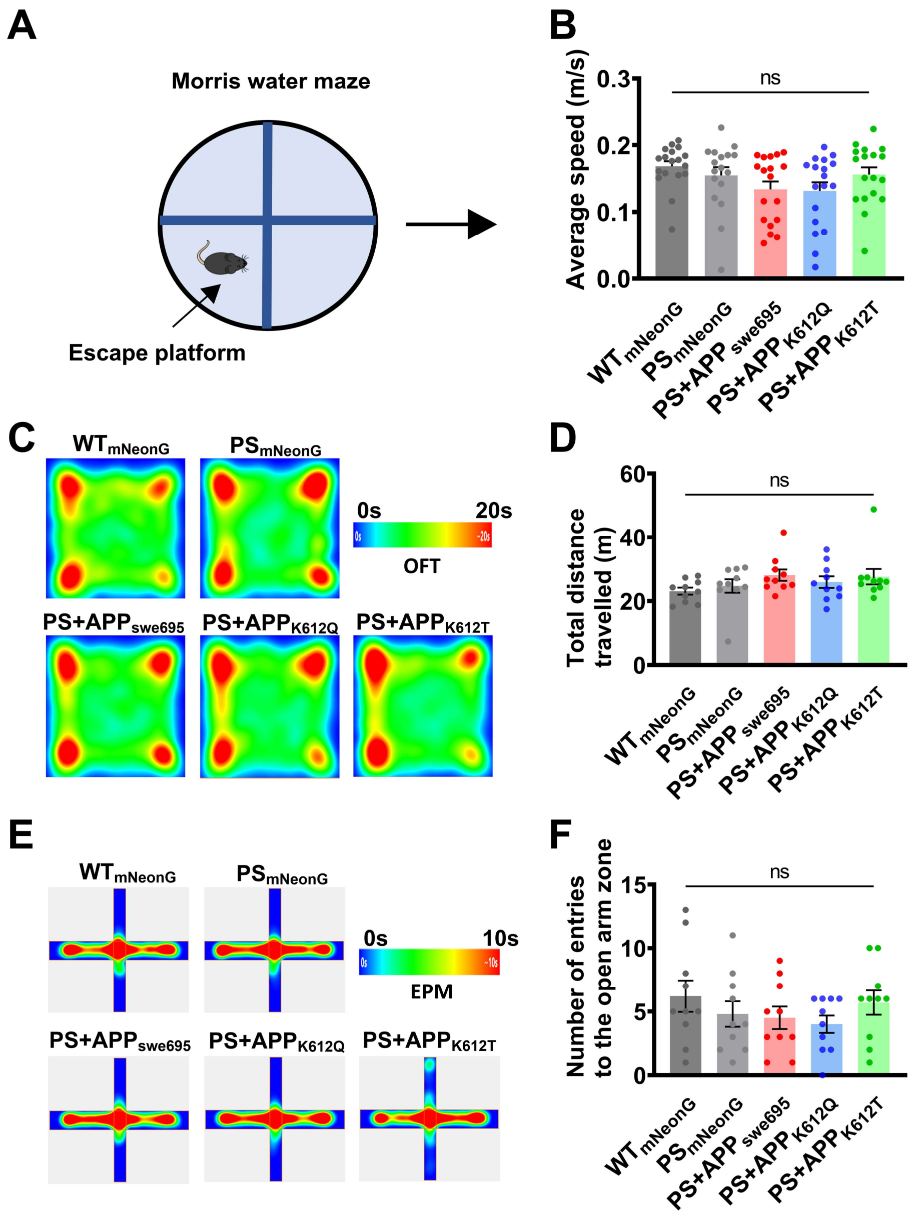

Supplemental Figure 5. No effect on spontaneous locomotion, exploratory behavior in APP-K612la mice.

(**A-B**) Average speed during spatial learning in the Morris water maze test (n=16-18 in each group). (**C**) Average heatmap during motion retrieval in the Open-field test. (**D**) Total distance traveled in the Open-field test. (**E**) Average heatmap during motion retrieval in the Elevated plus maze. (**F**) The number of entries to the open arm zone in the Elevated plus maze. Data were presented as mean ± SEM, ns, not significant. One-way ANOVA, followed by Tukey's multiple comparisons test (**B**, **D**, and **F**).

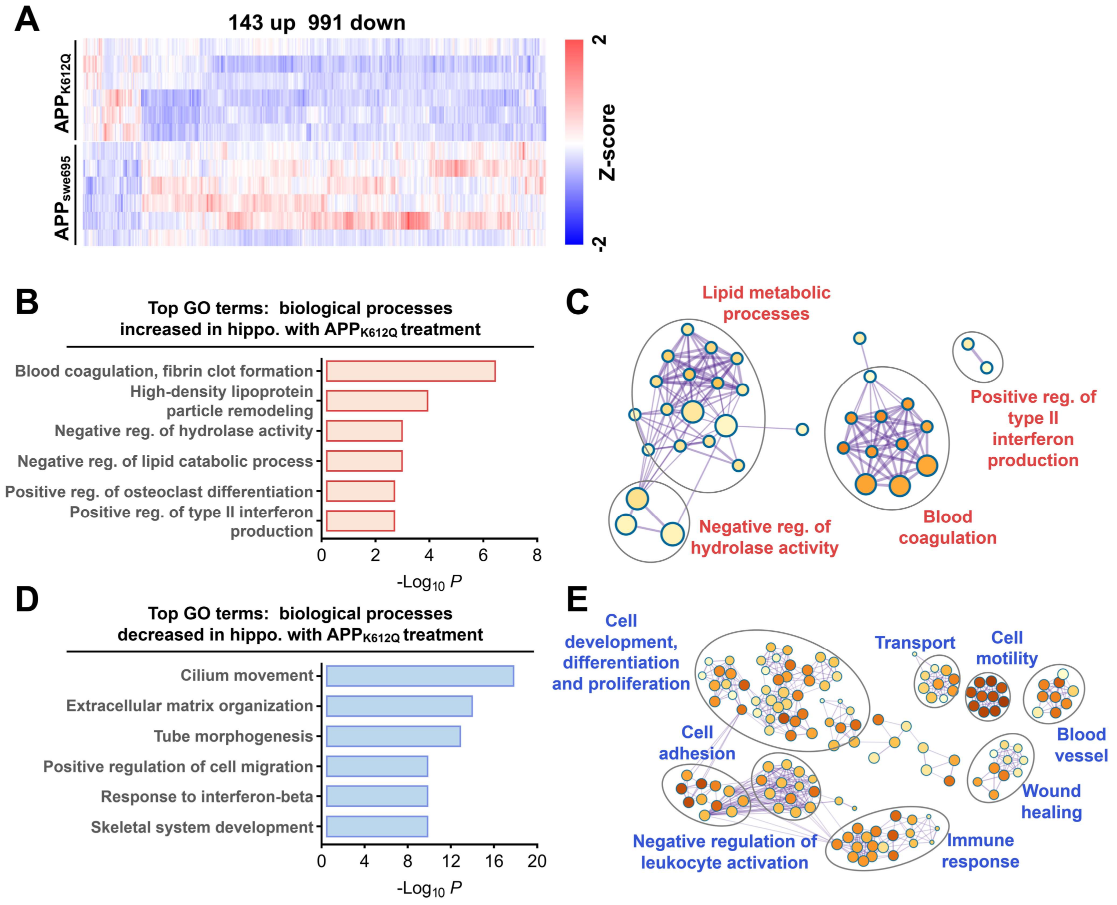

Supplemental Figure 6. Effects of APP$_{K612Q}$ on transcription of relevant biological functions in mouse hippocampus.

(**A**) Significant DEGs were identified by RNA-seq analysis of hippocampal tissues of AAV-APP$_{swe695}$, APP$_{K612Q}$ microinjected mice (log_2 FC=1.2, $p < 0.05$, n=6 in each group). (**B-C**) Top GO terms and GO enrichment map associated with up-regulated DEGs in (**A**). (**D-E**) Top GO terms and GO enrichment map associated with down-regulated DEGs in (**A**).

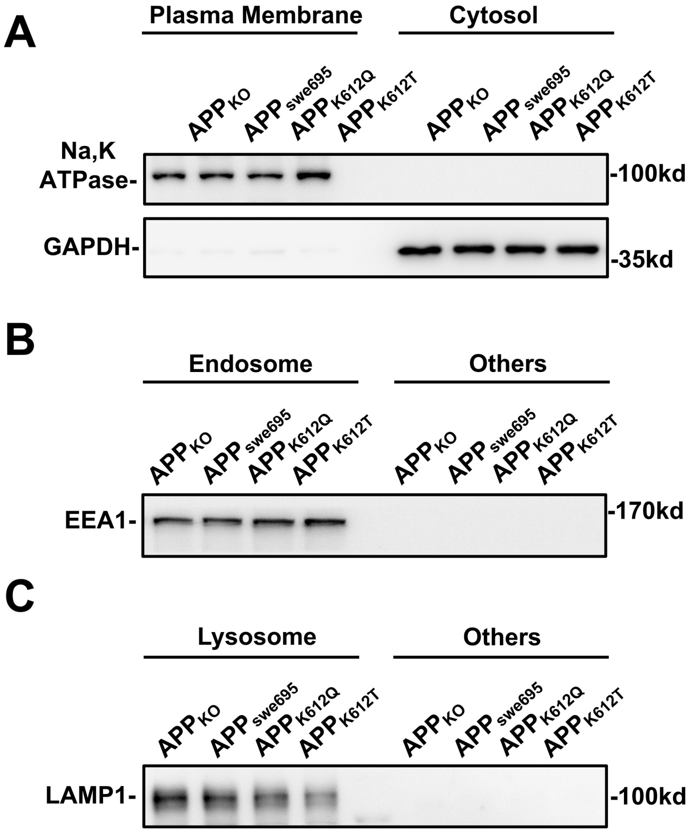

Supplemental Figure 7. Purity verification of organelle protein extraction.

(**A**) The protein levels of Na, K-ATPase, and GAPDH in the plasma membrane were assessed by Western blot. (**B**) The protein levels of EEA1 in endosomes were assessed by Western blot. (**C**) The protein levels of LAMP1 in lysosomes were assessed by Western blot.

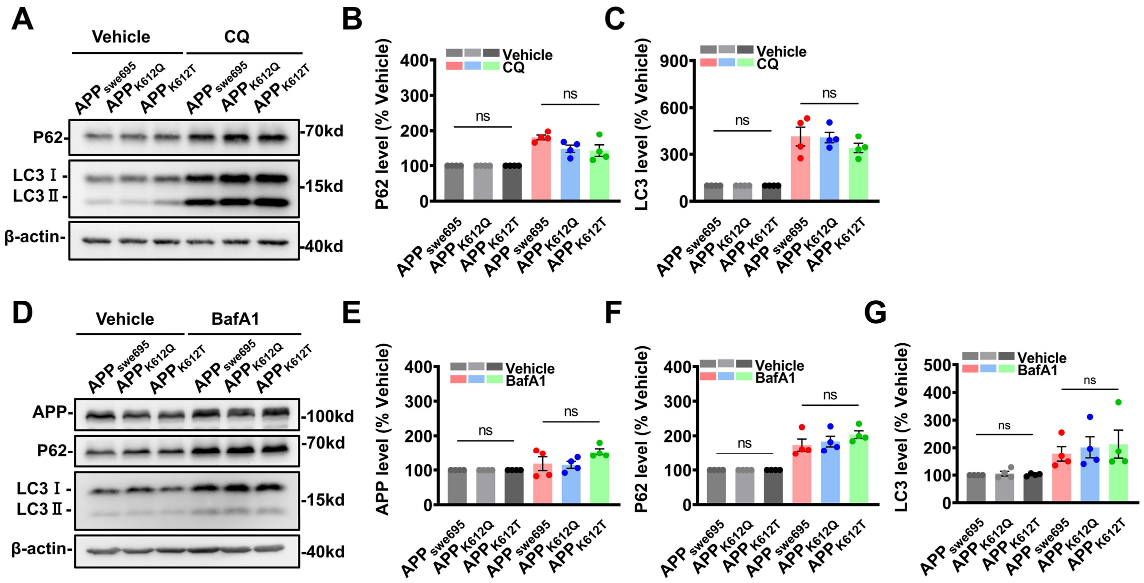

Supplemental Figure 8. APP-K612la did not affect the autophagic lysosomal pathway of APP.

(**A-C**) The relative protein levels of P62(**A**, **B**), and LC3 (**A**, **C**) treated with chloroquine (50 nM) for 24 h were assessed by Western blot in APP_{KO} cells transfected with APP_{swe695}, APP_{K612Q}, and APP_{K612T} mutant plasmids (n = 4 in each group). (**D-G**) The relative protein levels of APP (**D**, **E**), P62(**D**, **F**), and LC3 (**D**, **G**) treated with Bafilomycin A1 (BafA1, 100 nM) for 4 h were assessed by Western blot in APP_{KO} cells transfected with APP_{swe695}, APP_{K612Q}, and APP_{K612T} mutant plasmids (n = 4 in each group). Data were presented as mean ± SEM, ns, not significant. One-way ANOVA, followed by Tukey's multiple comparisons test (**B**, **C**, and **E-G**).

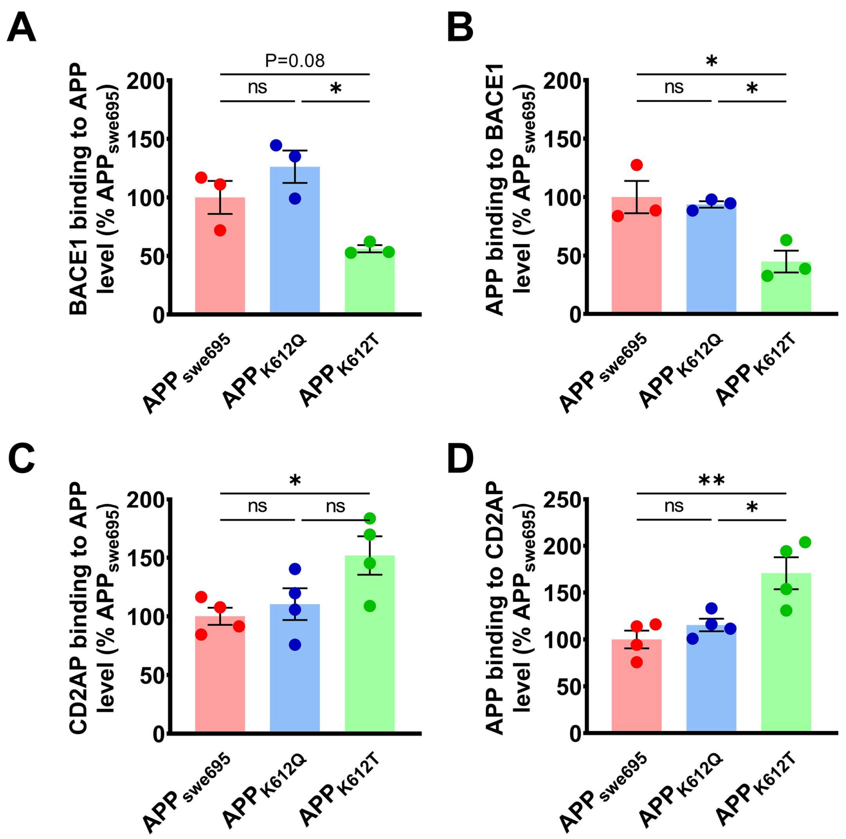

Supplemental Figure 9. Co-IP quantification of APP with BACE1 and CD2AP.

(**A-B**) Quantification of the interactions among APP_{swe695}, APP_{K612Q}, and APP_{K612T} group APP and BACE1 proteins were detected by co-immunoprecipitation in endosomal protein lysates (n = 3 per group). (**C-D**) Quantification of the interactions among APP_{swe695}, APP_{K612Q}, and APP_{K612T} group APP and CD2AP proteins were detected by co-immunoprecipitation in endosomal protein lysates (n = 4 per group). Data were presented as mean ± SEM, *$p < 0.05$, **$p < 0.01$, ns, not significant. One-way ANOVA, followed by Tukey's multiple comparisons test (**A-D**).

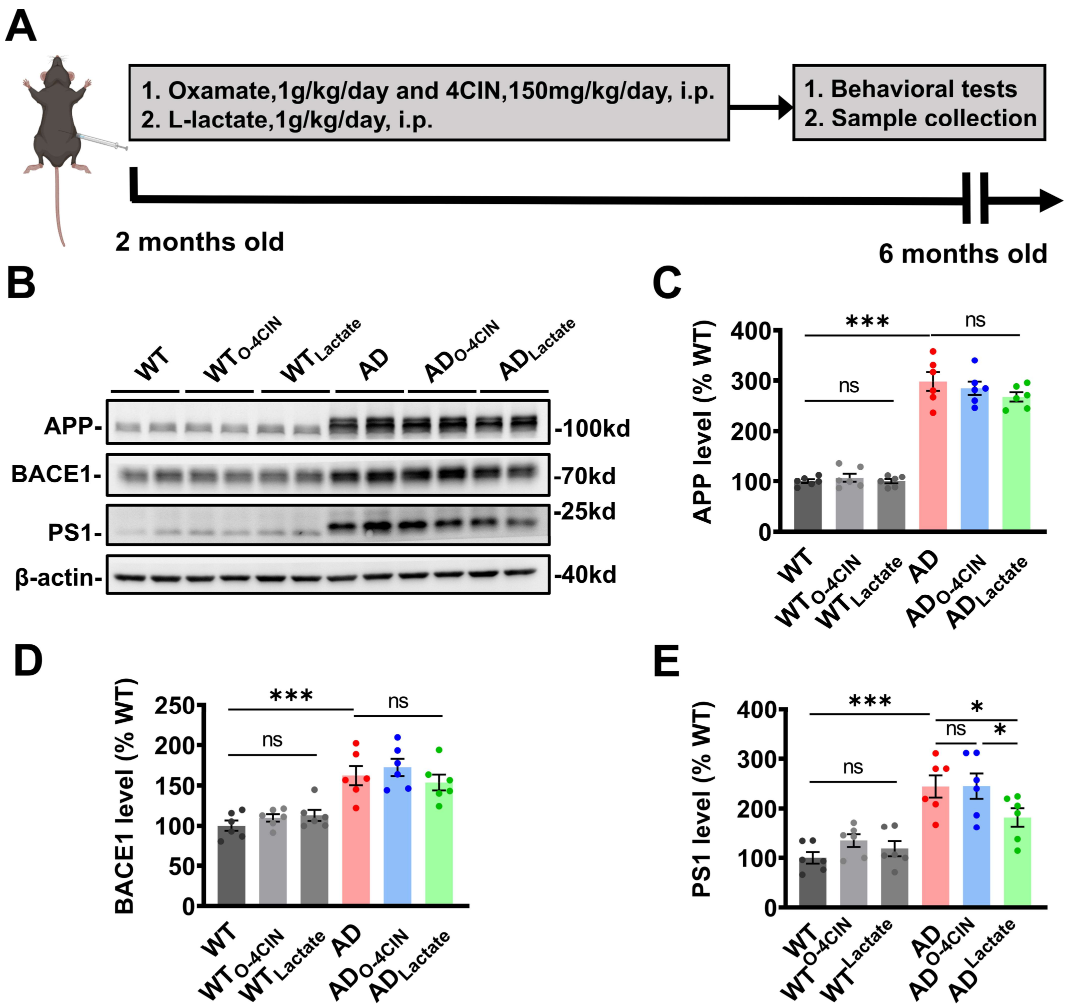

Supplemental Figure 10. Expression of APP amyloidogenic processing-related proteins in L-lactate treated AD mice.

(**A**) Schematic showing the strategy for administering L-lactate or O-4CIN treatment in APP23/PS45 mice. (**B-E**) The relative protein levels of APP (**A**, **B**), BACE1 (**A**, **C**), and PS1 (**A**, **D**) were assessed by Western blot in the hippocampus of WT and APP23/PS45 mice treated with L-lactate or O-4CIN at the age of 6 months (n = 6 in each group). Data were presented as mean ± SEM, *p < 0.05 and ***p < 0.001, ns, not significant. One-way ANOVA, followed by Tukey's multiple comparisons test (**C-E**).

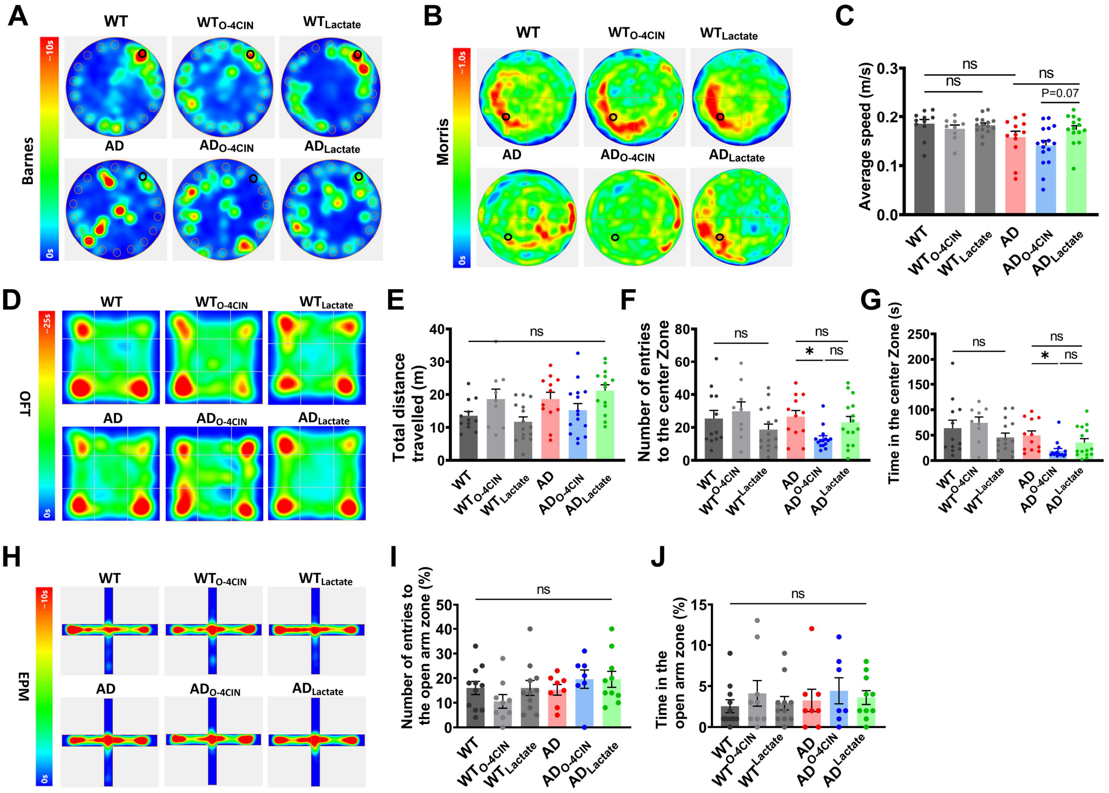

Supplemental Figure 11. No effect on spontaneous locomotion, exploratory behavior in in L-lactate treated AD mice.

(**A**) Average heatmap during memory retrieval in the Barnes maze test. (**B-C**) Average heatmap during memory retrieval (**B**) and average speed (**C**) during spatial learning in the Morris water maze test (n=9-15 in each group). (**D**) Average heatmap during motion retrieval in the Open-field test. (**E-G**) Total distance traveled (**E**) and the number (**F**) and time (**G**) of entries to the center zone in the Open-field test. (**H**) Average heatmap during motion retrieval in the Elevated plus maze. (**I-J**) The number (**I**) and time (**J**) of entries to the open arm zone in the Elevated plus maze. Data were presented as mean ± SEM, *p < 0.05, ns, not significant. Two-way ANOVA, followed by Tukey's multiple comparisons test (**C**, **E-G**, and **I-J**).
