# Supplementary material for: APP lysine 612 lactylation ameliorates amyloid pathology and memory decline in Alzheimer’s disease
Source: J Clin Invest. 2025 Jan 2;135(1):e184656. doi: 10.1172/JCI184656 (PMC11684803; doi:10.1172/JCI184656)

Figure 1A.

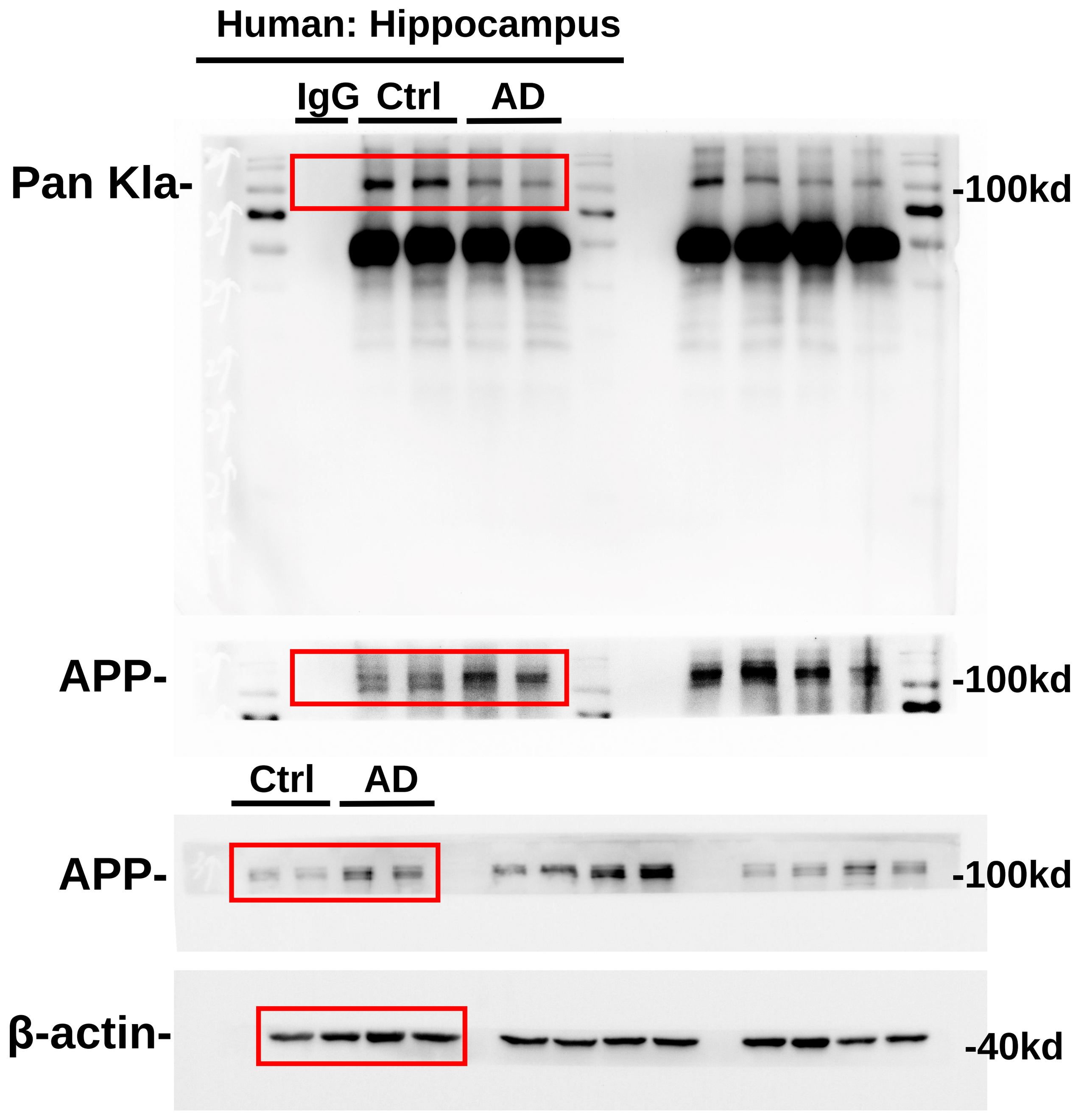

Figure 1C.

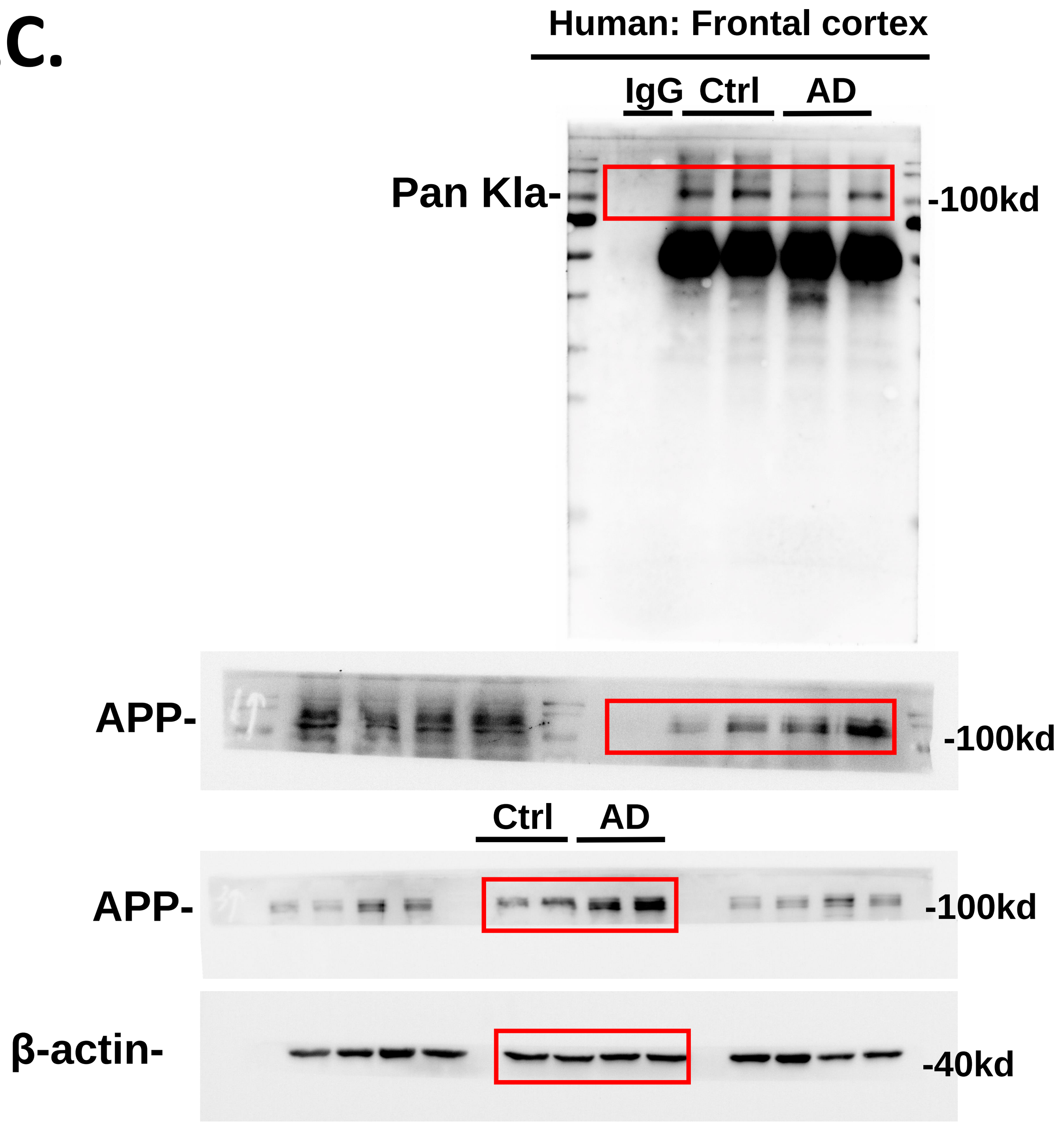

Figure 1E.

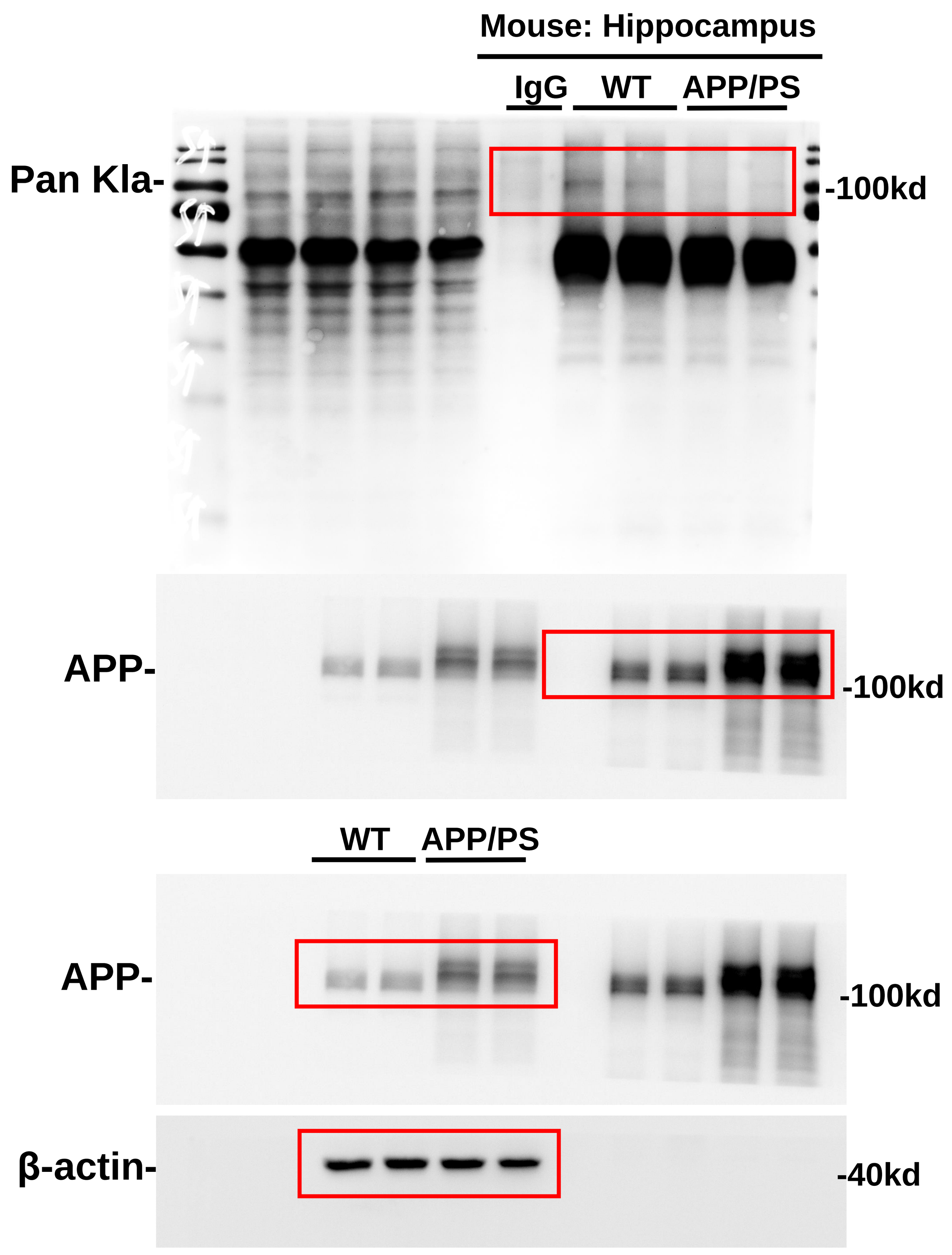

Figure 1G.

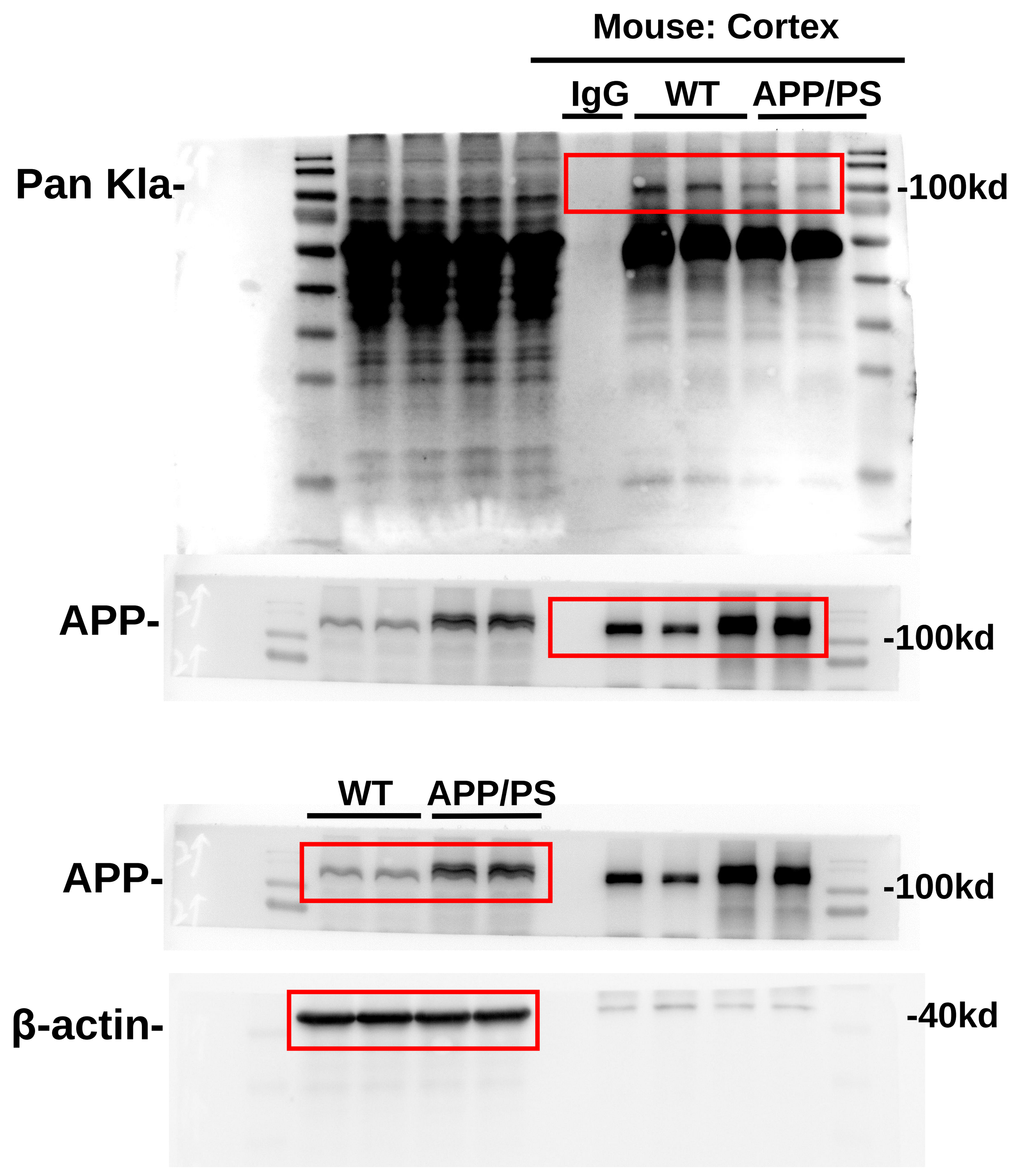

Figure 1L.

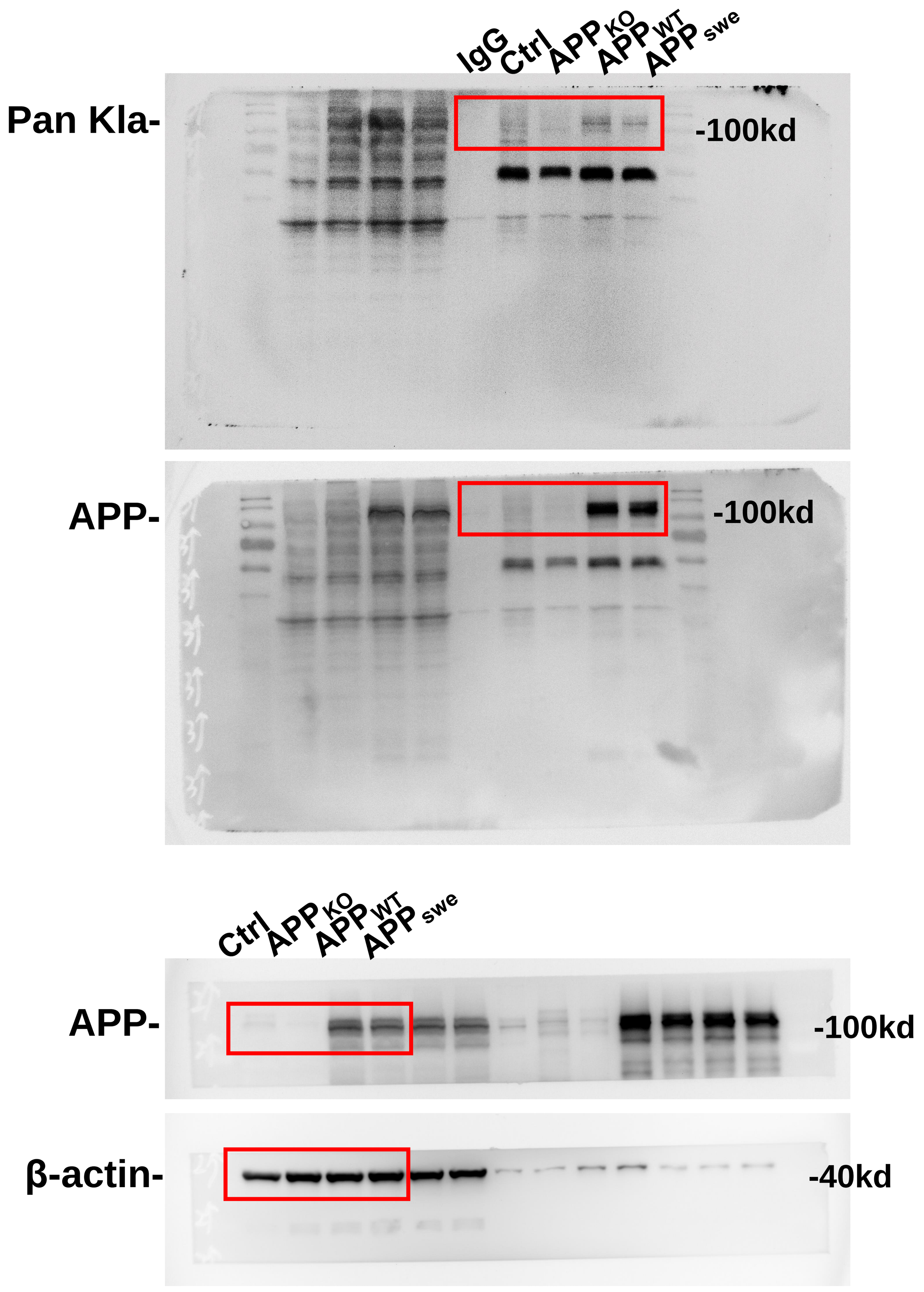

Figure 1J.

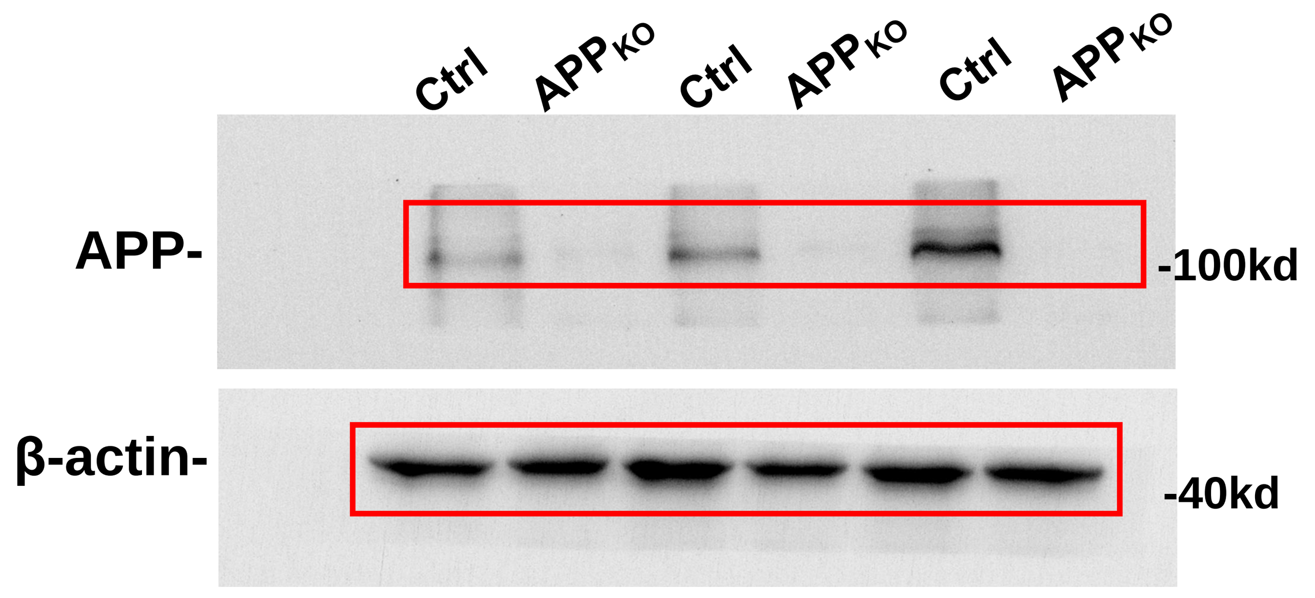

Figure 2E.

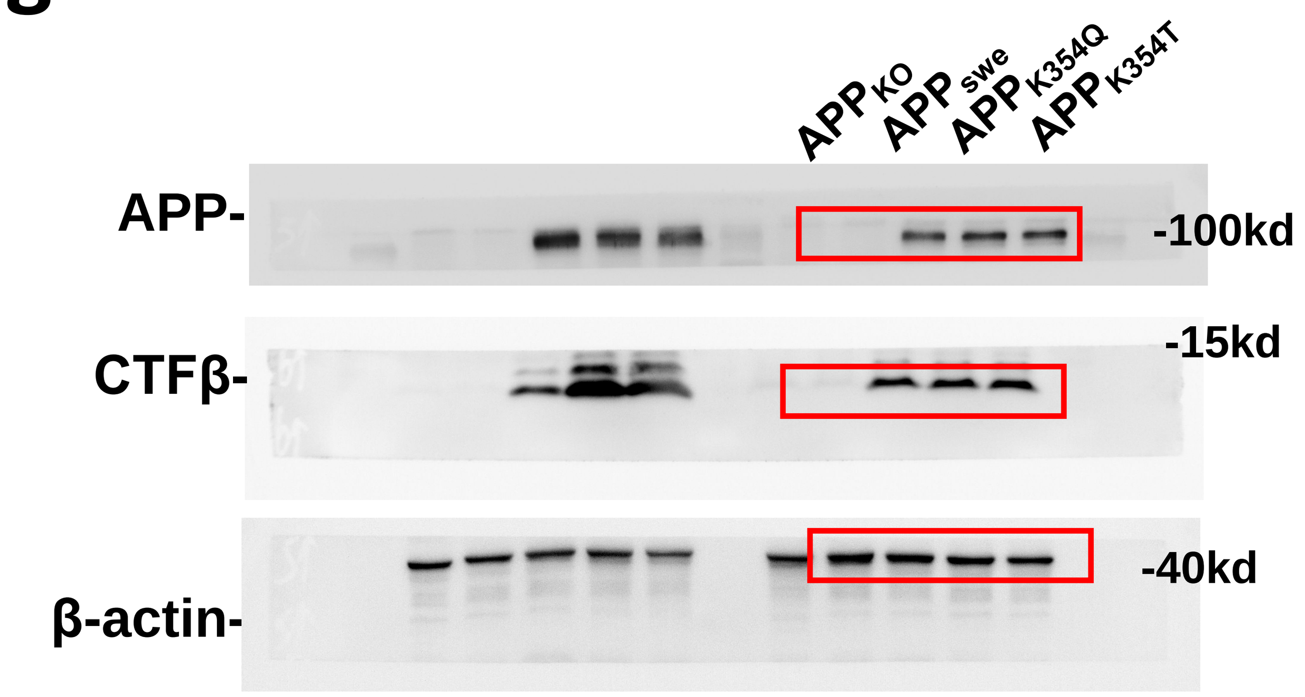

Figure 2H.

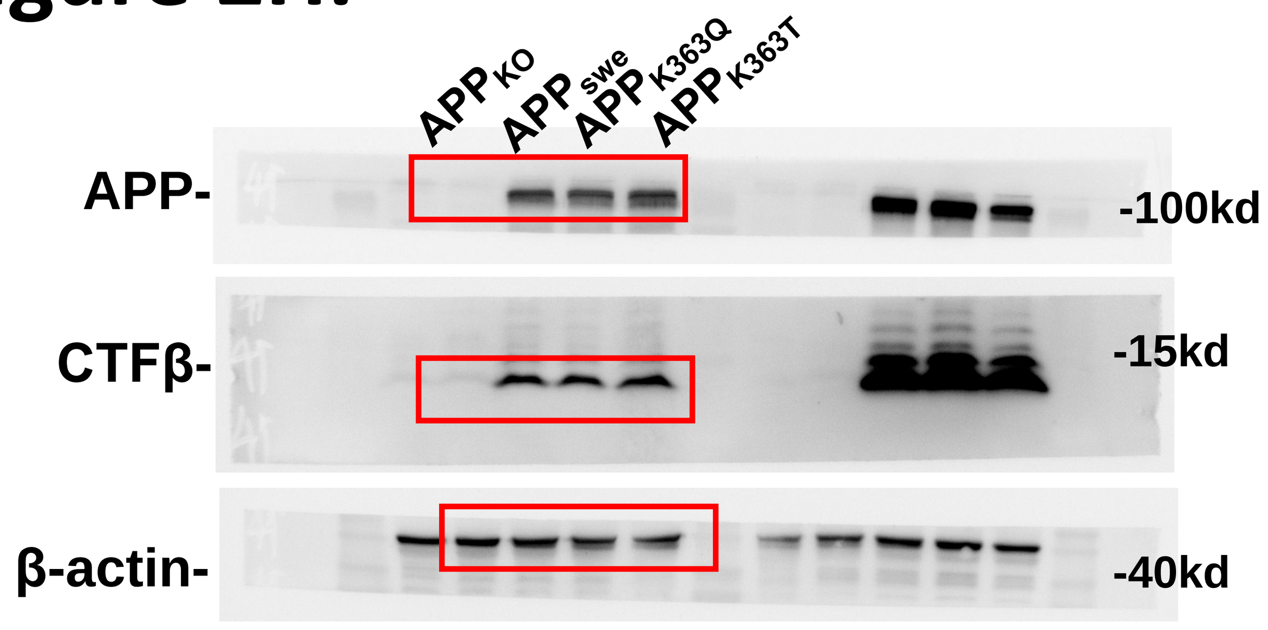

Figure 3D.

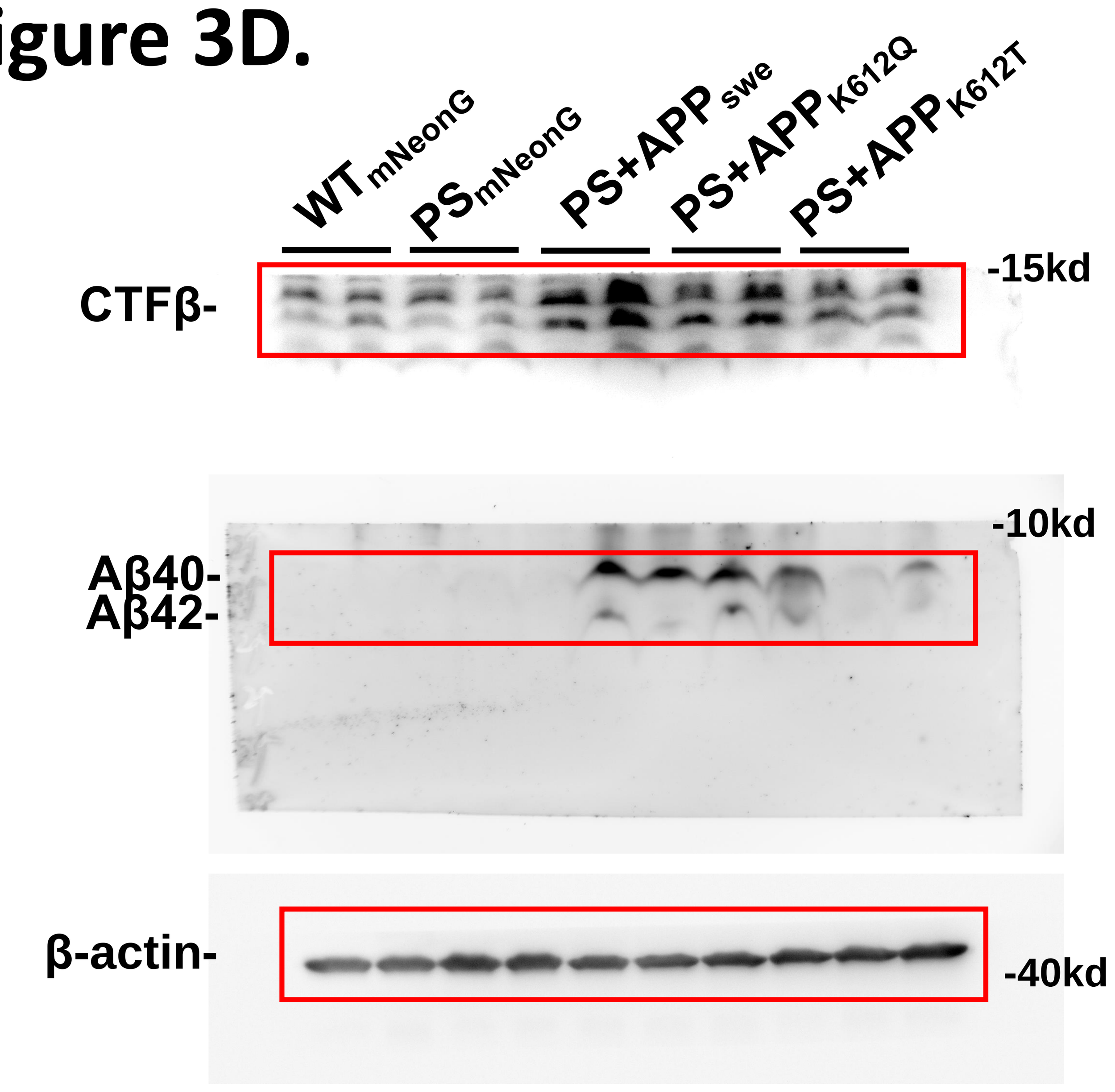

Figure 2K.

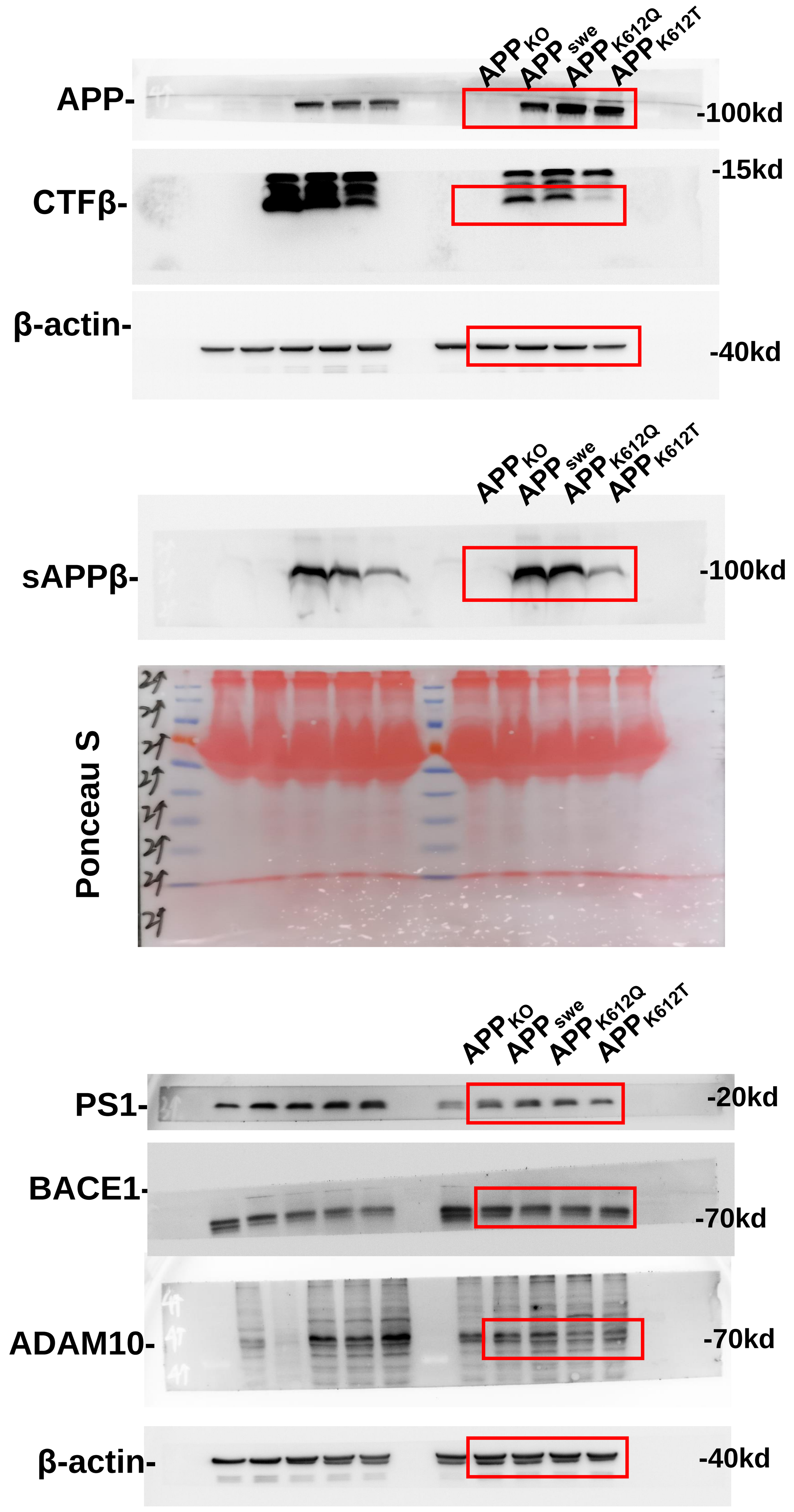

Figure 6A

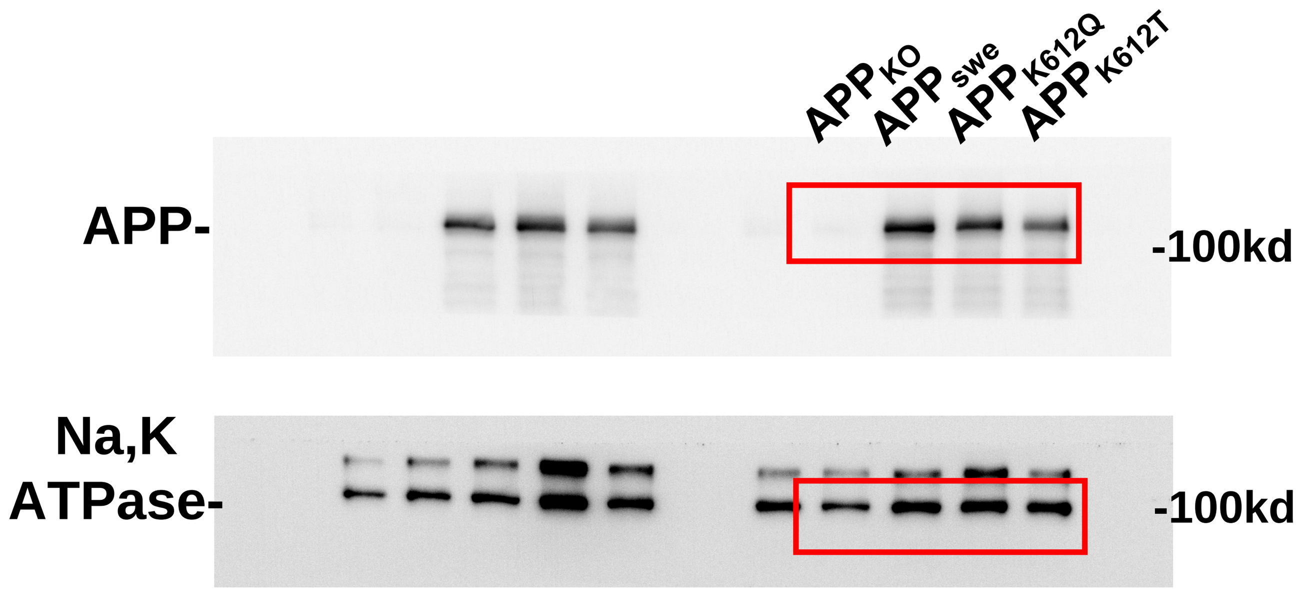

Figure 6G

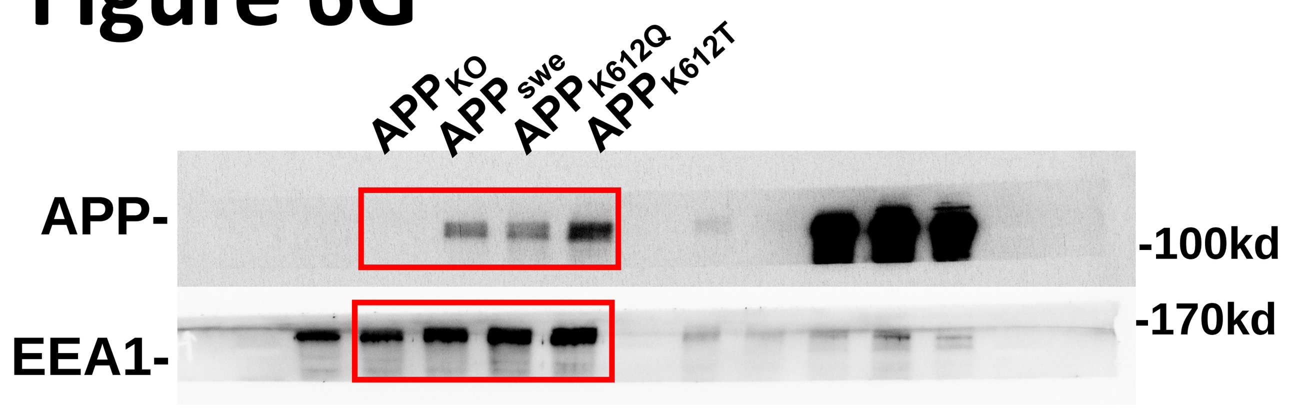

Figure 6D

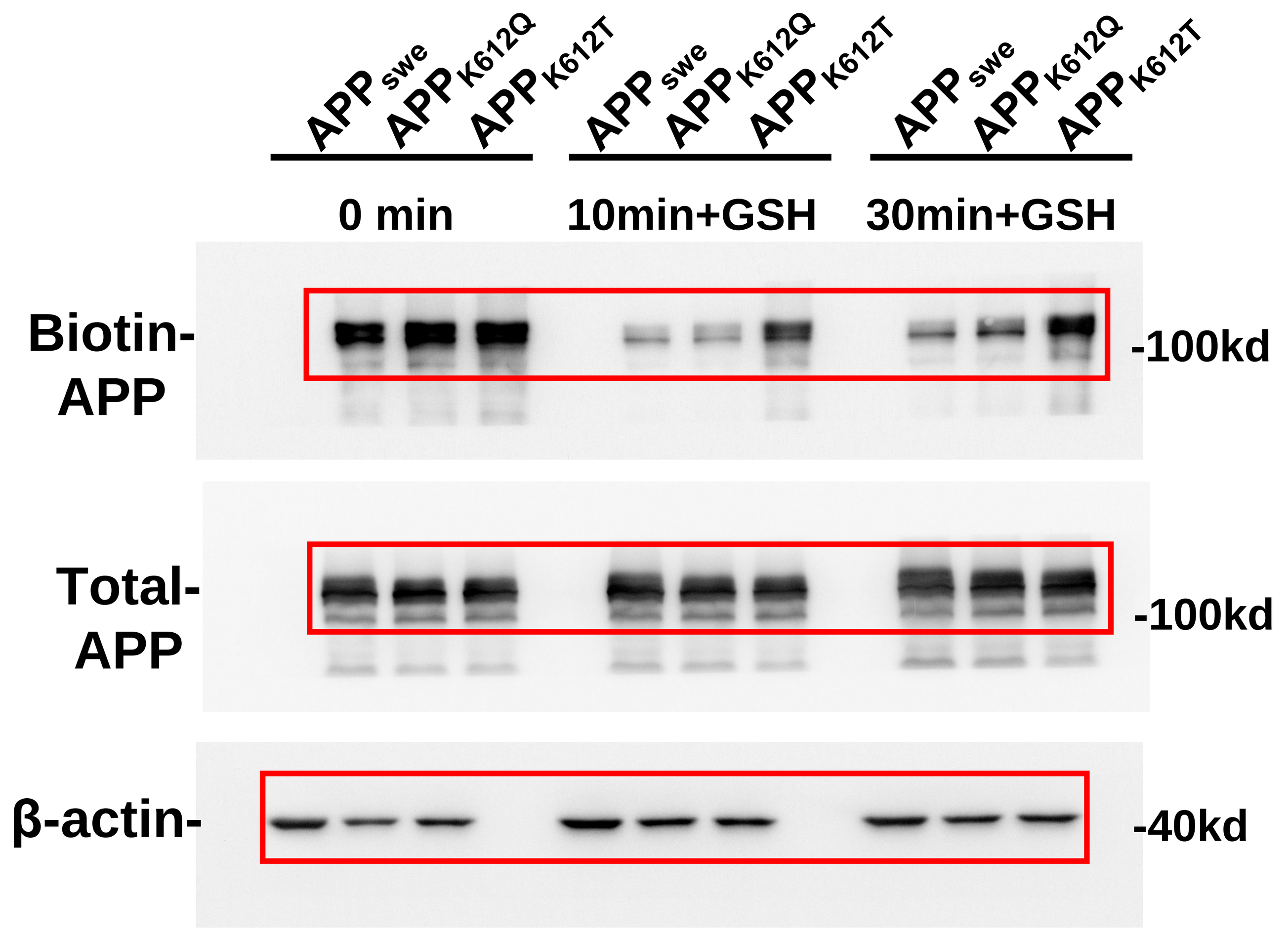

Figure 6H

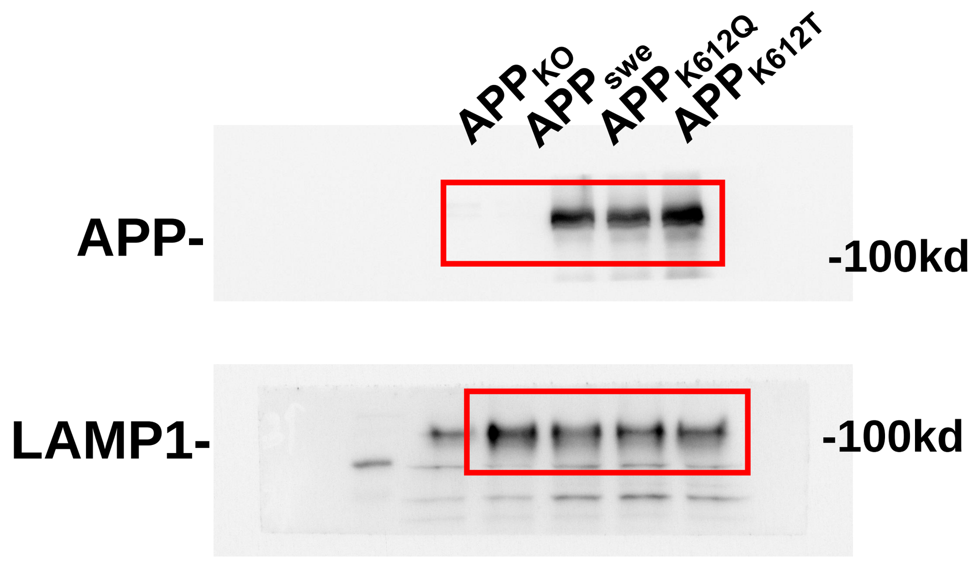

Figure 7A.

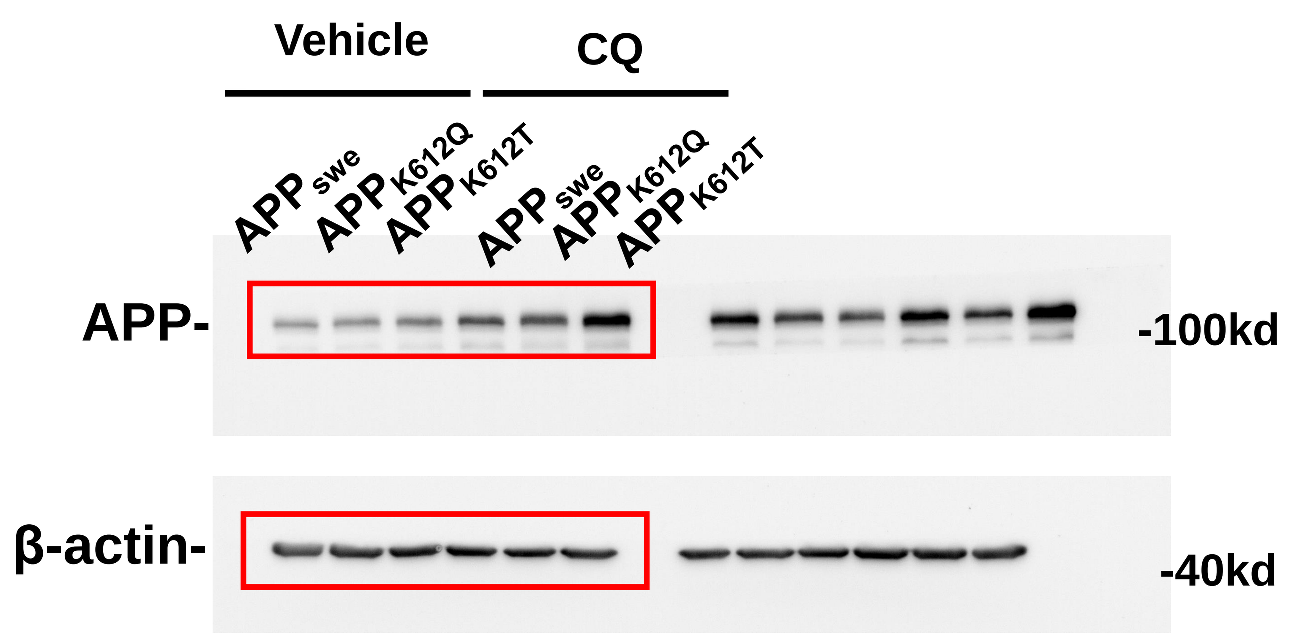

Figure 7D.

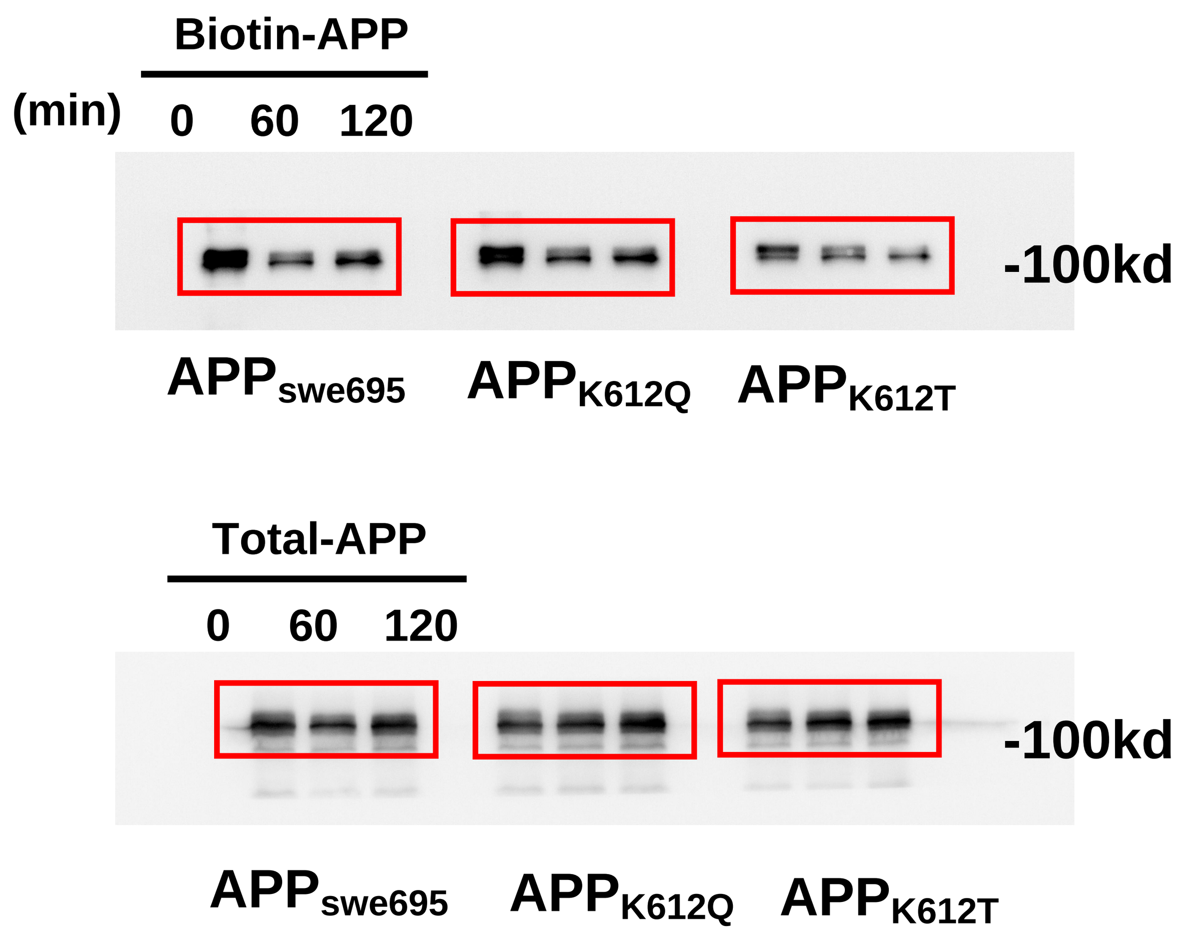

Figure 7E.

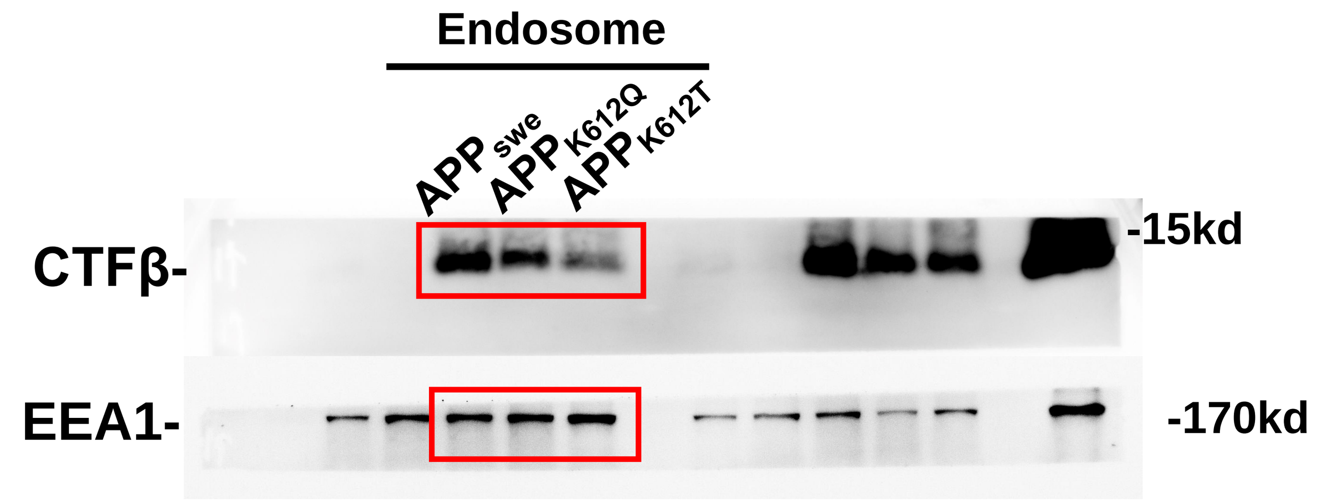

Figure 7F.

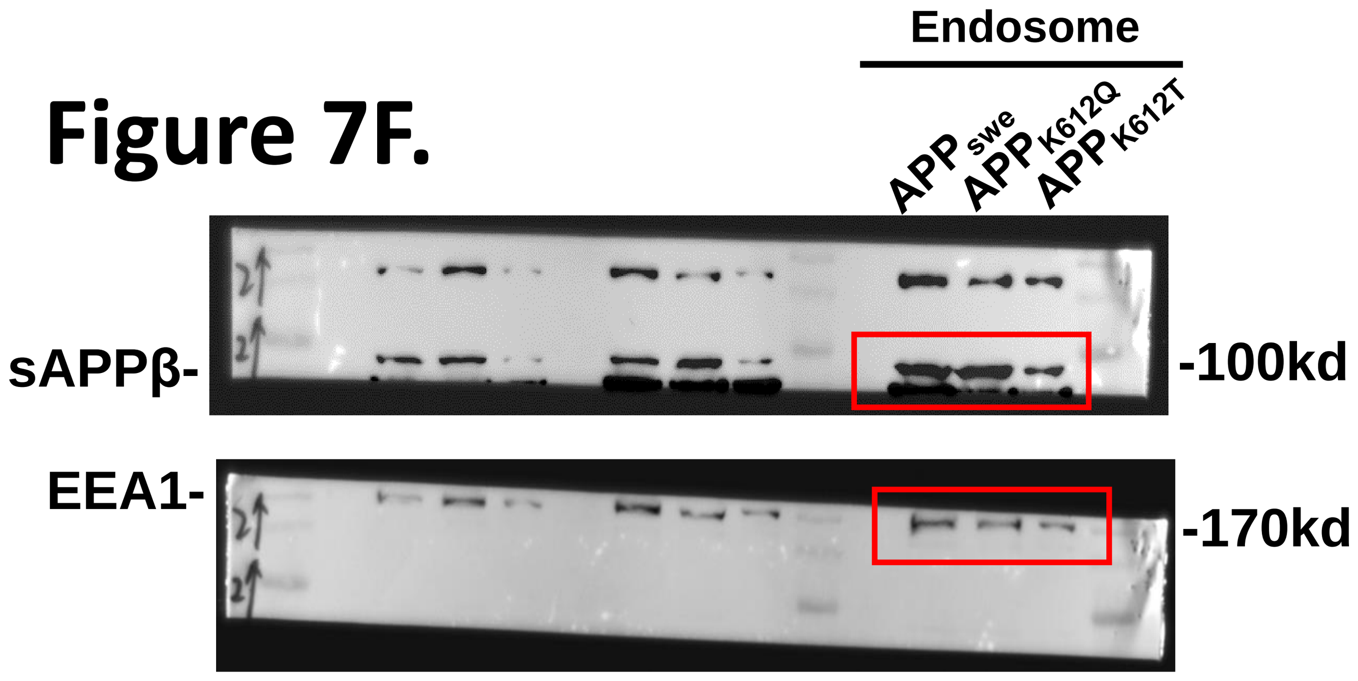

Figure 7G.

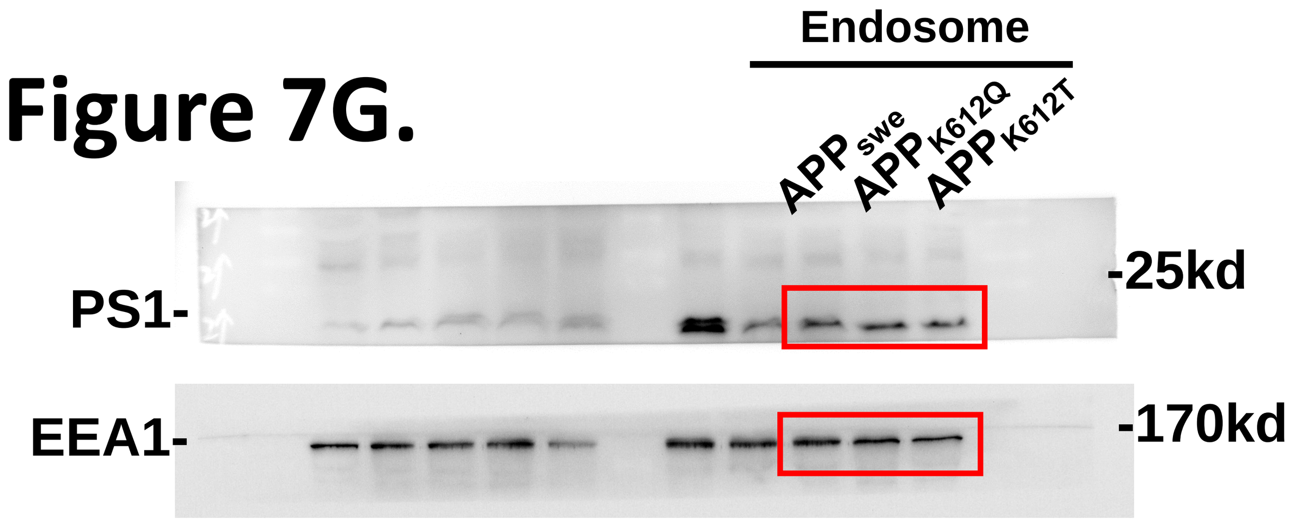

Figure 7H.

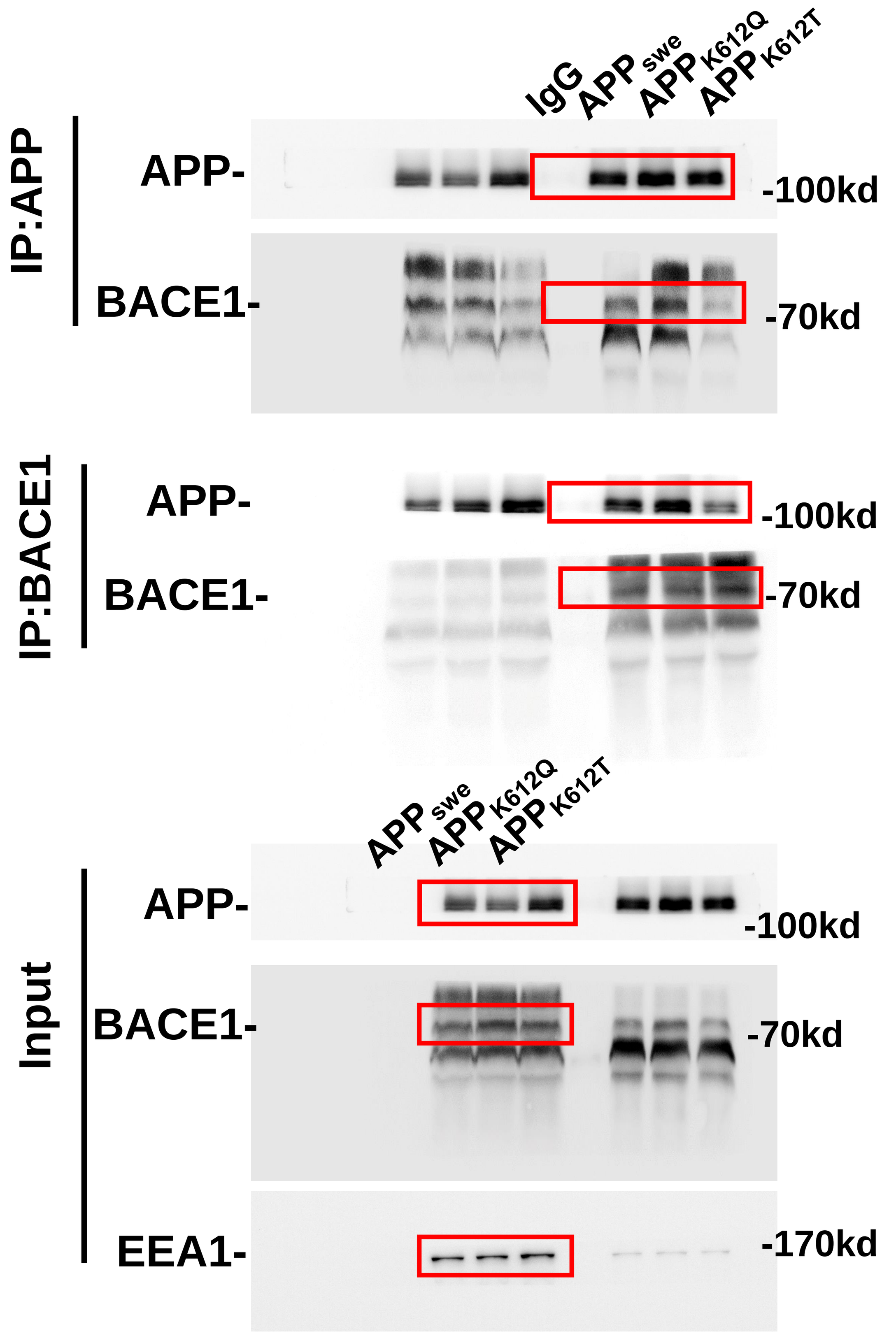

Figure 7I.

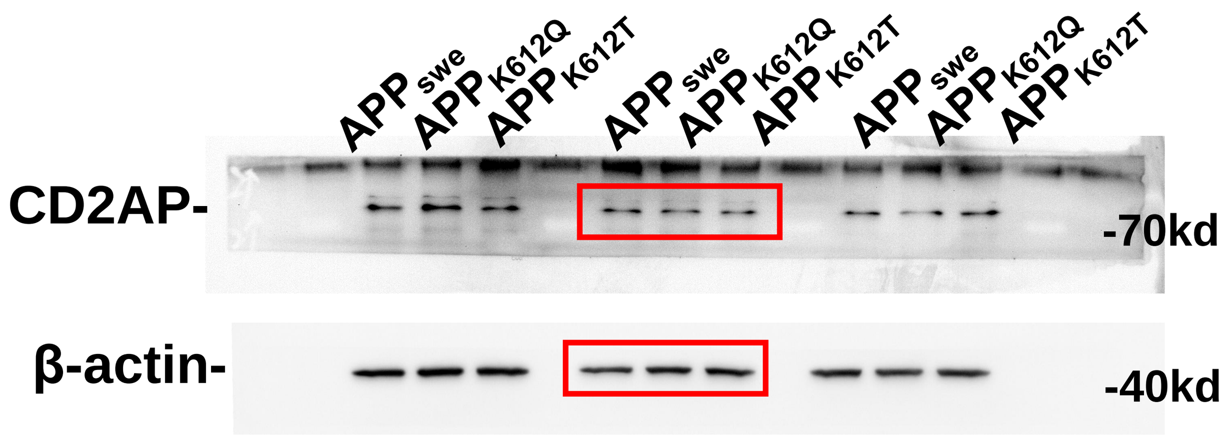

Figure 7J.

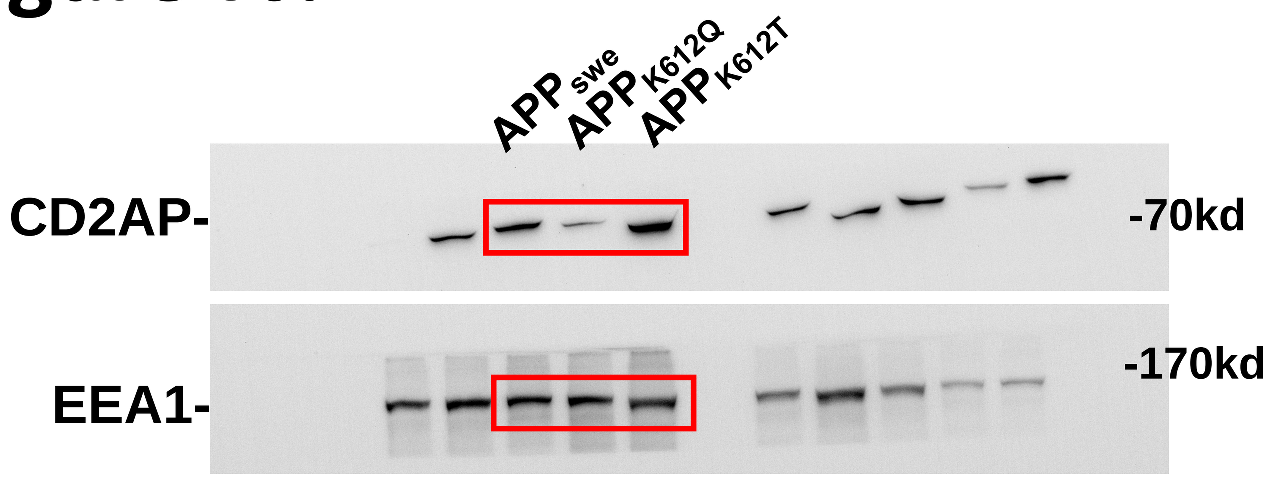

Figure 7K.

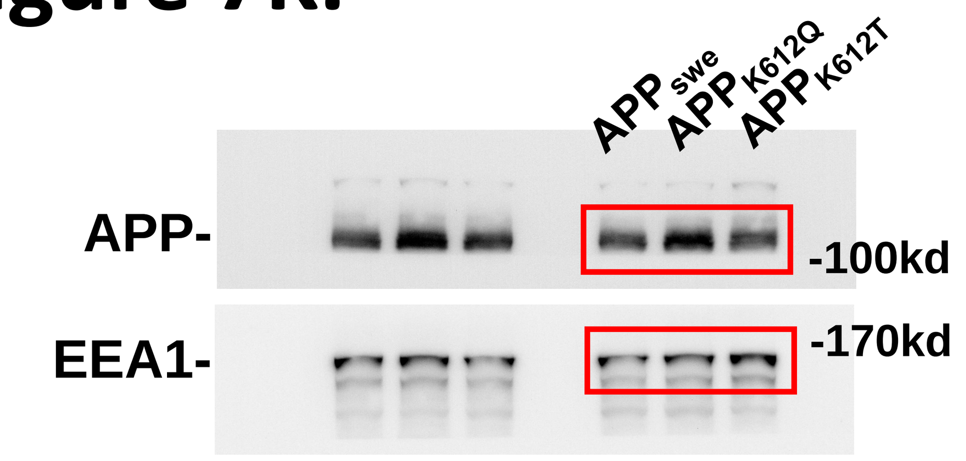

Figure 7L.

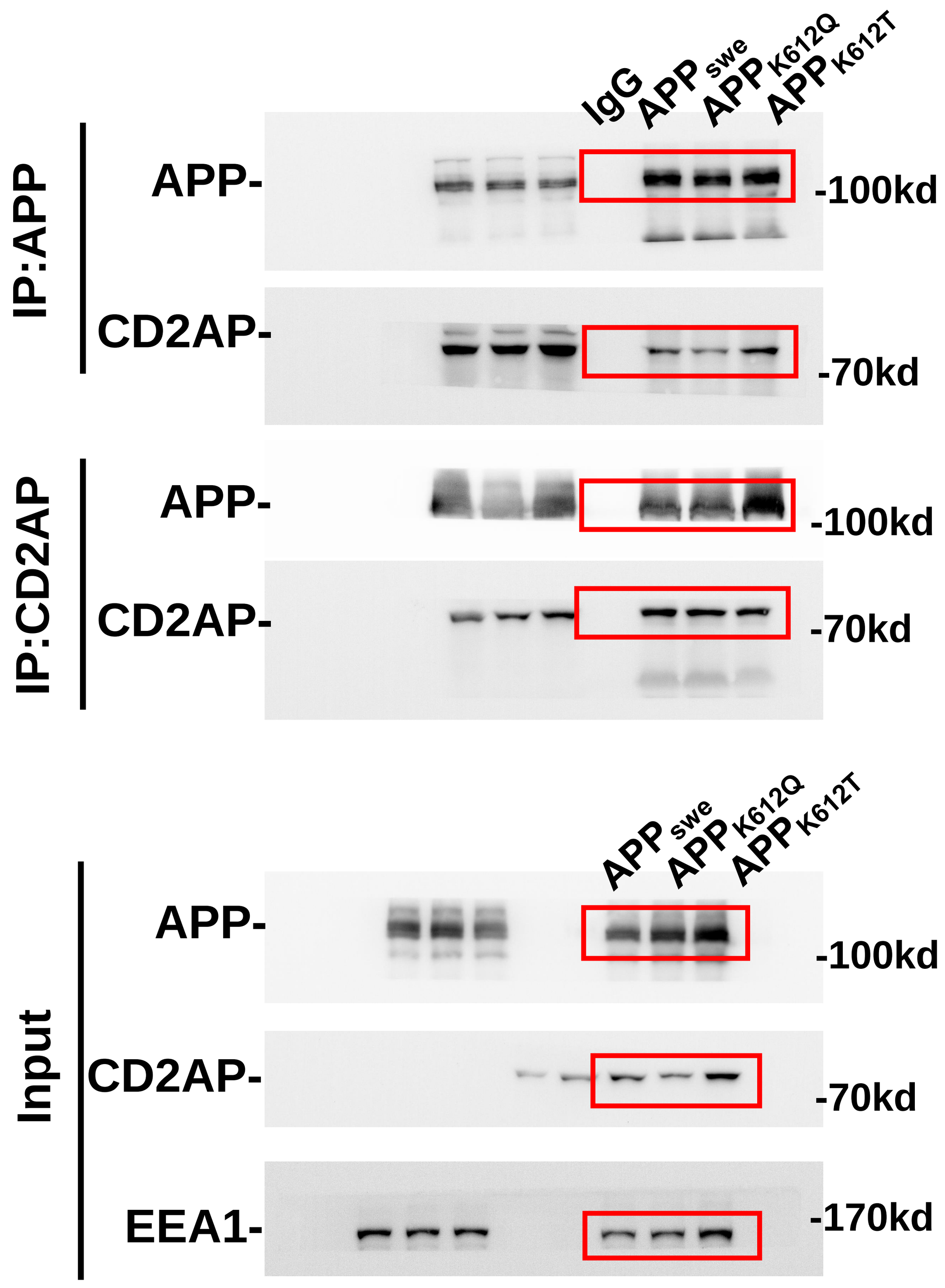

Figure 8A.

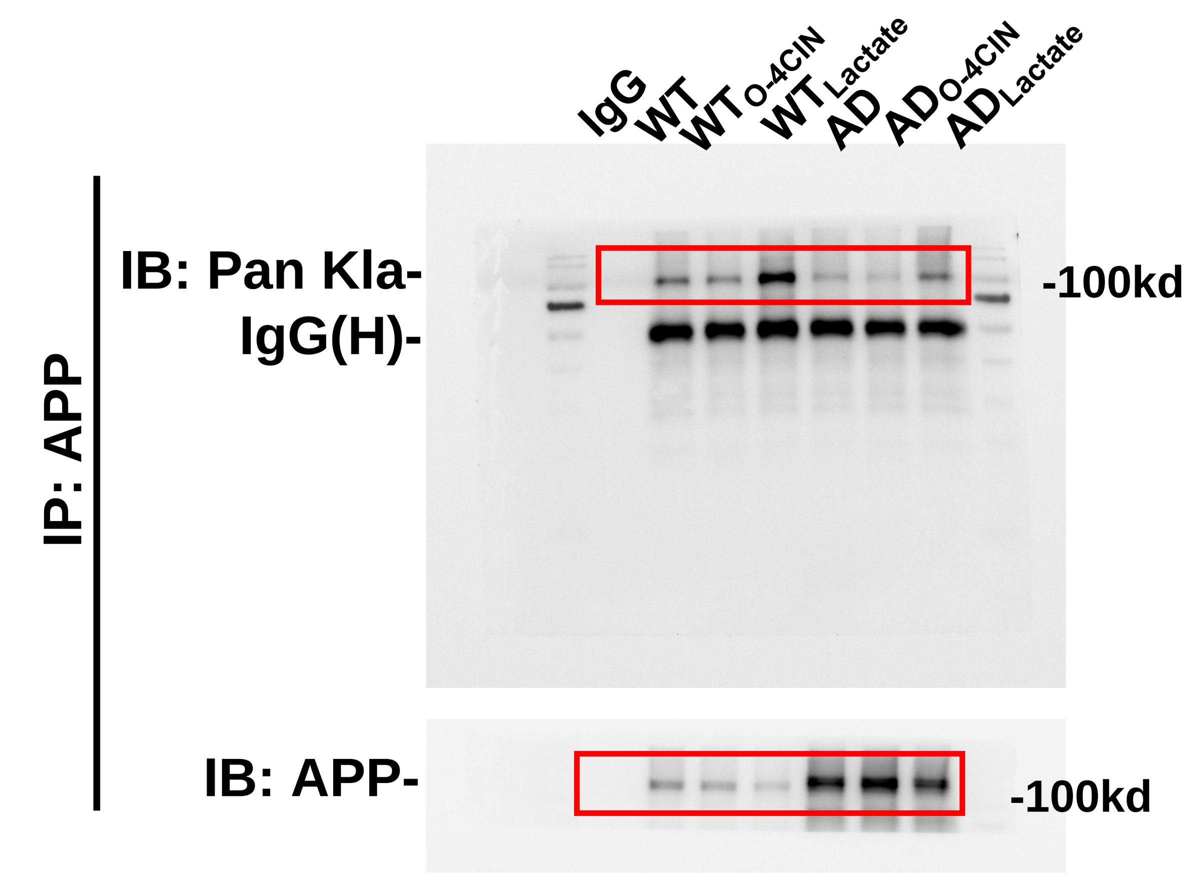

Figure 8D.

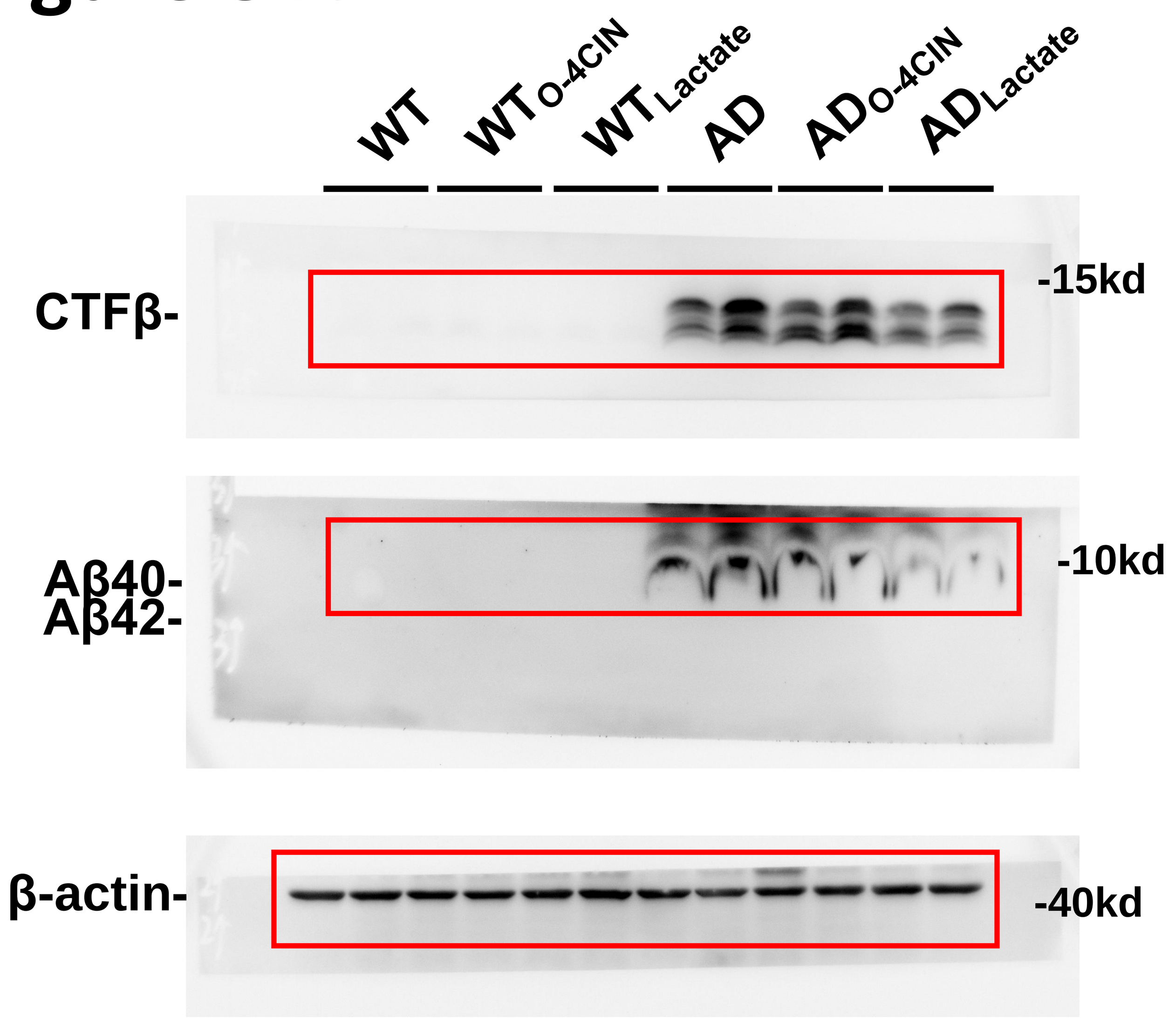

Supplemental Figure 1A

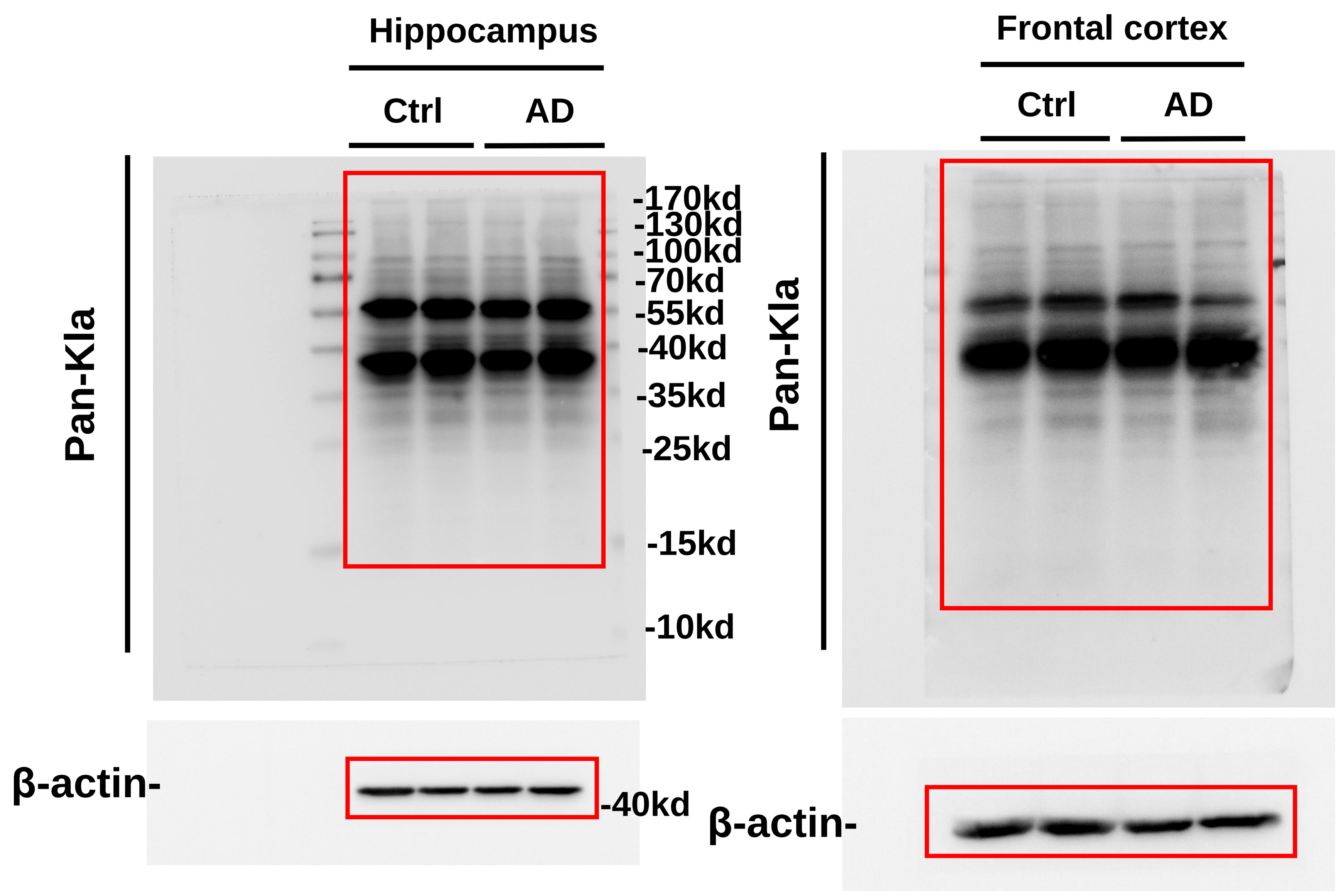

Supplemental Figure 1B

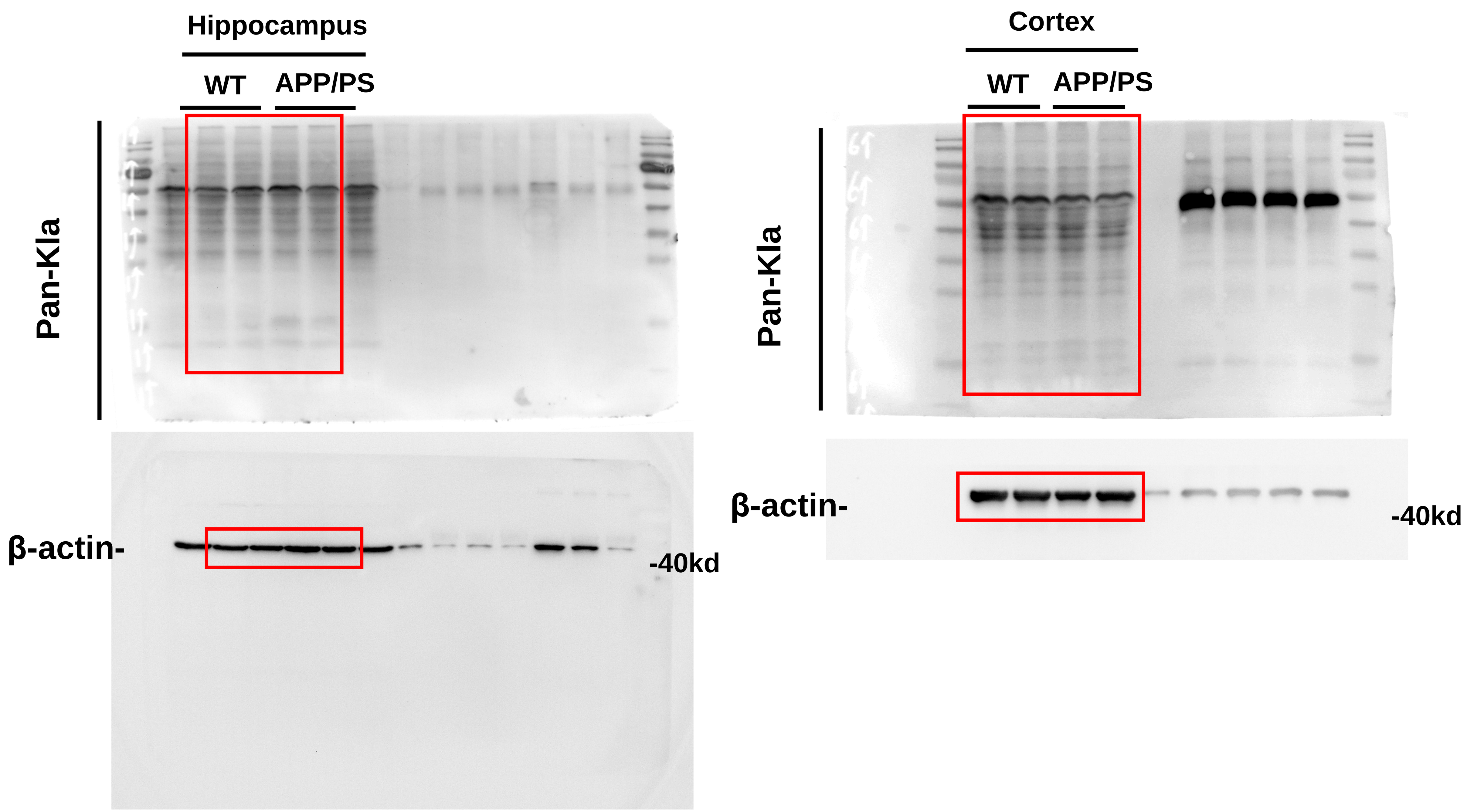

Supplemental Figure 2D

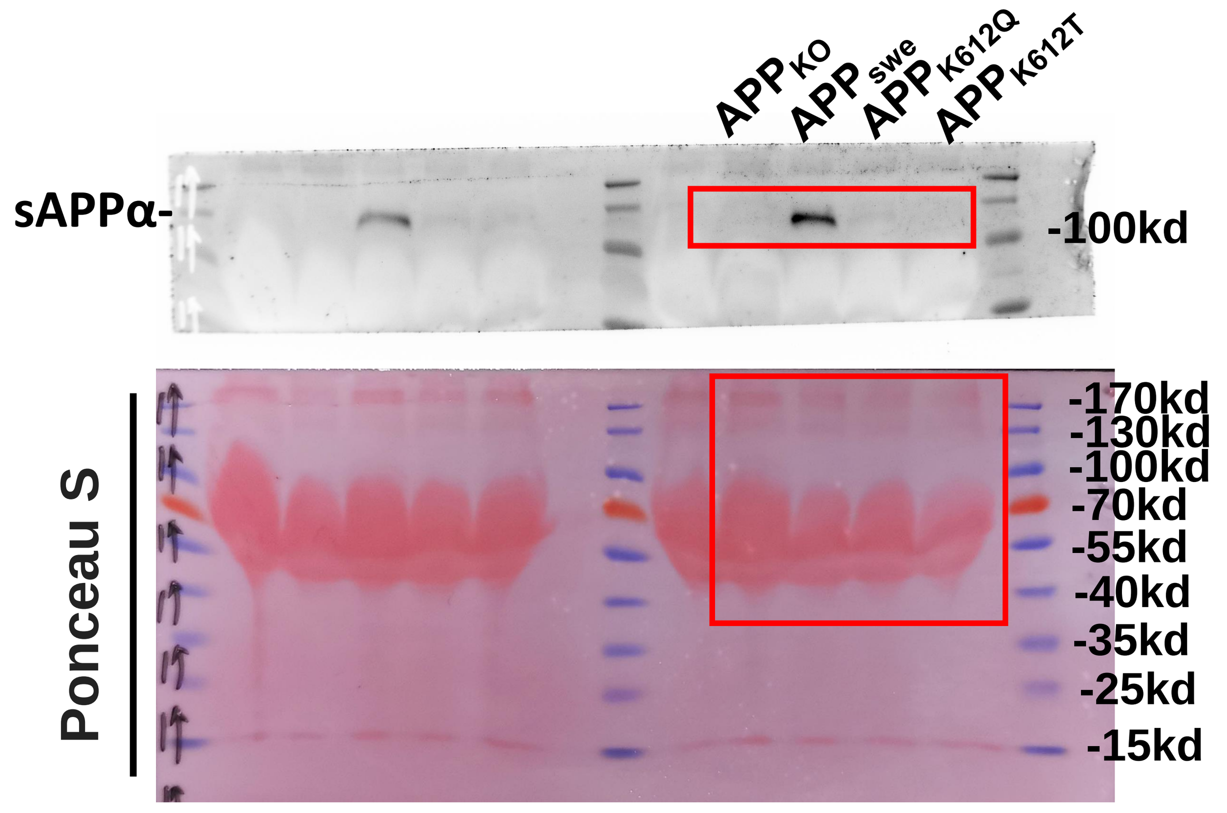

Supplemental Figure 2E

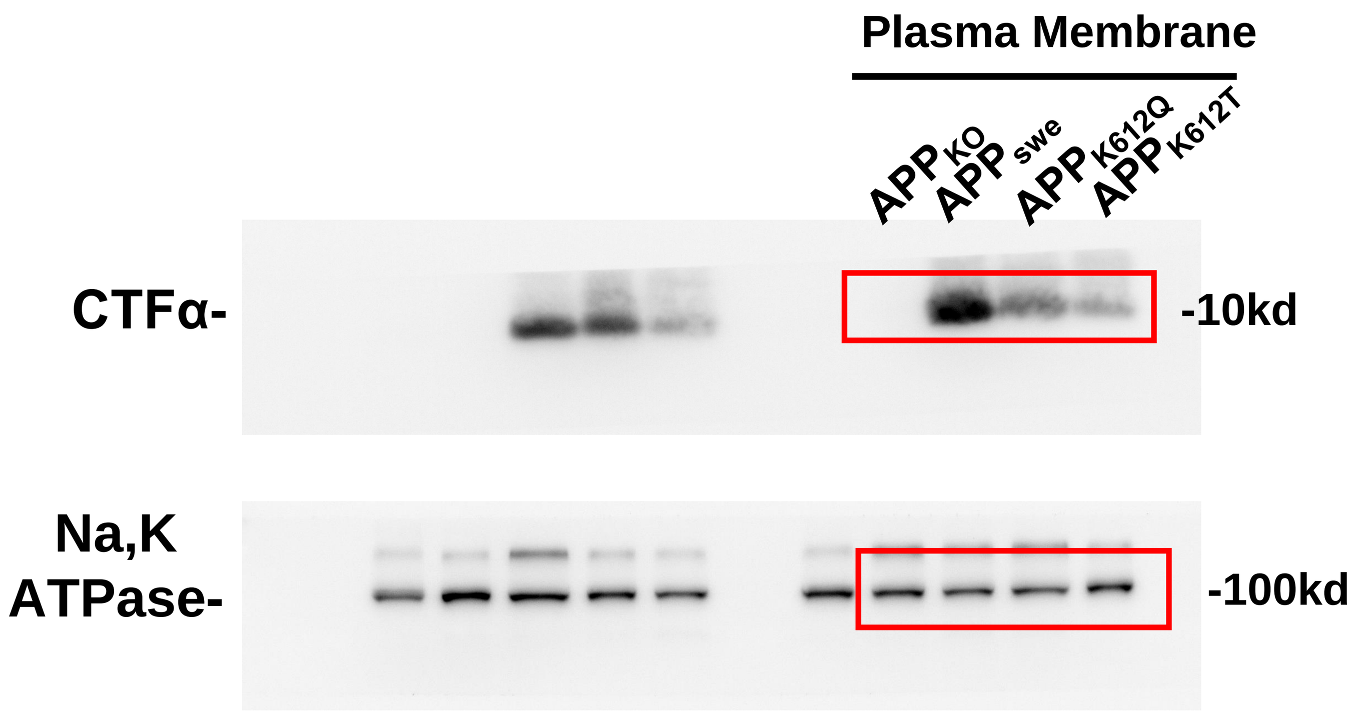

Supplemental Figure 3A

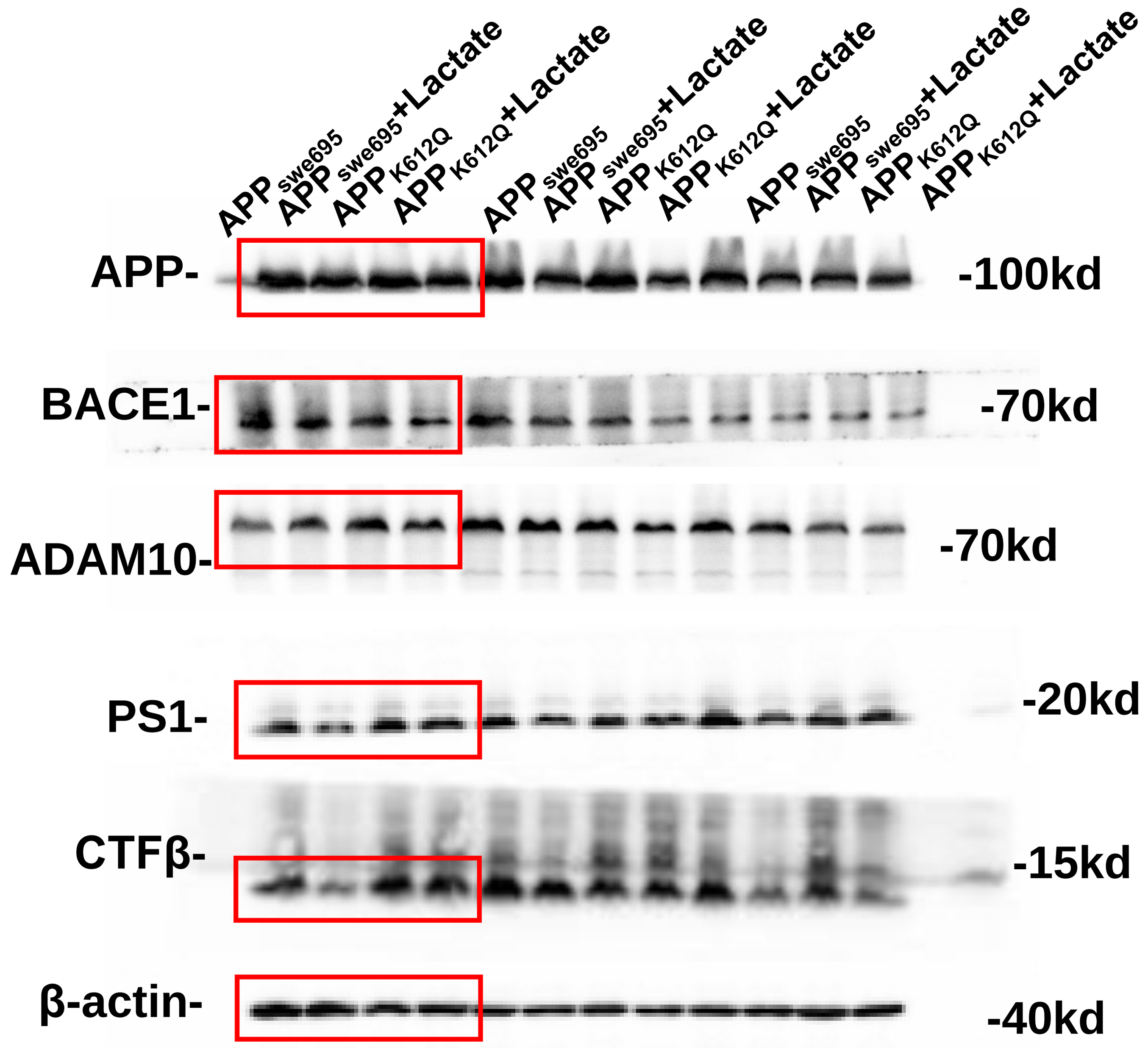

Supplemental Figure 4B

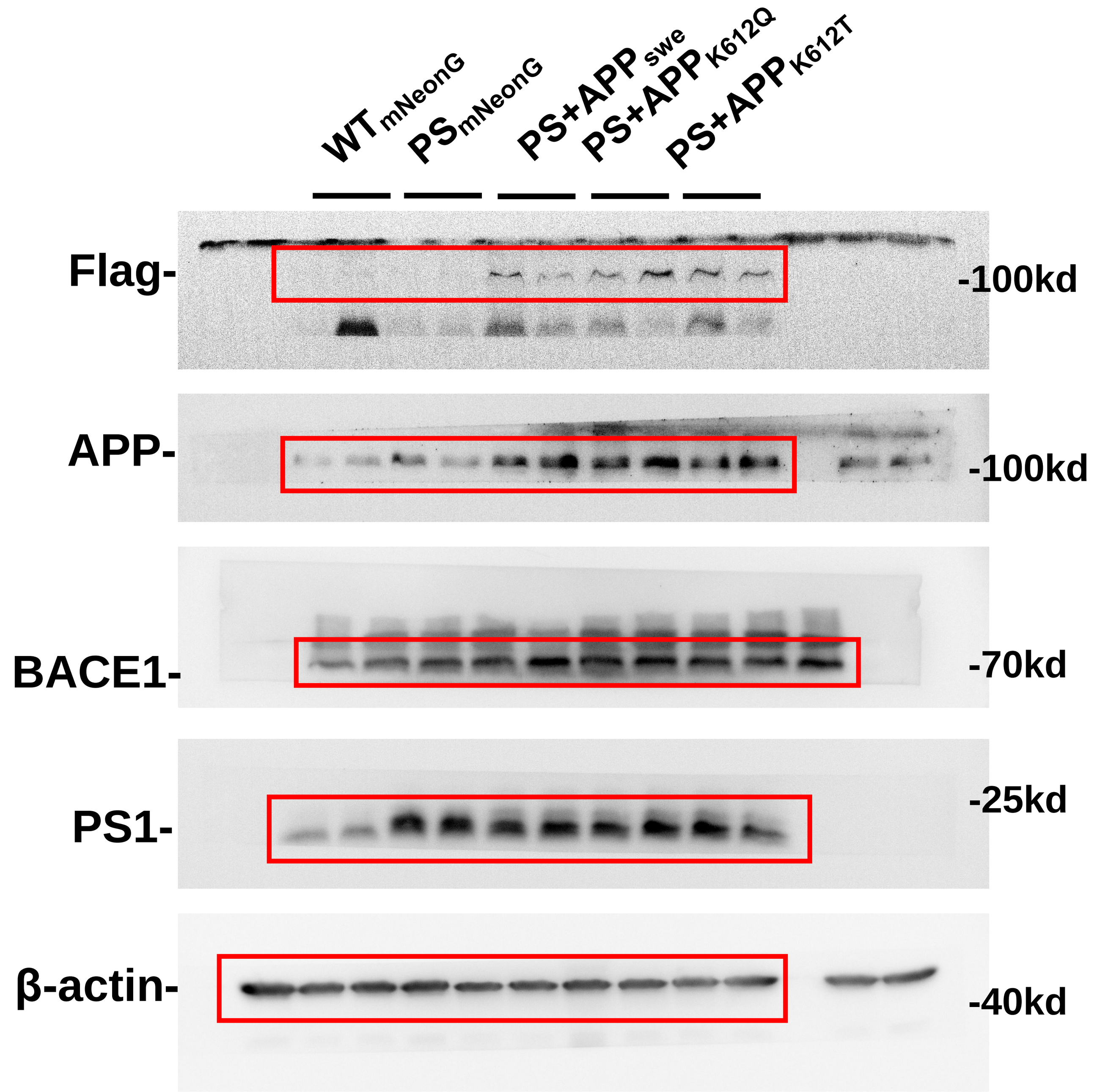

Supplemental Figure 7

A

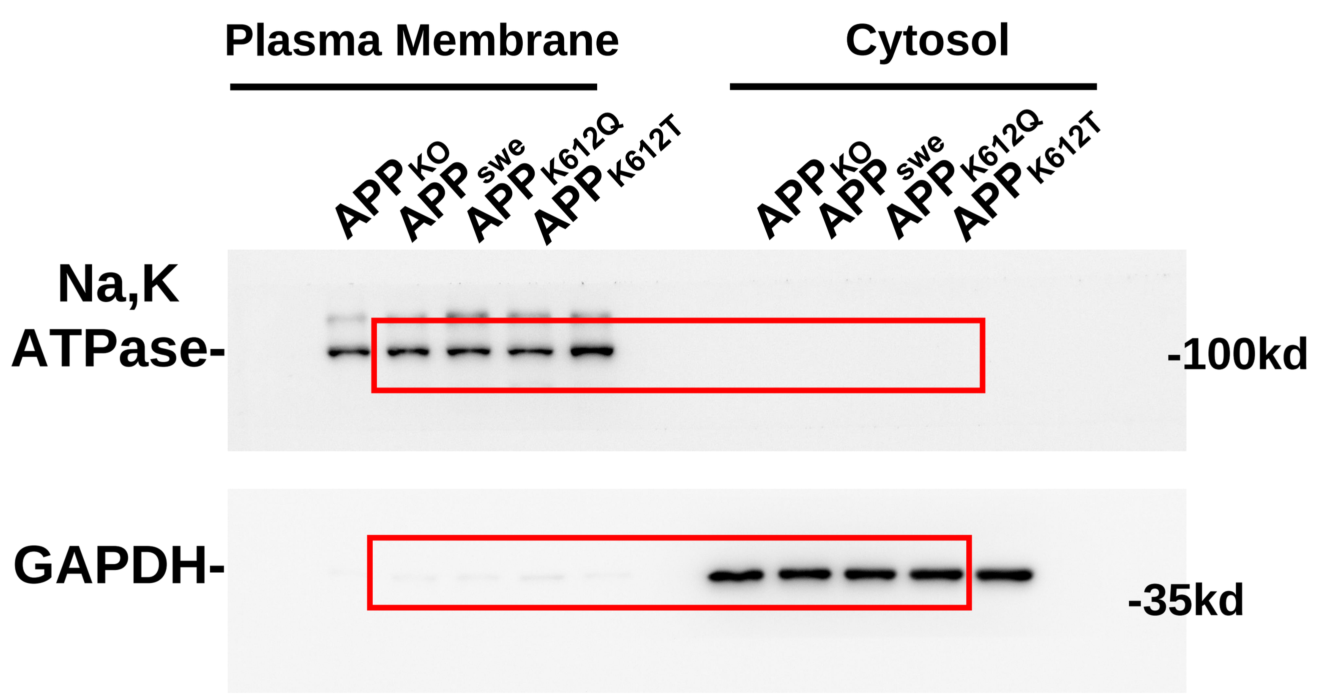

B

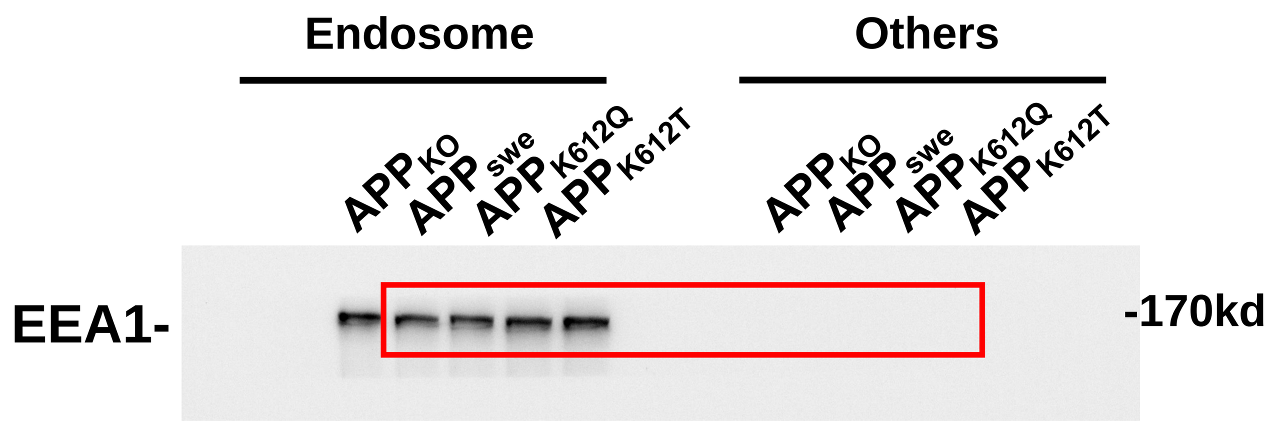

C

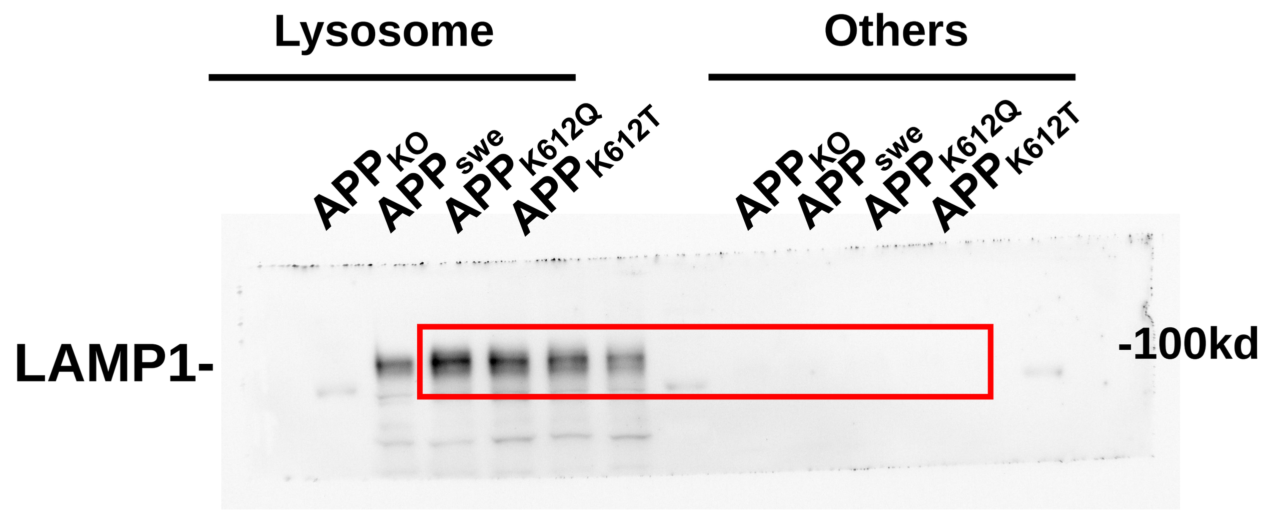

Supplemental Figure 8A

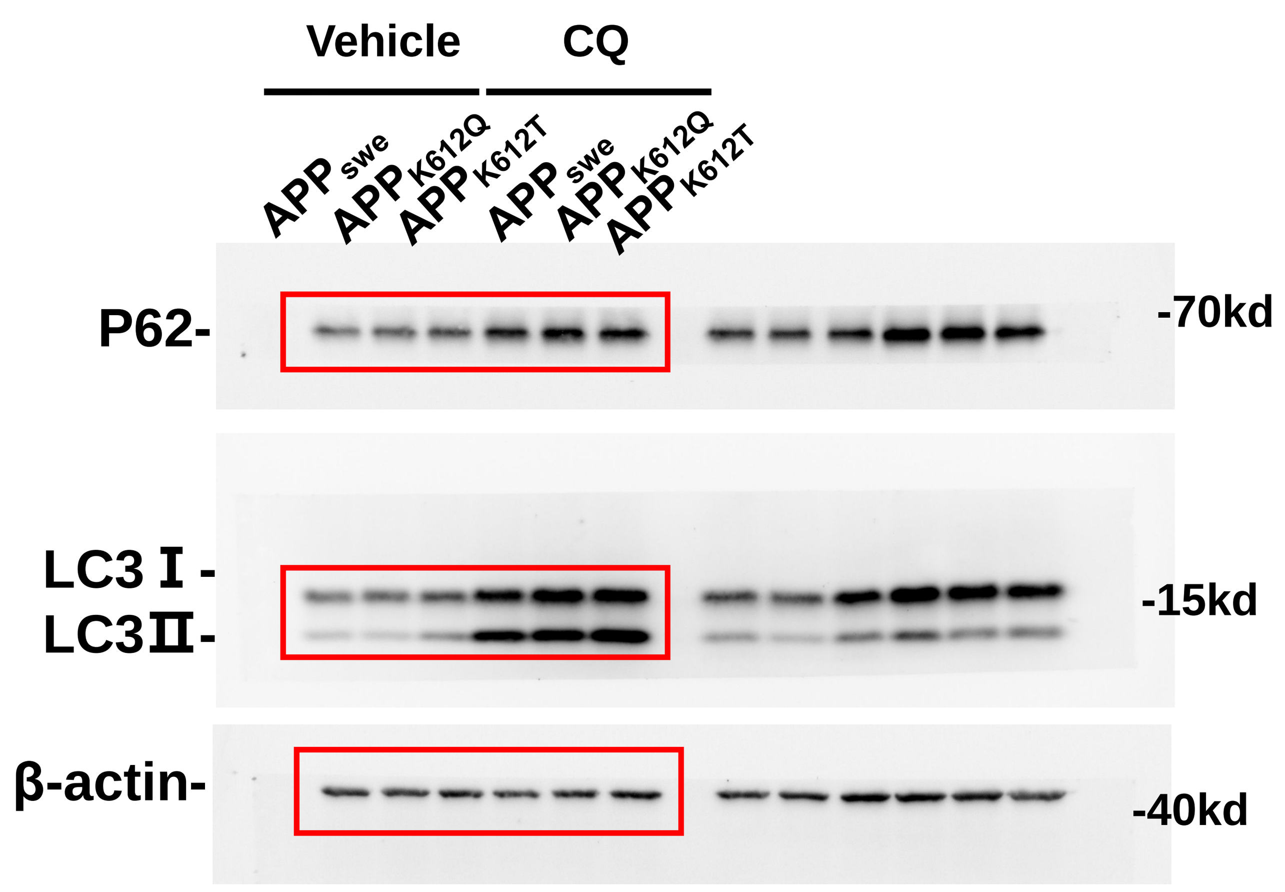

Supplemental Figure 8D

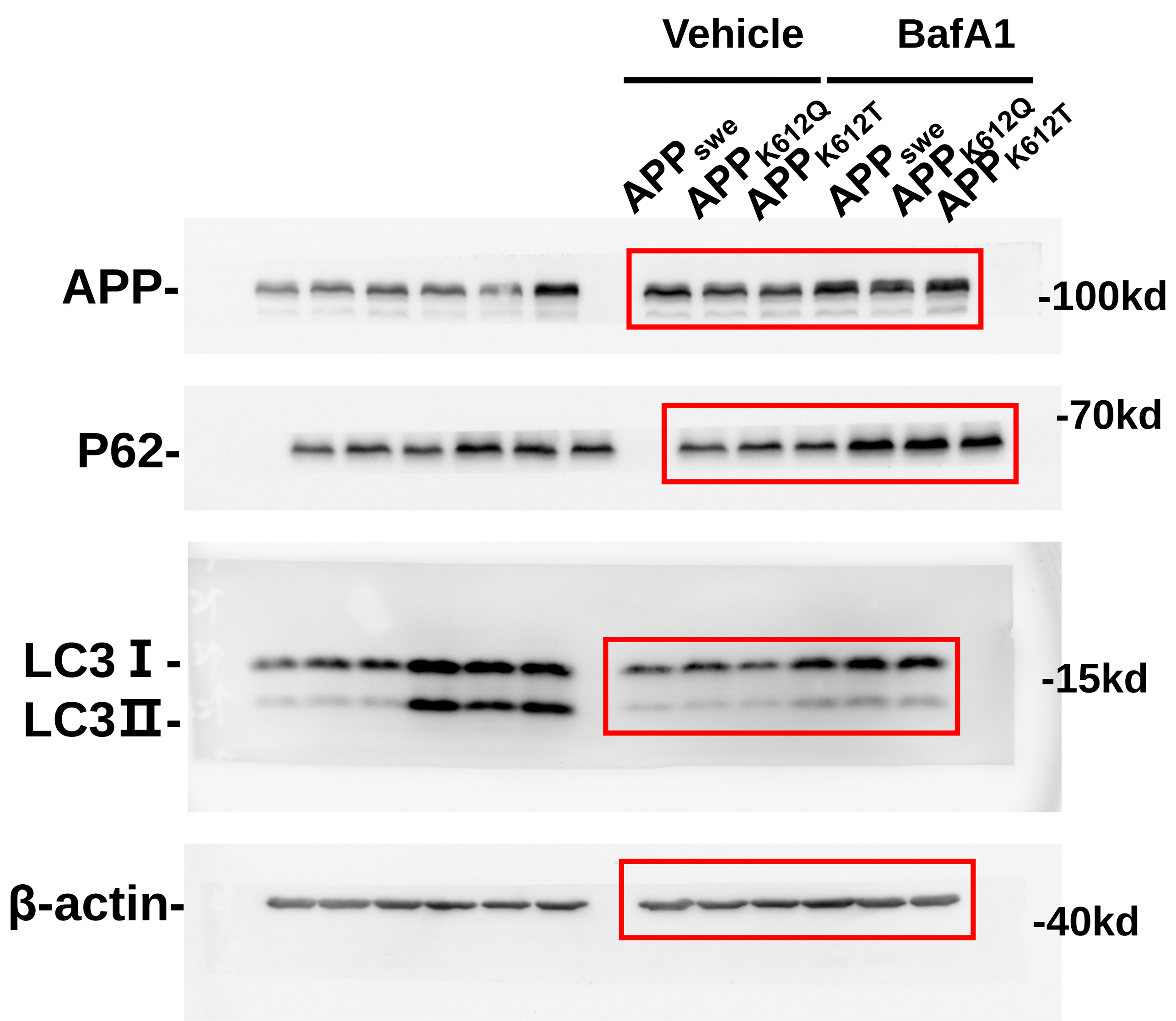

Supplemental Figure 10B

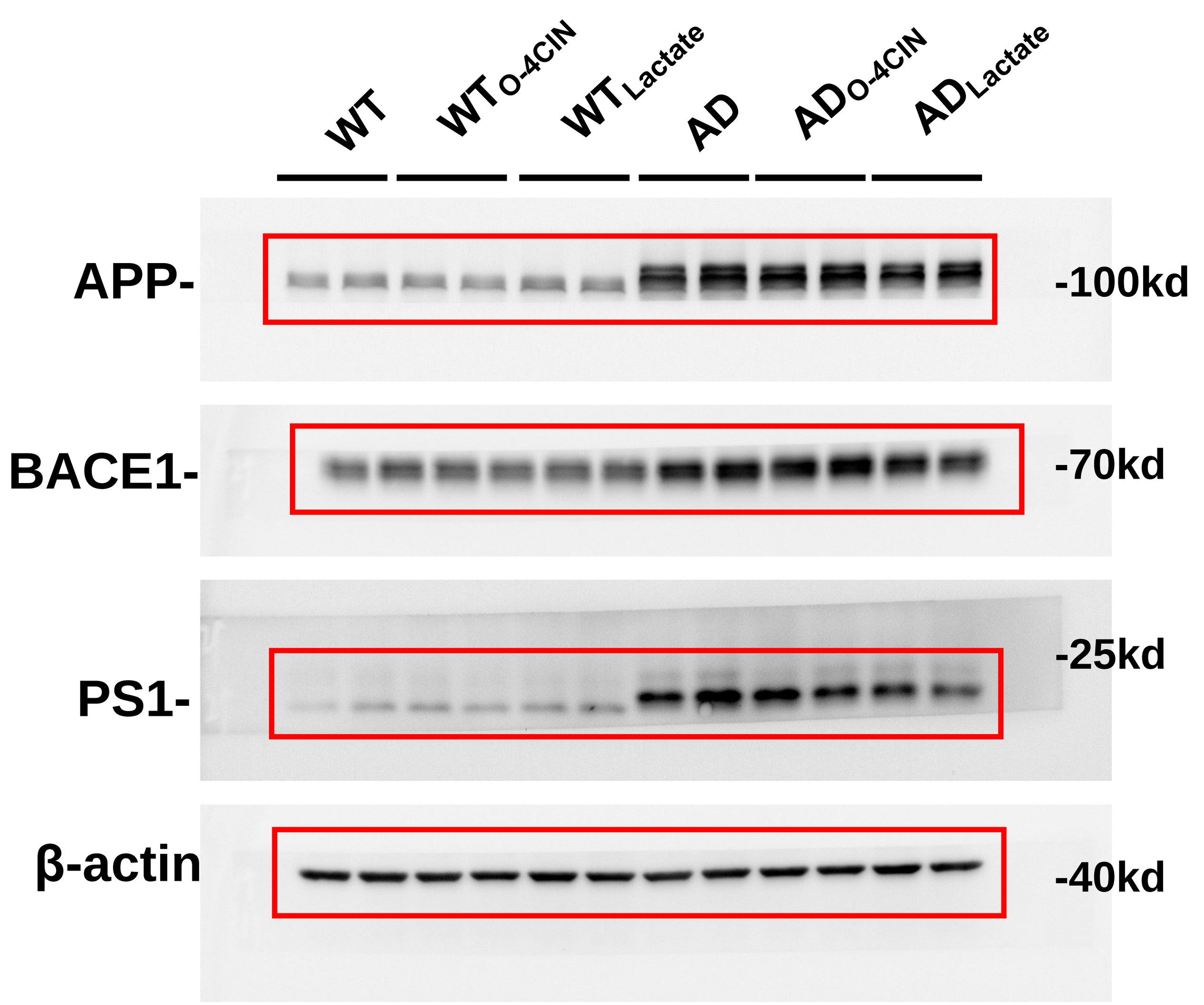

Supplement: Unedited blot and gel images [file jci-135-184656-s083.pdf]
